# Synthesis of 3-Aryl-*ortho*-carboranes with Sensitive Functional Groups †

**DOI:** 10.3390/molecules26237297

**Published:** 2021-12-01

**Authors:** Sergey A. Anufriev, Akim V. Shmal’ko, Kyrill Yu. Suponitsky, Igor B. Sivaev

**Affiliations:** A.N. Nesmeyanov Institute of Organoelement Compounds, Russian Academy of Sciences, 28 Vavilov Str., 119991 Moscow, Russia; trueman476@mail.ru (S.A.A.); akimbend@yandex.ru (A.V.S.); kirshik@yahoo.com (K.Y.S.)

**Keywords:** *ortho*-carborane, 3-aryl derivatives, synthesis, Co/Pd catalysis, X-ray diffraction, intramolecular hydrogen bonds

## Abstract

A simple and efficient method was developed for the one-pot synthesis of 3-aryl derivatives of *ortho*-carborane with sensitive functional groups using 3-iodo-*ortho*-carborane and aryl zinc bromides that were generated in situ. A series of 3-aryl-*ortho*-carboranes, including those containing nitrile and ester groups, 3-RC_6_H_4_-1,2-C_2_B_10_H_11_ (R = *p*-Me, *p*-NMe_2_, *p*-OCH_2_OMe, *p*-OMe, *o*-CN, *p*-CN, *o*-COOEt, *m*-COOEt, *p*-COOEt) was synthesized using this approach. The solid-state structures of 3-RC_6_H_4_-1,2-C_2_B_10_H_11_ (R = *p*-OMe, *o*-CN, and *p*-CN) were determined by single crystal X-ray diffraction. The intramolecular hydrogen bonding involving the *ortho*-substituents of the aryl ring and the CH and BH groups of carborane was discussed.

## 1. Introduction

Aryl derivatives of icosahedral carboranes C_2_B_10_H_12_ are of interest for a variety of applications, from the development of new materials [1,2,3,4,5,6,7,8,9,10,11,12] to the design of pharmaceuticals [13,14,15,16,17]. This dictates the need to develop convenient methods for their synthesis. Methods for the synthesis of *C*-arylcarboranes have been well developed and are widely used in the synthesis of a wide variety of aryl derivatives [18]. The general method includes the reaction of decaborane *nido*-B_10_H_14_ with Lewis bases L (L = SR_2_, NR_3_, MeCN), resulting in the 6,9-*arachno*-B_10_H_12_L_2_ derivatives. These derivatives react with arylacetylenes to form the corresponding *C*-aryl-*ortho*-carboranes [19]. However, this reaction gives very low yields when used with some sterically hindered alkynes, especially those containing two aromatic moieties [10,20,21,22,23,24,25], and it cannot be used with arylacetylenes that have acidic or easily reducible substituents. This method is also unsuitable for use in the synthesis of the aryl derivatives of *meta*- and *para*-carboranes. Therefore, for the synthesis of aryl derivatives of *meta*- and *para*-carboranes, the Ullmann-type copper-coupling reactions are used. In this way, *C*-mono and *C,C’*-diaryl derivatives of *meta*- and *para*-carboranes can be obtained, as well as *C*-aryl derivatives of *ortho*-carborane [20,26,27,28,29,30]. An alternative method is based on the Ni-catalyzed cross-coupling reactions of aryl iodides with carboranyl Grignard reagents. In this way, both monoaryl and diaryl derivatives of *ortho*-carborane can be prepared [31,32,33].

The synthesis of the *B*-aryl derivatives of carboranes is mainly based on the Pd-catalyzed cross-coupling reactions of their iodo derivatives with aryl Grignard reagents (Kumada cross-coupling). In this way, various 9-aryl and 9,12-diaryl derivatives of *ortho*-carborane, 9-aryl and 9,10-diaryl derivatives of *meta*-carborane, and 2-aryl derivatives of *para*-carborane were synthesized [34,35,36,37,38,39,40,41,42]. However, the available substituents on the aromatic ring in these reactions are strictly limited due to the high reactivity of the Grignard reagents. Mild *B*-arylation of carboranes via the Suzuki cross-coupling reactions of aryl boronic acids with 9-iodo-*meta*- and 2-iodo-*para*-carboranes were reported [43,44]. These cross-coupling reactions can be used for the direct introduction of functionalized aryl substituents that are not compatible with the Kumada reaction conditions. However, this approach turned out to be ineffective for *ortho*-carborane because in order to facilitate the transmetallation step in the Suzuki cross-coupling reactions, inorganic bases such as F^−^ or OH^−^ are usually used, which are strong nucleophiles that can lead to the deboronation of the *ortho*-carborane cage.

4,5-Diphenyl- and 3,6-diphenyl-*ortho*-carboranes were obtained from the Pd- and Rh-catalyzed B-H activation reactions that started from *ortho*-carborane derivatives containing removable carboxylic- [45] and imine-directing [46] functional groups, respectively. 3,6-Diphenyl-*ortho*-carborane was prepared as well by the Ir-catalyzed borylation of *ortho*-carborane via direct B-H activation followed by the Pd-catalyzed Suzuki cross-coupling of the resulting 3,6-(Bpin)_2_-*ortho*-carborane with phenyl bromide [47]. 3-Phenyl-*ortho*-carborane and some its *C*-substituted analogues can be obtained via the insertion of a BPh fragment into the *nido*-carborane cage by the reaction with PhBCl_2_ under basic conditions [48,49,50]. However, this approach cannot be applied to the synthesis of a wide range of 3-aryl derivatives due to the practical unavailability of many ArBCl_2_ reagents, as well as because of their high reactivity, which excludes the use of aryls with sensitive functional groups. 3-Phenyl- and 3-(9-anthracenyl)-*ortho*-carboranes were prepared by the Pd-catalyzed cross-coupling of 3-iodo-*ortho*-carborane with the corresponding aryl Grignard reagents [51]. However, the range of available substituents on the aromatic ring in these reactions is also very limited due to the high reactivity of the Grignard reagents. Moreover, it was found that reactions 3-iodo-*ortho*-carborane with organometallic reagents, which are “hard” nucleophiles, in the presence of a catalytic amount of [Pd(PPh_3_)_4_] can lead to the loss of a halogen with the formation of 1,3-dehydro-*ortho*-carboryne [52], whereas the reaction with an equimolar amount of [Pd(PPh_3_)_4_] in the presence of K_2_CO_3_ in DMF proceeds with the decapitation of the carborane cage resulting in *nido*-carborane [7,8-C_2_B_9_H_12_]^−^ as the final product [53]. The preparation of 3-phenyl-*ortho*-carborane by the reaction of the diazonium derivative of *ortho*-carborane [3-N_2_-1,2-C_2_B_10_H_11_]BF_4_ with the Grignard reagent was reported [54]; however, in our hands these reactions led exclusively to the 3-arylazo derivatives of *ortho*-carborane [55]. The attempt to use the Suzuki cross-coupling reaction of 3-iodo-*ortho*-carborane with aryl boronic acids only gave good results for aryls containing electron-donating substituents, while reactions with aryl boronic acids containing electron-withdrawing substituents (-CN, -NO_2_) only led to the desired carboranes in low yields [56]. Recently, the direct arylation of *ortho*-carborane via Pd-catalyzed B-H activation has been reported, but this approach has been optimized for the synthesis of the 3,6-diaryl derivatives rather than the 3-aryl derivatives [57]. A series of 3-aryl derivatives of *ortho*-carborane were prepared by Pd-catalyzed B-H activation reactions with aryl iodides under functional group assistance; however, these reactions are currently only of academic interest rather than real synthetic methods [58,59]. Recently, we proposed a convenient and mild one-pot method for the synthesis of 9-aryl- and 9,12-diaryl-*ortho*-carboranes with sensitive functional groups, including esters and nitriles, using sequential Co- and Pd-catalyzed reactions [60].

In this contribution, we describe the application of this method for the synthesis of a series of 3-aryl-*ortho*-carboranes, including those containing sensitive functional groups.

## 2. Results and Discussion

The method proposed is based on the mild generation of aryl zinc reagents followed by their Pd-catalyzed cross-coupling with 3-iodo-*ortho*-carborane. The aryl zinc reagents were prepared via the Co-catalyzed reaction of aryl bromides containing various functional groups with zinc dust [61,62,63]. The organozinc compounds that are obtained in this way can be easily coupled with various aryl iodides in the presence of a catalytic amount of (Ph_3_P)_2_PdCl_2_ [63]. Previously, we had successfully used this approach for the synthesis of 9-aryl-*ortho*-carboranes containing functional groups that were sensitive to organolithium and organomagnesium reagents [60].

**Scheme 1:**
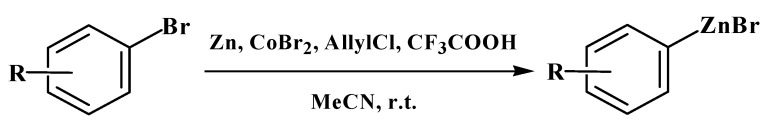
In situ synthesis of aryl zinc bromides.

Aryl zinc bromides containing various substituents, including sensitive functional groups (-CN, -COOEt), were prepared by the reaction of the corresponding aryl bromides with allyl zinc chloride/bromide that was generated from allyl chloride and zinc metal in the presence of 25 mol.% of CoBr_2_ and a catalytic amount of trifluoroacetic acid in acetonitrile at ambient temperature (Scheme 1).

**Scheme 2:**
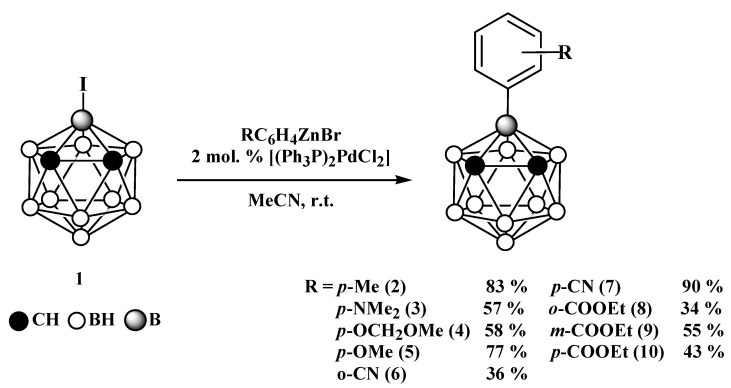
Synthesis of 3-aryl-*ortho*-carboranes.

The reactions of the prepared aryl zinc bromides with 3-iodo-*ortho*-carborane in the presence of 2 mol.% of [(Ph_3_P)_2_PdCl_2_] in acetonitrile at room temperature results in the corresponding 3-aryl-*ortho*-carboranes (Scheme 2).

The synthesized 3-aryl-*ortho*-carboranes were characterized by ^1^H, ^13^C and ^11^B NMR spectroscopy. The ^1^H and ^13^C NMR spectra of all compounds contain signals of the corresponding aryl substituents as well as the signals of the carborane cage. It should be noted that the signals of the aromatic hydrogens in the ^1^H NMR spectra of 3-aryl-*ortho*-carboranes in CDCl_3_ are noticeably shifted to the downfield region in comparison with the signals of the corresponding 9-aryl-*ortho*-carboranes [60], with a difference in the weighted average chemical shift of aromatic hydrogens in the range of 0.11 to 0.34 ppm. This is in agreement with the transition from the markedly electron-donating *ortho*-carboran-9-yl group to the slightly electron-accepting *ortho*-carboran-3-yl group [64]. In general, the chemical shift of the carborane *CH* groups depends slightly on the presence of electron-donating (3.65–3.69 ppm) or electron-accepting (3.74–3.77 ppm) substituents in the aromatic ring. However, most notable is the strong downfield shift of the *CH* carborane signals in the case of *ortho*-substituted aryl groups (4.38 and 4.37 ppm for 3-(2′-EtOOCC_6_H_4_)-1,2-C_2_B_10_H_11_ (**8**) and 3-(2′-NCC_6_H_4_)-1,2-C_2_B_10_H_11_ (**6**), respectively). In the first case, it can be explained by the formation of a hydrogen bond between the carborane *CH* group and the ester carbonyl group. It is known that the chemical shifts of the *CH* groups of carboranes and metallacarboranes are very sensitive to the formation of intramolecular hydrogen bonds [65,66]. On the other hand, the signals of the carbonyl group in the ^13^C NMR spectra are also sensitive to the formation of hydrogen bonds [67,68]. Indeed, the signal of the carbonyl group in 3-(2′-EtOOCC_6_H_4_)-1,2-C_2_B_10_H_11_ (**8**) (170.0 ppm) demonstrates a significant downfield shift compared to those found in 3-(3′-EtOOCC_6_H_4_)-1,2-C_2_B_10_H_11_ (**9**) (166.5 ppm) and 3-(4′-EtOOCC_6_H_4_)-1,2-C_2_B_10_H_11_ (**10**) (166.4 ppm), as well as in 9-(2′-EtOOCC_6_H_4_)-1,2-C_2_B_10_H_11_ (161.5 ppm) [60], which is a clear confirmation of the formation of an intramolecular hydrogen bond. It is also worth noting that the mass spectrum of **8** in the negative mode, in contrast to the mass spectra of other 3-aryl-*ortho*-carboranes, exhibits a peak which, in addition to the loss of the carborane proton, corresponds to the abstraction of the ethanol molecule. This is caused by the attack of the carbonyl group by the nucleophile, which is formed upon the loss of the carborane *CH* proton, leading to intramolecular cyclization with the elimination of ethanol and the formation of carboranyl fluorenone 1,3-µ-C(O)C_6_H_4_-1,2-C_2_B_10_H_10_. In the case of 3-(2′-NCC_6_H_4_)-1,2-C_2_B_10_H_11_, the nature of the interactions involving the carborane *CH* group is less clear.

**Figure 1:**
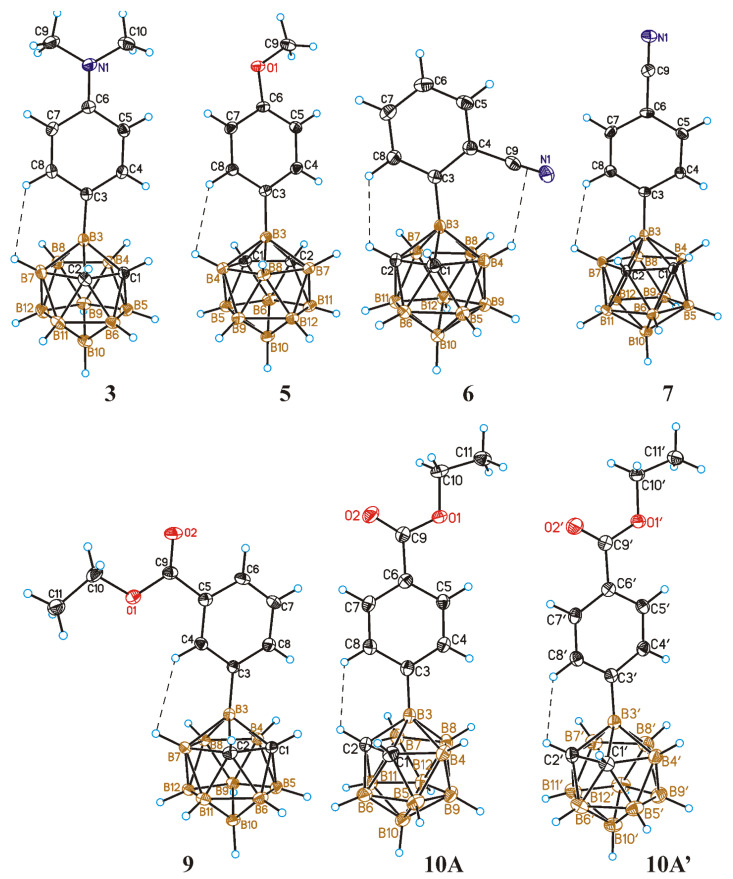
General views of 3-(*p*-Me_2_NC_6_H_4_)-1,2-C_2_B_10_H_11_ (**3**), 3-(*p*-MeOC_6_H_4_)-1,2-C_2_B_10_H_11_ (**5**), 3-(*o*-NCC_6_H_4_)-1,2-C_2_B_10_H_11_ (**6**), 3-(*p*-NCC_6_H_4_)-1,2-C_2_B_10_H_11_ (**7**), 3-(*m*-EtOOCC_6_H_4_)-1,2-C_2_B_10_H_11_ (**9**), and 3-(*p*-EtOOCC_6_H_4_)-1,2-C_2_B_10_H_11_ (**10**) (both symmetrically independent molecules are presented) showing atomic numbering. Thermal ellipsoids are drawn at 50% probability level. For each structure, the shortest H⋯H and H⋯π contacts are shown by dashed lines. The H⋯H distances are equal to 2.43, 2.43, 2.18, 2.44, 2.47, 2.27, and 2.37 Å for compounds **3**, **5**, **6**, **7**, **9**, **10**A, and **10**A′, respectively. The distance of the H⋯π contact for **6** (from H atom δ to the center of the C≡N bond) is equal to 2.80(2) Å.

This prompted us to perform an X-ray diffraction study of the synthesized 3-aryl-*ortho*-carboranes. The solid-state structures of carboranes **3**, **5**–**7**, **9** and **10** were determined by single crystal X-ray diffraction (Figure 1). The most characteristic feature of the structure of 3-aryl-*ortho*-carboranes is the deviation of the exo-polyhedral B-C bond from the B(3)-B(10) axis of the carborane cage towards the carborane C(1)-C(2) bond (See Table 1).

This can be related to the fact that the B(3)-C bonds are somewhat shorter than the B(3)-B bonds, as well as to the participation of the aryl substituents in intermolecular interactions. The orientation of the aryl ring with respect to the carborane cage in the obtained derivatives is different. For compounds **3**, **5**, **7**, and **9**, the projection line of the phenyl ring onto the C_2_B_3_ plane passes through the B(7) or B(4) atom and the center of the opposite B-C bond, while for compounds **6** and **10**, it passes through the C(2) atom and the center of the B(4)-B(8) bond. As shown in Figure 1, a C_phen_-H⋯H-B(C) contact is observed in all of the compounds. In some cases, it is slightly longer than the sum of the van-der-Waals radii (2.4 Å [69]), while it is somewhat shorter in the other cases. The shortest distance (2.18 Å) is observed for compound **6** with an *ortho*-cyanophenyl substituent. In structures **5** and **7**, there are no short contacts between the aryl ring and the carborane cage, while in structure **6** the aryl ring is rotated in such a way that leads to the formation of a short (2.174 Å) C(2)H⋯HC(8) contact between the aryl and carborane hydrogens. It should be noted that the presence of such contacts was previously found in the structure of 1-phenyl-*ortho*-carborane [70]. In addition, there is a slightly shortened contact of the B-H⋯π(N≡C-) type between the B(4)-H group of carborane and the cyano group of the aryl substituent. It should be mentioned that the existence of the intramolecular B-H⋯π(N≡C-) hydrogen bonds in the *nido*-carborane derivative 10-N≡CCH_2_(Me)S-7,8-C_2_B_9_H_11_ was postulated earlier based on the NMR spectroscopy data [71].

**Figure 2:**
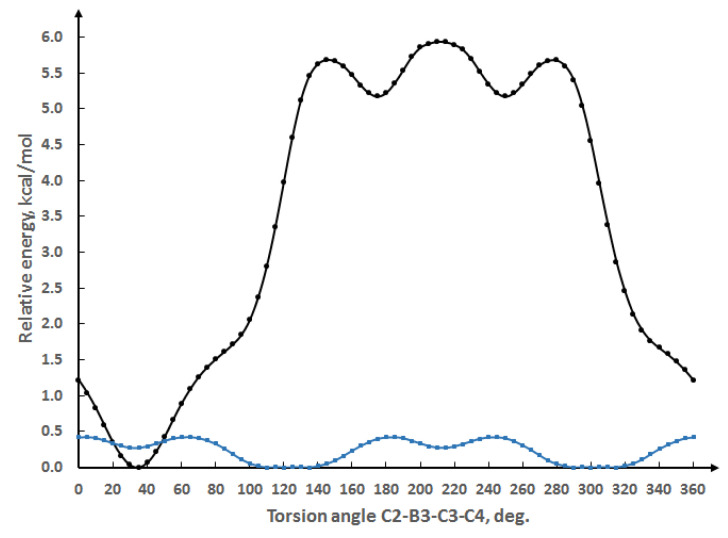
Dependence of conformational energy on C(2)-B(3)-C(3)-C(4) torsion angle for compounds **6** (black curve) and **7** (blue curve).

In order to understand in more detail the observed differences in molecular conformation, we carried out a comparative quantum chemical study for compounds **7** and **6,** which have *para*- and *ortho*-cyanophenyl substituents and significantly differ in their molecular conformation and intramolecular contacts. The calculation was done using the GAUSSIAN program [72] at the PBE0/def2tzvp level of theory that was shown to provide realistic geometrical and energetic properties for different types of compounds [73,74,75]. In order to search for preferential molecular conformation, the aryl substituent was rotated about the B(3)-C(3) bond with a step of 5°. The results are shown in Figure 2.

**Figure 3:**
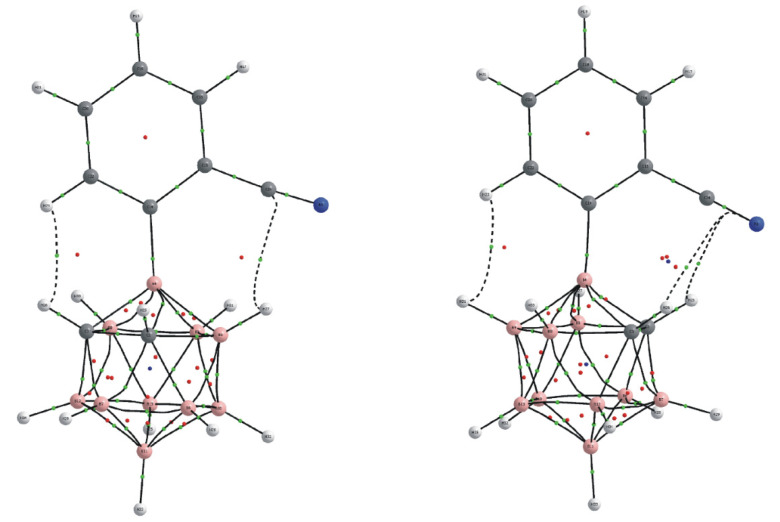
View of the molecular conformations corresponding to local (**left**) and global (**right**) minima for compound **6**.

As expected, for the *para*-cyanophenyl derivative (**7**), the barrier to rotation is small and the conformational curve is symmetrical with respect to the plane passing through the B(3) and B(8) atoms and the center of the C(1)-C(2) bond. The experimental structure corresponds to the global minimum. In the case of the *ortho*-cyanophenyl derivative (**6**), there are two equivalent local minima and a global minimum, which probably corresponds to structure **6** in solution, and which is ~5 kcal/mol lower in energy. Using the geometries of the local and global minima as starting points, we carried out an additional optimization without any restrictions. Both optimizations converged to true minima, global and local, respectively. The QTAIM theory [76] was utilized to analyze the intramolecular noncovalent interactions. A search for the bond critical points (using the AIMALL program [77]) revealed the presence of two intramolecular attractive interactions between the carborane cage and the aryl substituent for both local and global minima (Figure 3). The energies of the observed noncovalent interactions were estimated using the empirical correlation between interaction energy and potential energy density at the bond critical point (*E* = 1/2*V*(*r*)) [78], which is frequently utilized for energetic analysis [73,79,80].

The stabilization of the global minimum was provided by the B-H⋯H-C_phen_ (−1.6 kcal/mol) and C_carb_-H⋯π (−3.8 kcal/mol) nonbonded interactions, while C_carb_-H⋯H-C_phen_ (−2.4 kcal/mol) and B-H⋯π (−1.3 kcal/mol) contacts were observed for the local minimum which, evidently, was weaker. At the same time, the experimentally observed solid-state structure of compound **6** corresponds to the local minima which should be caused by the crystal-packing influence. Indeed, in the crystal structure of **6**, a relatively strong intermolecular hydrogen bond C(2)-H(2)⋯N(1) is formed (Figure S1 in the Supplementary Materials). Such an interaction cannot exist for a molecular conformation corresponding to the global minima due to steric reasons.

Based on these results we can suggest that experimental molecular conformation of compound **10**, for which intramolecular C_phen_-H⋯H-C_carb_ shortened contacts are observed, is also influenced by the crystal-packing effect (Figure S2 in the Supplementary Materials).

In summary, the efficient method for the one-pot synthesis of 3-aryl-*ortho*-carboranes with sensitive functional groups using sequential Co- and Pd-catalyzed reaction was proposed. A series of functional aryl derivatives, including esters and nitriles, were synthesized and characterized by methods of the NMR spectroscopy and single crystal X-ray diffraction.

## 3. Experimental Part

### General Synthetic Procedure and Characterization of 3-Aryl-ortho-carboranes

Allyl chloride (82 μL, 77 mg, 1.00 mmol) and trifluoroacetic acid (25 μL, catalytic amount) were added to a blue mixture of zinc powder (490 mg, 7.50 mmol) and anhydrous cobalt dibromide (55 mg, 0.25 mmol) in 2.5 mL of fresh distilled acetonitrile. The resulting dark orange mixture was stirred at room temperature for 15 min. Then corresponding aryl bromide (2.50 mmol) was added, and reaction was stirred at room temperature for an additional 1 h. Then, 3-iodo-*ortho*-carborane (**1**) (270 mg, 1.00 mmol) with bis(triphenylphosphine)palladium dichloride (14 mg, 0.02 mmol) were added. The reaction was stirred at room temperature overnight. After the removal of volatiles under reduced pressure, the residue was washed with water (25 mL), dichloromethane (3 × 25 mL) and acetone (until no trace of carborane appeared on TLC). The organic phases were combined, dried over Na_2_SO_4_ and concentrated under reduced pressure. The crude product was purified by column chromatography on silica to give the corresponding 3-aryl-*ortho*-carborane.

3-(4′-Methylphenyl)-*ortho*-carborane (**2**): 4-methylphenyl bromide (315 μL, 435 mg, 2.50 mmol) was used; diethyl ether was used as the eluent for column chromatography; a pale-yellow crystalline solid was obtained (195 mg, yield 83%). ^1^H NMR (400 MHz, CDCl_3_): δ 7.49 (2H, d, J = 7.7 Hz, C*H_Ar_*), 7.19 (2H, d, J = 7.7 Hz, C*H_Ar_*), 3.69 (2H, br s, C*H_Carb_*), 2.37 (3H, s, C*H_3_*) ppm; ^11^B NMR (128 MHz, CDCl_3_): δ −2.4 (2B, d, J = 148 Hz), −5.0 (1B, s, *B*-C), −8.5 (1B, d, J = 145 Hz), −12.9 (3B, d, J = 162 Hz), −13.7 (3B, d, J = 174 Hz) ppm; ^13^C NMR (100 MHz, CDCl_3_): δ 139.7 (*C_Ar_*-CH_3_), 133.2 (*C_Ar_*H), 129.2 (*C_Ar_*H), 56.8 (*C_Carb_*H), 21.5 (*C*H_3_) ppm. MS (DUIS): *m*/*z* for C_9_H_18_B_10_: calcd. 233.2 [M−H]^−^, obsd. 233.2 [M−H]^−^.

3-(4′-*N*,*N*-Dimethylaminophenyl)-*ortho*-carborane (**3**): 4-*N*,*N*-dimethylaminophenyl bromide (500 mg, 2.50 mmol) was used; a mixture of chloroform and hexane (3:1, *v*/*v*) was used as the eluent for column chromatography; a pale-pink crystalline solid was obtained (150 mg, yield 57%). ^1^H NMR (400 MHz, CDCl_3_): δ 7.46 (2H, d, J = 8.5 Hz, C*H_Ar_*), 6.72 (2H, d, J = 8.5 Hz, C*H_Ar_*), 3.65 (2H, br s, C*H_Carb_*), 3.00 (6H, s, N(C*H_3_*)_2_) ppm; ^11^B NMR (128 MHz, CDCl_3_): δ −2.7 (2B, d, J = 149 Hz), −4.0 (1B, s, *B*-C), −8.6 (1B, d, J = 150 Hz), −13.1 (3B, d, J = 152 Hz,), −13.7 (3B, d, J = 164 Hz) ppm; ^13^C NMR (100 MHz, CDCl_3_): δ 151.5 (*C_Ar_*-N), 134.3 (*C_Ar_*H), 117.4 (*C_Ar_*-B), 112.0 (*C_Ar_*H), 56.8 (*C_Carb_*H), 40.3 (N(*C*H_3_)_2_) ppm. MS (DUIS): *m*/*z* for C_10_H_21_B_10_N: calcd. 262.3 [M−H]^−^, obsd. 262.3 [M−H]^−^; calcd. 305.3 [M+H+MeCN]^+^, obsd. 305.3 [M+H+MeCN]^+^.

3-(4′-Methoxymethoxyphenyl)-*ortho*-carborane (**4**): 4-methoxymethoxy bromide (381 μL, 543 mg, 2.50 mmol) was used; a mixture of chloroform and hexane (3:1, *v*/*v*) and a mixture of diethyl ether and hexane (1:2, *v*/*v*) were used as the eluent for column chromatography; a pale-yellow solid was obtained (164 mg, yield 58%). ^1^H NMR (400 MHz, CDCl_3_): δ 7.52 (2H, d, J = 8.5 Hz, C*H_Ar_*), 7.04 (2H, d, J = 8.5 Hz, C*H_Ar_*), 5.20 (2H, s, OC*H_2_*O), 3.67 (2H, br s, C*H_Carb_*), 3.48 (3H, s, OC*H_3_*) ppm; ^11^B NMR (128 MHz, CDCl_3_): δ −2.5 (2B, d, J = 149 Hz), −4.8 (1B, s, *B*-C), −8.5 (1B, d, J = 150 Hz), −13.0 (3B, d, J = 156 Hz), −13.6 (3B, d, J = 159 Hz) ppm; ^13^C NMR (100 MHz, CDCl_3_): δ 158.7 (*C_Ar_*-O), 134.6 (*C_Ar_*H), 122.7 (*C_Ar_*-B), 116.2 (*C_Ar_*H), 94.2 (O*C*H_2_O), 56.8 (*C_Carb_*H), 56.2 (O*C*H_3_) ppm. MS (DUIS): *m*/*z* for C_10_H_20_B_10_O_2_: calcd. 279.2 [M−H]^−^, obsd. 279.3 [M−H]^−^. Crystallographic data (CCDC number 2124596): C_10_H_21_B_10_N are monoclinic, space group *P2*_1_/*c*: *a* = 12.9798(5) Å, *b* = 9.8799(4) Å, *c* = 12.5908(5) Å, *β* = 109.130(2)°, *V* = 1525.47(11) Å^3^, *Z* = 4, *M* = 263.38, *d*_cryst_ = 1.147 g·cm^−3^. *wR*_2_ = 0.1159 calculated on *F*^2^*_hkl_* for all 3666 independent reflections with 2*θ* < 56.0°, (*GOF* = 1.074, *R* = 0.0417 calculated on *F_hkl_* for 2986 reflections with *I* > 2*σ*(*I*)).

3-(4′-Methoxyphenyl)-*ortho*-carborane (**5**): 4-methoxyphenyl bromide (312 μL, 468 mg, 2.50 mmol) was used; a mixture of diethyl ether and hexane (1:2, *v*/*v*) was used as the eluent for column chromatography; a yellow solid was obtained (193 mg, yield 77%). ^1^H NMR (400 MHz, CDCl_3_): δ 7.52 (2H, d, J = 8.6 Hz, C*H_Ar_*), 6.90 (2H, d, J = 8.6 Hz, C*H_Ar_*), 3.83 (3H, s, OC*H_3_*), 3.67 (2H, br s, C*H_Carb_*) ppm; ^11^B NMR (128 MHz, CDCl_3_): δ −2.5 (2B, d, J = 149 Hz), −4.7 (1B, s, *B*-C), −8.5 (1B, d, J = 149 Hz), −13.0 (3B, d, J = 156 Hz), −13.6 (3B, d, J = 159 Hz) ppm; ^13^C NMR (100 MHz, CDCl_3_): δ 161.1 (*C_Ar_*-O), 134.7 (*C_Ar_*H), 122.3 (*C_Ar_*-B), 114.0 (*C_Ar_*H), 56.8 (*C_Carb_*H), 56.1 (O*C*H_3_), 55.4 (*C_Carb_*H) ppm. MS (DUIS): *m*/*z* for C_9_H_18_B_10_O: calcd. 249.2 [M−H]^−^, obsd. 249.3 [M−H]^−^. Crystallographic data (CCDC number 2118711): C_9_H_18_B_10_O are orthorhombic, space group *P*2_1_2_1_2_1_: *a* = 9.5602(2) Å, *b* = 11.2642(3) Å, *c* = 12.9598(3) Å, *V* = 1395.62(6) Å^3^, *Z* = 4, *M* = 250.33, *d*_cryst_ = 1.191 g⋅cm^−3^. *wR*2 = 0.0857 calculated on *F*^2^*_hkl_* for all 3377 independent reflections with 2*θ* < 56.0°, (*GOF* = 1.048, *R* = 0.0308 calculated on *F_hkl_* for 3200 reflections with *I* > 2*σ*(*I*)).

3-(2′-Cyanophenyl)-*ortho*-carborane (**6**): 2-cyanophenyl bromide (455 mg, 2.50 mmol) was used; a mixture of chloroform and hexane (1:1, *v*/*v*) was used as the eluent for column chromatography; a pale-yellow crystalline solid was obtained (88 mg, yield 36%). ^1^H NMR (400 MHz, CDCl_3_): δ 8.14 (1H, d, J = 7.6 Hz, C*H_Ar_*), 7.69 (2H, m, C*H_Ar_*), 7.54 (1H, dd, J_1_ = 7.6 Hz, J_2_ = 7.5 Hz, C*H_Ar_*), 4.37 (2H, br s, C*H_Carb_*) ppm; ^11^B NMR (128 MHz, CDCl_3_): δ −2.4 (2B, d, J = 149 Hz), −7.0 (1B, s, *B*-C), −8.6 (1B, d, J = 153 Hz), −10.7 (1B, d, J = 152 Hz), −12.1 (2B, d, J = 161 Hz), −13.0 (2B, d, J = 150 Hz), −13.9 (1B, d, J = 176 Hz) ppm; ^13^C NMR (100 MHz, CDCl_3_): δ 138.3 (*C_Ar_*H), 133.8 (*C_Ar_*H), 132.8 (*C_Ar_*H), 130.0 (*C_Ar_*H), 119.7 (*C*N), 113.5 (*C_Ar_*-CN), 55.6 (*C_Carb_*H) ppm. MS (DUIS): *m*/*z* for C_9_H_15_B_10_N: calcd. 244.2 [M−H]^−^, obsd. 244.3 [M−H]^−^. Crystallographic data (CCDC number 2118710): C_9_H_15_B_10_N are monoclinic, space group *P*2_1_/*c*: *a* = 6.7263(3) Å, *b* = 19.8472(8) Å, *c* = 10.5592(4) Å, β = 105.7989(17)°, *V* = 1356.38(10) Å^3^, *Z* = 4, *M* = 245.32, *d*_cryst_ = 1.201 g⋅cm^−3^. *wR*2 = 0.1139 calculated on *F*^2^*_hkl_* for all 3247 independent reflections with 2*θ* < 55.9°, (*GOF* = 1.045, *R* = 0.0406 calculated on *F_hkl_* for 2535 reflections with *I* > 2*σ*(*I*)).

3-(4′-Cyanophenyl)-*ortho*-carborane (**7**): 4-cyanophenyl bromide (455 mg, 2.50 mmol) was used; a mixture of chloroform and hexane (3:1, *v*/*v*) was used as the eluent for column chromatography; a pale-yellow crystalline solid was obtained (221 mg, yield 90%). ^1^H NMR (400 MHz, CDCl_3_): δ 7.69 (2H, m, C*H_Ar_*), 7.63 (2H, m, C*H_Ar_*), 3.74 (2H, br s, C*H_Carb_*) ppm; ^11^B NMR (128 MHz, CDCl_3_): δ −2.0 (2B, d, J = 149 Hz), −6.3 (1B, s, *B*-C), −8.5 (1B, d, J = 151 Hz), −11.4 (1B, d, J = 148 Hz), −13.0 (5B, d, J = 164 Hz) ppm; ^13^C NMR (100 MHz, CDCl_3_): δ 133.8 (*C_Ar_*H), 131.7 (*C_Ar_*H), 118.5 (*C*N), 113.6 (*C_Ar_*-CN), 56.6 (*C_Carb_*H) ppm. MS (DUIS): *m*/*z* for C_9_H_15_B_10_N: calcd. 244.2 [M−H]^−^, obsd. 244.3 [M−H]^−^. Crystallographic data (CCDC number 2118709): C_9_H_15_B_10_N are monoclinic, space group *P*2_1_/*n*: *a* = 7.1000(10) Å, *b* = 17.782(2) Å, *c* = 10.7239(14) Å, β = 93.146(5)°, *V* = 1351.9(3) Å^3^, *Z* = 4, *M* = 245.32, *d*_cryst_ = 1.205 g⋅cm^−3^. *wR*2 = 0.2020 calculated on *F*^2^*_hkl_* for all 2949 independent reflections with 2*θθ* < 54.2°, (*GOF* = 1.323, *R* = 0.0792 calculated on *F_hkl_* for 2335 reflections with *I* > 2*σ*(*I*)).

3-(2′-Ethoxycarbonylphenyl)-*ortho*-carborane (**8**): 2-ethoxycarbonyl bromide (398 μL, 573 mg, 2.50 mmol) was used; a mixture of chloroform and hexane (2:1, *v*/*v*) was used as the eluent for column chromatography; a colorless oil was obtained (100 mg, yield 34%). ^1^H NMR (400 MHz, CDCl_3_): δ 8.24 (1H, d, J = 7.5 Hz, C*H_Ar_*), 7.81 (1H, dd, J^3^ = 7.8 Hz, J^4^ = 1.3 Hz, C*H_Ar_*), 7.55 (1H, ddd, J^3^_1_ = 7.5 Hz, J^3^_2_ = 7.5 Hz, J^4^ = 1.3 Hz, C*H_Ar_*), 7.46 (1H, ddd, J^3^_1_ = 7.8 Hz, J^3^_2_ = 7.5 Hz, J^4^ = 1.3 Hz, C*H_Ar_*), 4.38 (2H, br s, C*H_Carb_*), 4.33 (2H, q, J = 7.1 Hz, OC*H_2_*CH_3_), 1.40 (3H, t, J = 7.1 Hz, OC*H_2_*CH_3_) ppm; ^11^B NMR (128 MHz, CDCl_3_): δ −3.3 (2B, d, J = 147 Hz), −6.1 1B, (s, *B*-C), −8.2 (1B, d, J = 148 Hz), −11.2 (1B, d, J = 134 Hz), −11.9 (2B, d, J = 163 Hz), −13.9 (3B, d, J = 169 Hz) ppm; ^13^C NMR (100 MHz, CDCl_3_): δ 170.0 (*C*O), 139.8 (*C_Ar_*H), 134.5 (*C_Ar_*-CO), 131.3 (*C_Ar_*H), 130.3 (*C_Ar_*H), 129.2 (*C_Ar_*H), 62.0 (O*C*H_2_CH_3_), 57.0 (*C_Carb_*H), 14.3 (OCH_2_*C*H_3_) ppm. MS (DUIS): *m*/*z* for C_11_H_20_B_10_O_2_: calcd. 245.2 [M−H−EtOH]^−^, obsd. 245.3 [M−H−EtOH]^−^.

3-(3′-Ethoxycarbonylphenyl)-*ortho*-carborane (**9**): 3-ethoxycarbonyl bromide (401 μL, 573 mg, 2.50 mmol) was used; a mixture of chloroform and hexane (2:1, *v*/*v*) was used as the eluent for column chromatography; a pale-yellow crystalline solid was obtained (161 mg, yield 55%). ^1^H NMR (400 MHz, CDCl_3_): δ 8.17 (1H, s, C*H_Ar_*), 8.08 (1H, d, J = 7.8 Hz, C*H_Ar_*), 7.85 (1H, d, J = 7.6 Hz, C*H_Ar_*), 7.46 (1H, dd, J_1_ = 7.8 Hz, J_2_ = 7.6 Hz, C*H_Ar_*), 4.39 (2H, q, J = 7.1 Hz, OC*H_2_*CH_3_), 3.77 (2H, br s, C*H_Carb_*), 1.40 (3H, t, J = 7.1 Hz, OC*H_2_*CH_3_) ppm; ^11^B NMR (128 MHz, CDCl_3_): δ −2.2 (2B, d, J = 149 Hz), −5.5 (1B, s, *B*-C), −8.6 (1B, d, J = 150 Hz), −12.0 (1B, d, J = 134 Hz), −12.9 (2B, d, J = 154 Hz), −13.4 (3B, d, J = 168 Hz) ppm; ^13^C NMR (100 MHz, CDCl_3_): δ 166.5 (*C*O), 138.1 (*C_Ar_*H), 133.5 (*C_Ar_*H), 130.9 (*C_Ar_*H), 130.5 (*C_Ar_*-CO), 128.6 (*C_Ar_*H), 61.4 (O*C*H_2_CH_3_), 56.8 (*C_Carb_*H), 14.5 (OCH_2_*C*H_3_) ppm. MS (DUIS): *m*/*z* for C_11_H_20_B_10_O_2_: calcd. 291.2 [M−H]^−^, obsd. 291.3 [M−H]^−^. Crystallographic data (CCDC number 2124597): C_11_H_20_B_10_O_2_ are monoclinic, space group *P2*_1_*/n*: *a* = 7.0480(7) Å, *b* = 10.3342(10) Å, *c* = 21.972(2) Å, *β* = 90.515(5)°, *V* = 1600.3(3) Å^3^, *Z* = 4, *M* = 292.37, *d*_cryst_ = 1.214 g·cm^−3^. *wR*_2_ = 0.1601 calculated on *F*^2^*_hkl_* for all 3144 independent reflections with 2*θ* < 52.0°, (*GOF* = 1.149, *R* = 0.0512 calculated on *F_hkl_* for 2555 reflections with *I* > 2*σ*(*I*)).

3-(4′-Ethoxycarbonylphenyl)-*ortho*-carborane (**10**): 4-ethoxycarbonyl bromide (408 μL, 573 mg, 2.50 mmol) was used; a mixture of chloroform and hexane (1:5, *v*/*v*) and diethyl ether were used as the eluent for column chromatography; a pale-yellow crystalline solid was obtained (126 mg, yield 43%). ^1^H NMR (400 MHz, CDCl_3_): δ 8.01 (2H, d, J = 8.0 Hz, C*H_Ar_*), 7.67 (2H, d, J = 8.0 Hz, C*H_Ar_*), 4.39 (2H, q, J = 7.1 Hz, OC*H_2_*CH_3_), 3.75 (2H, br s, C*H_Carb_*), 1.40 (3H, t, J = 7.1 Hz, OC*H_2_*CH_3_) ppm; ^11^B NMR (128 MHz, CDCl_3_): δ −2.2 (2B, d, J = 150 Hz), −5.7 (1B, s, *B*-C), −8.5 (1B, d, J = 149 Hz), −11.8 (1B, d, J = 146 Hz), −12.9 (5B, d, J = 157 Hz) ppm; ^13^C NMR (100 MHz, CDCl_3_): δ 166.4 (*C*O), 149.9 (*C_Ar_*-B), 133.2 (*C_Ar_*H), 132.9 (*C_Ar_*-CO), 129.2 (*C_Ar_*H), 61.3 (O*C*H_2_CH_3_), 56.7 (*C_Carb_*H), 14.5 (OCH_2_*C*H_3_) ppm. MS (DUIS): *m*/*z* for C_11_H_20_B_10_O_2_: calcd. 291.2 [M−H]^−^, obsd. 291.3 [M−H]^−^. Crystallographic data (CCDC number 2124598): C_11_H_20_B_10_O_2_ are monoclinic, space group *P2*_1_/*n*: *a* = 16.8494(13) Å, *b* = 7.0272(6) Å, *c* = 28.549(2) Å, *β* = 105.387(2)°, *V* = 3259.2(5) Å^3^, *Z* = 8, *M* = 292.37, *d*_cryst_ = 1.192 g·cm^−3^. *wR*_2_ = 0.1266 calculated on *F*^2^*_hkl_* for all 7113 independent reflections with 2*θ* < 54.2°, (*GOF* = 1.006, *R* = 0.0503 calculated on *F_hkl_* for 4786 reflections with *I* > 2*σ*(*I*)).

## Figures and Tables

**Table 1:** Some selected angles in 3-aryl-*ortho*-carboranes **3**, **5**–**7**, **9** and **10**.

Compound	Selected Angles, °			
	C(3)-B(3)-B(10)	C(3)-B(3)-C(1)	C(3)-B(3)-C(2)	C(3)-B(3)-B(8)
**3**	173.72(9)	121.84(9)	122.88(9)	128.70(9)
**5**	171.88(12)	120.71(12)	120.91(11)	130.56(11)
**6**	171.08(10)	120.91(9)	119.28(9)	130.81(9)
**7**	168.2(3)	117.2(3)	117.5(2)	133.5(3)
**9**	170.0(2)	118.4(2)	120.0(2)	132.0(2)
**10A**	173.3(2)	122.2(2)	121.4(2)	128.7(2)
**10A′**	172.7(2)	120.8(2)	122.1(2)	129.3(2)

## Data Availability

The data presented in this study are available from the authors.

## Supplementary Materials

The following are available online. Figure S1: H-bonded chain in the crystal structure of compound **6**. Figure S2: Fragment of the crystal packing of compound **10**. Reagents and equipment used for the synthesis and characterization of 3-aryl-*ortho*-carboranes, NMR and mass-spectra of compounds **2**–**10**. References [54,81,82,83,84,85] are cited in the supplementary materials.

## Sample Availability

Samples of the compounds **2**–**10** are available from the authors.
