# Supplementary material for: Synthesis of 3-Aryl-ortho-carboranes with Sensitive Functional Groups
Source: Molecules. 2021 Dec 1;26(23):7297. doi: 10.3390/molecules26237297 (PMC8659134; doi:10.3390/molecules26237297)

SUPPORTING INFORMATION

Synthesis of 3-Aryl-*ortho*-carboranes with Sensitive Functional Groups

Sergey A. Anufriev, Akim V. Shmal'ko, Kyrill Yu. Suponitsky and Igor B. Sivaev *

A.N. Nesmeyanov Institute of Organoelement Compounds, Russian Academy of Sciences, 28 Vavilov Str., 119991, Moscow, Russia; trueman476@mail.ru (S.A.A.); akimbend@yandex.ru (A.V.S.); kirshik@yahoo.com (K.Y.S.); sivaev@ineos.ac.ru (I.B.S.)

* Correspondence: sivaev@ineos.ac.ru; Tel.: +7-916-5902025

Materials and Methods 2

Figure S1. H-bonded chain in the crystal structure of compound **6**. 3

Figure S2. Fragment of the crystal packing of compound **10**. 3

NMR and mass spectra of compound **2** 4

NMR and mass spectra of compound **3** 10

NMR and mass spectra of compound **4** 18

NMR and mass spectra of compound **5** 25

NMR and mass spectra of compound **6** 32

NMR and mass spectra of compound **7** 40

NMR and mass spectra of compound **8** 47

NMR and mass spectra of compound **9** 56

NMR and mass spectra of compound **10** 64

Materials and Methods

3-Iodo-*ortho*-carborane (**1**) [54] and bis(triphenylphosphine)palladium dichloride [81] were prepared according to the literature procedure. Anhydrous cobalt dibromide was prepared by heating cobalt bromide hexahydrate at 160 °C under vacuum for 3 h and stored under argon atmosphere. Acetonitrile was dried using standard procedures [82]. All other chemical reagents were purchased from Sigma Aldrich, Acros Organics and ABCR and used as received. All reactions were carried out under argon atmosphere. The reaction progress was monitored by thin layer chromatography (Merck F254 silica gel on aluminum plates) and visualized using 0.5% $PdCl_2$ in 1% HCl in aq. MeOH (1:10). Acros Organics silica gel (0.060–0.200 mm) was used for column chromatography. The NMR spectra at 400 MHz (^{1}H), 128 MHz (^{11}B) and 100 MHz (^{13}C) were recorded with Varian Inova 400 spectrometer. The residual signal of the NMR solvent relative to Me_4Si was taken as the internal reference for ^{1}H and ^{13}C NMR spectra. ^{11}B NMR spectra were referenced using $BF_3{\cdot}Et_2O$ as external standard. Mass spectra (MS) were measured using Shimadzu LCMS-2020 instrument with DUIS ionization (ESI—Electrospray ionization and APCI—Atmospheric pressure chemical ionization). The measurements were done in a positive and negative ion modes with mass range from m/z 50 to *m*/*z* 2000. Isotope distribution were calculated using Isotope Distribution Calculator and Mass Spec Plotter [83].

Single crystal X-ray diffraction experiments for compounds **5**-**7** were carried out using SMART APEX2 CCD diffractometer (λ(Mo-Kα) = 0.71073 Å, graphite monochromator, *ω*-scans) at 120 K. Collected data were processed by the SAINT and SADABS programs incorporated into the APEX2 program package [84]. The structures were solved by the direct methods and refined by the full-matrix least-squares procedure against F^2 in anisotropic approximation. The refinement was carried out with the SHELXTL program [85]. The CCDC numbers (2118711, 2118710, and 2118709 for **5**, **6**, and **7**, respectively) contain the supplementary crystallographic data for this paper. These data can be obtained free of charge via www.ccdc.cam.ac.uk/data_request/cif.

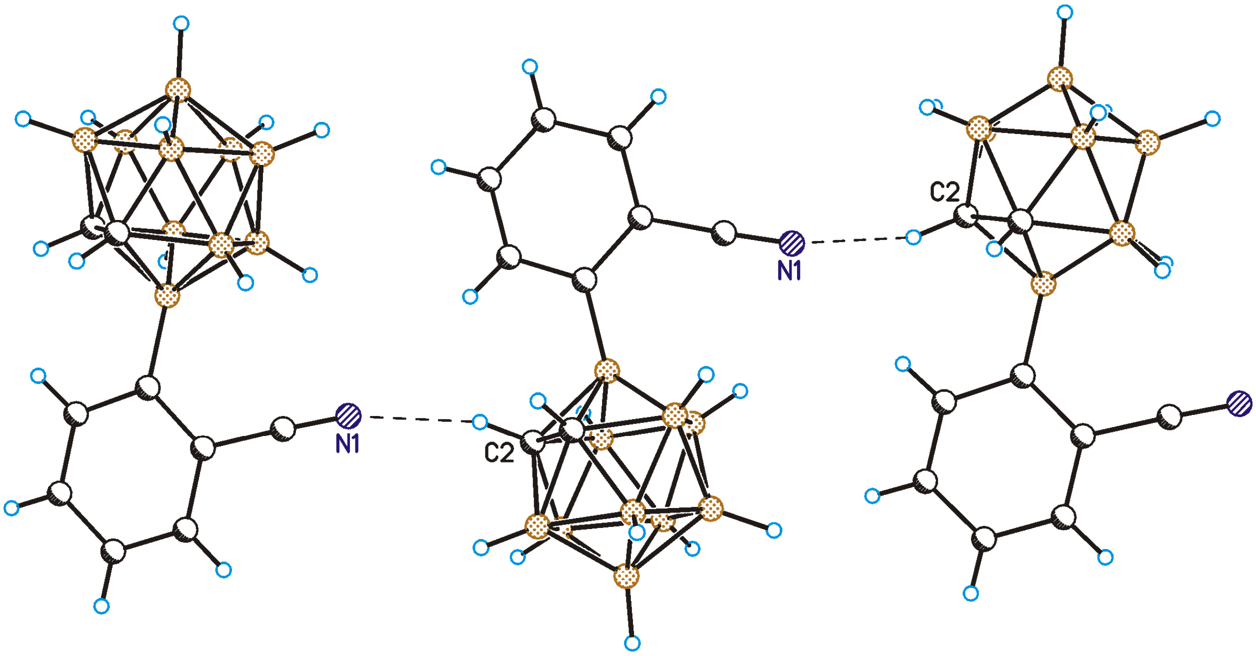

Figure S1. H-bonded chain in the crystal structure of compound **6**.

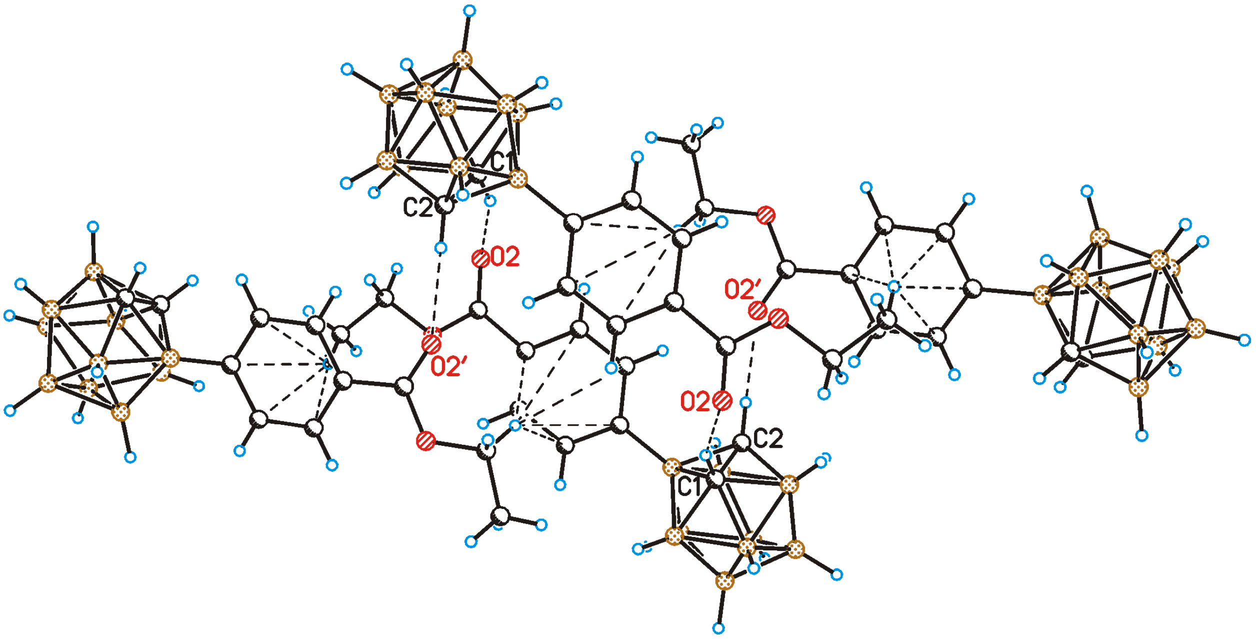

Figure S2. Fragment of the crystal packing of compound **10**. Tetramer formed by two molecules A and two molecules A' by means of H…π and C_{carb}-H…O intermolecular hydrogen bonds.

1H, compound 2

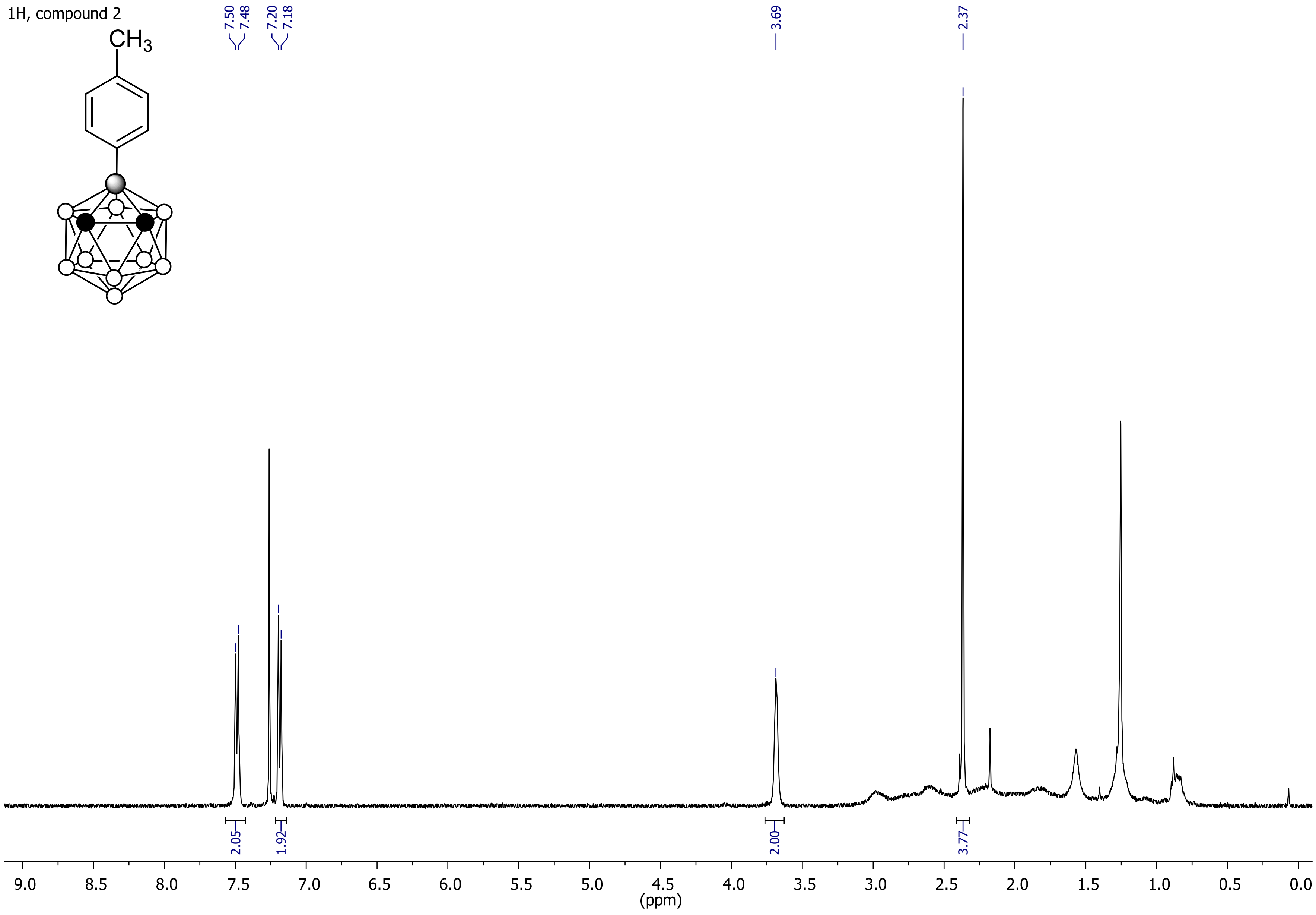

11B{1H}, compound 2

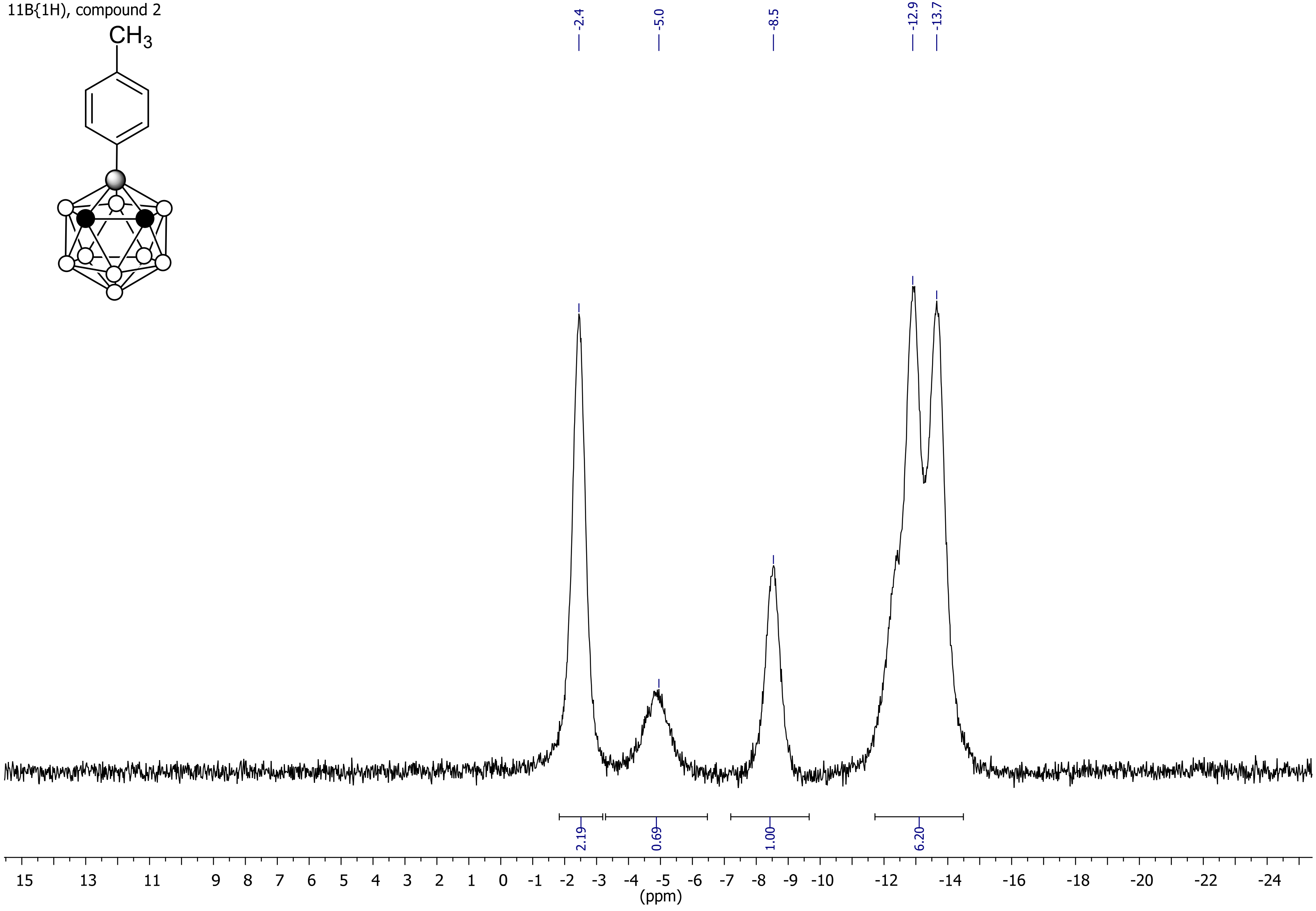

11B, compound 2

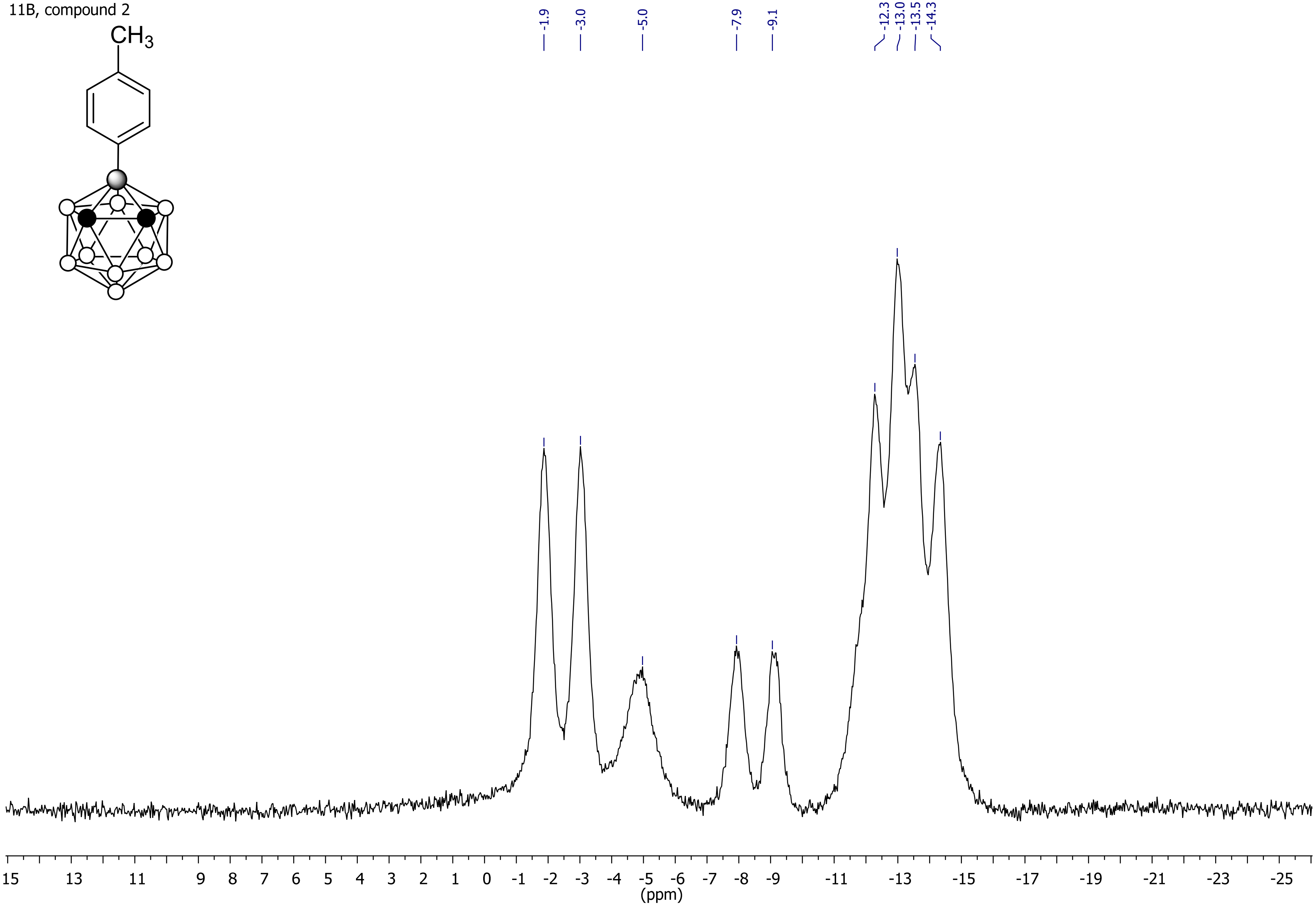

13C{1H}, compound 2

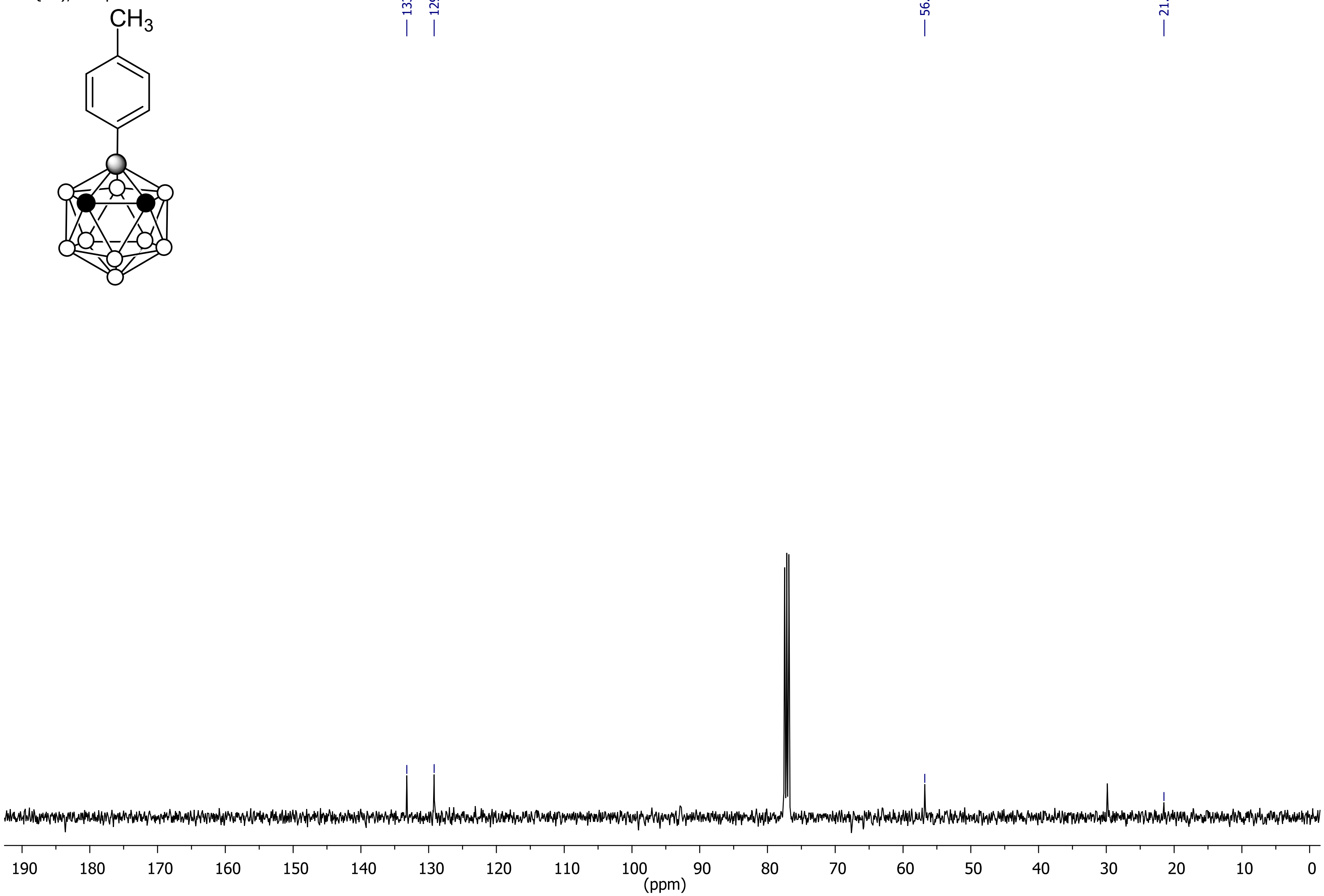

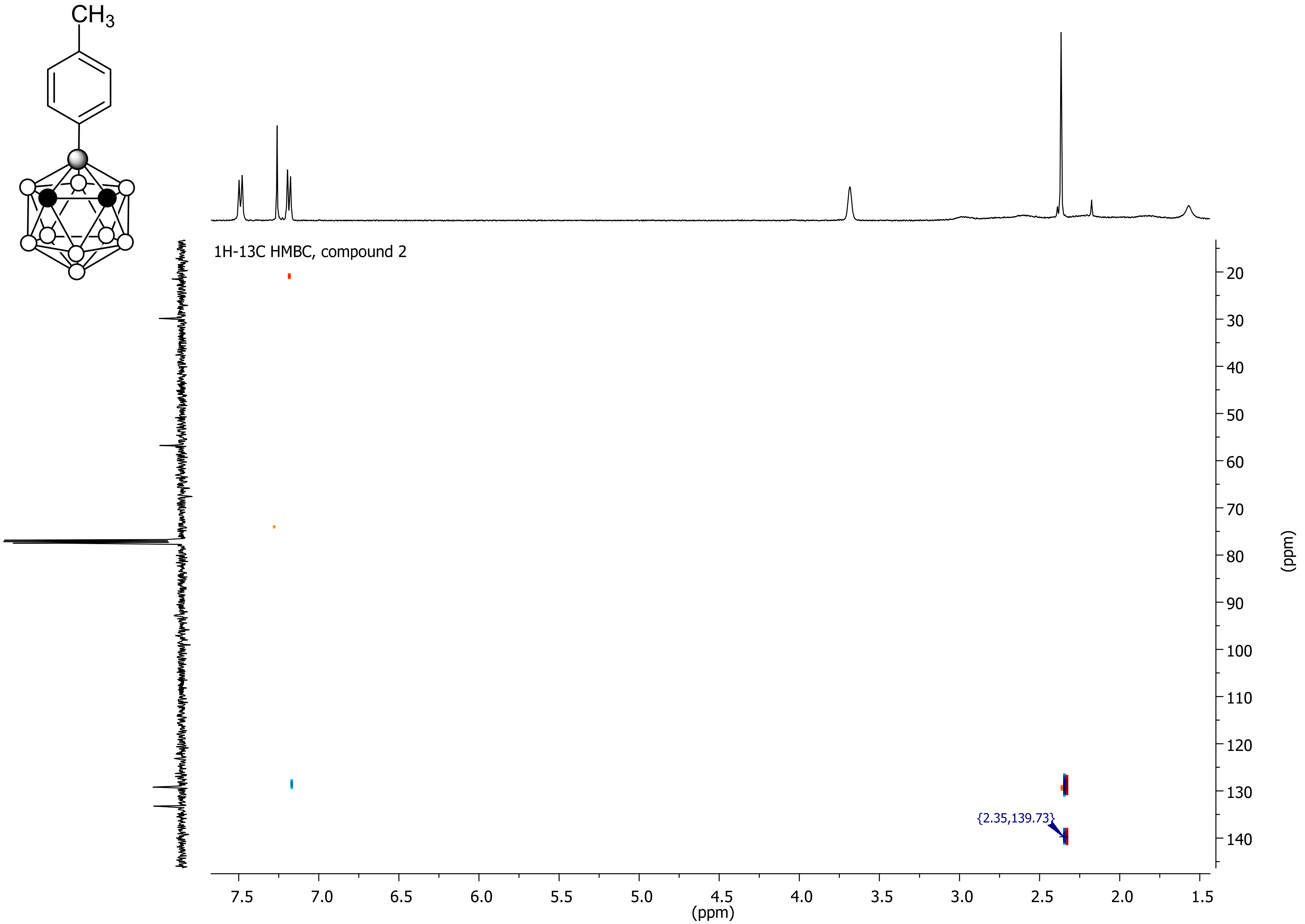

CH3
1H-13C HMBC, compound 2
{2.35,139.73}
20
30
40
50
60
70
80
90
100
110
120
130
140
(ppm)
7.5
7.0
6.5
6.0
5.5
5.0
4.5
4.0
3.5
3.0
2.5
2.0
1.5
(ppm)

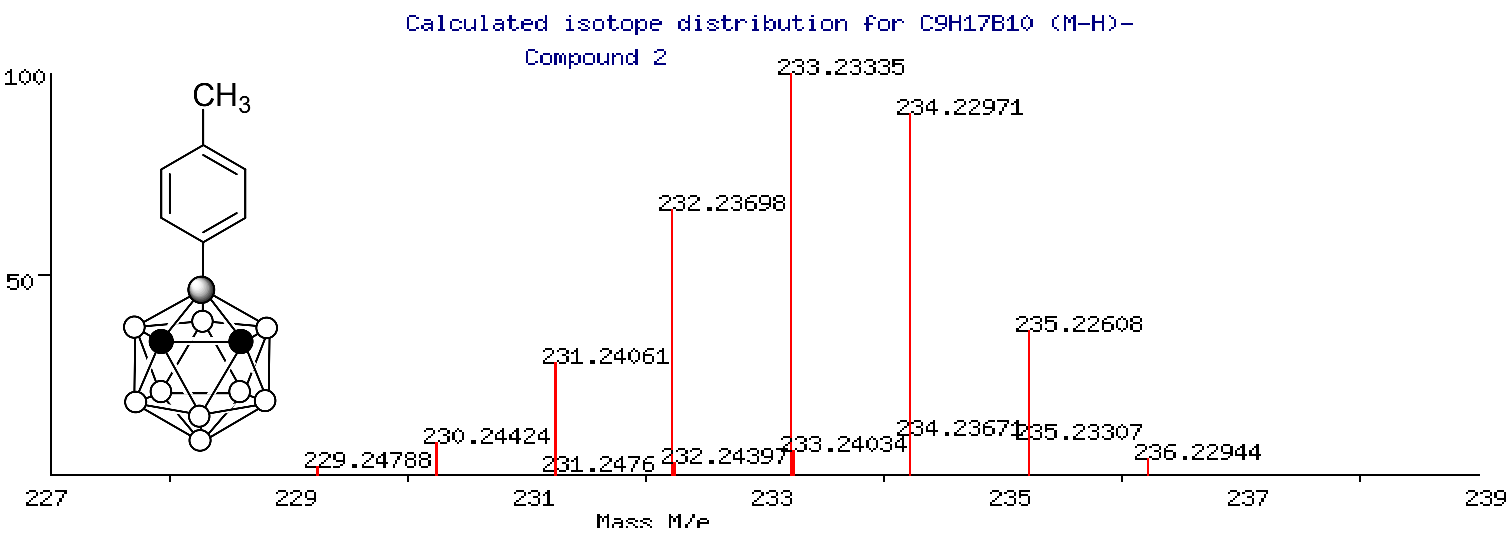
Calculated isotope distribution for C9H17B10 (M-H)-
Compound 2
CH3
100
50
233.23335
234.22971
232.23698
235.22608
231.24061
230.24424
229.24788
231.2476
232.24397
233.24034
234.23671
235.23307
236.22944
227
229
231
233
235
237
239
Mass M/e

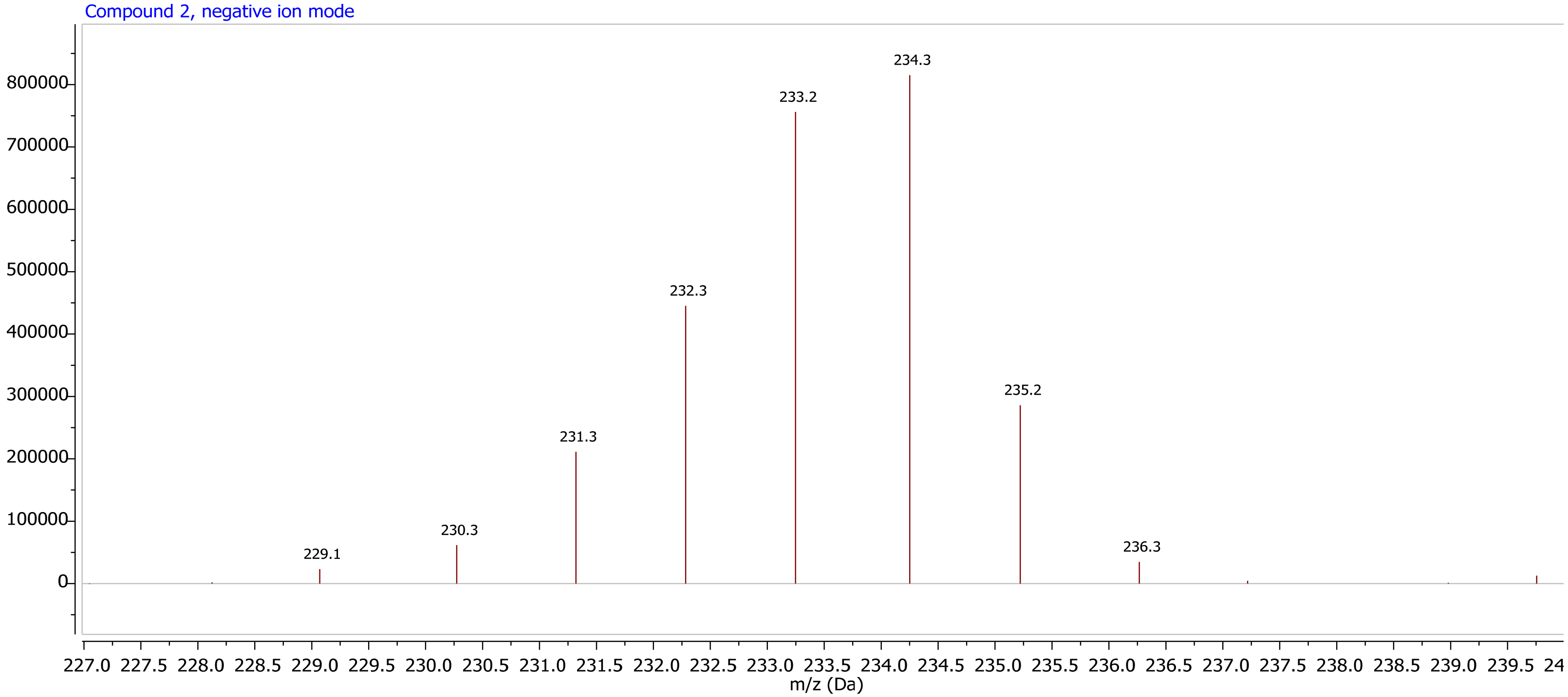
Compound 2, negative ion mode
800000
700000
600000
500000
400000
300000
200000
100000
0
229.1
230.3
231.3
232.3
233.2
234.3
235.2
236.3
227.0 227.5 228.0 228.5 229.0 229.5 230.0 230.5 231.0 231.5 232.0 232.5 233.0 233.5 234.0 234.5 235.0 235.5 236.0 236.5 237.0 237.5 238.0 238.5 239.0 239.5 24
m/z (Da)

1H, compound 3

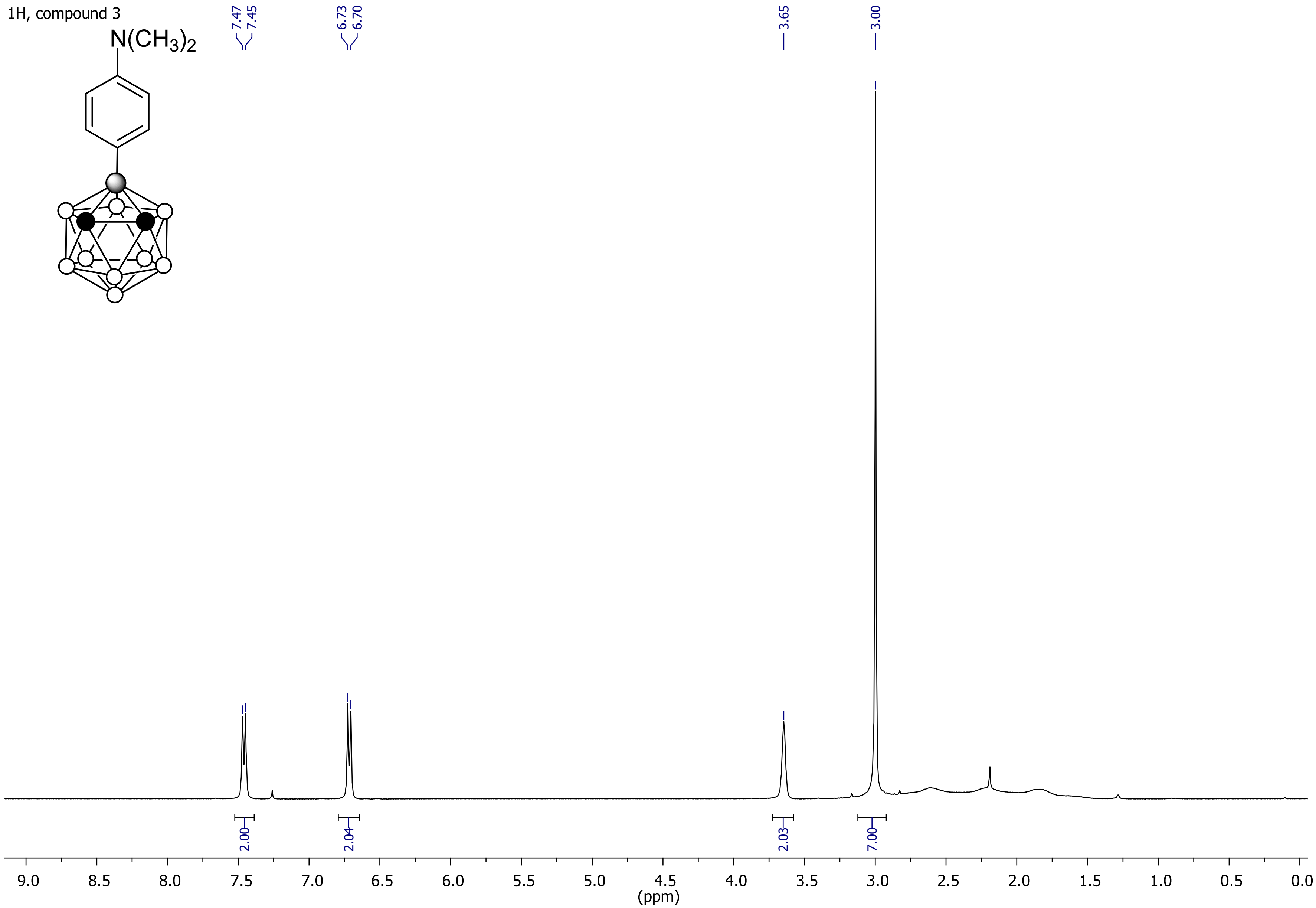

11B{1H}, compound 3

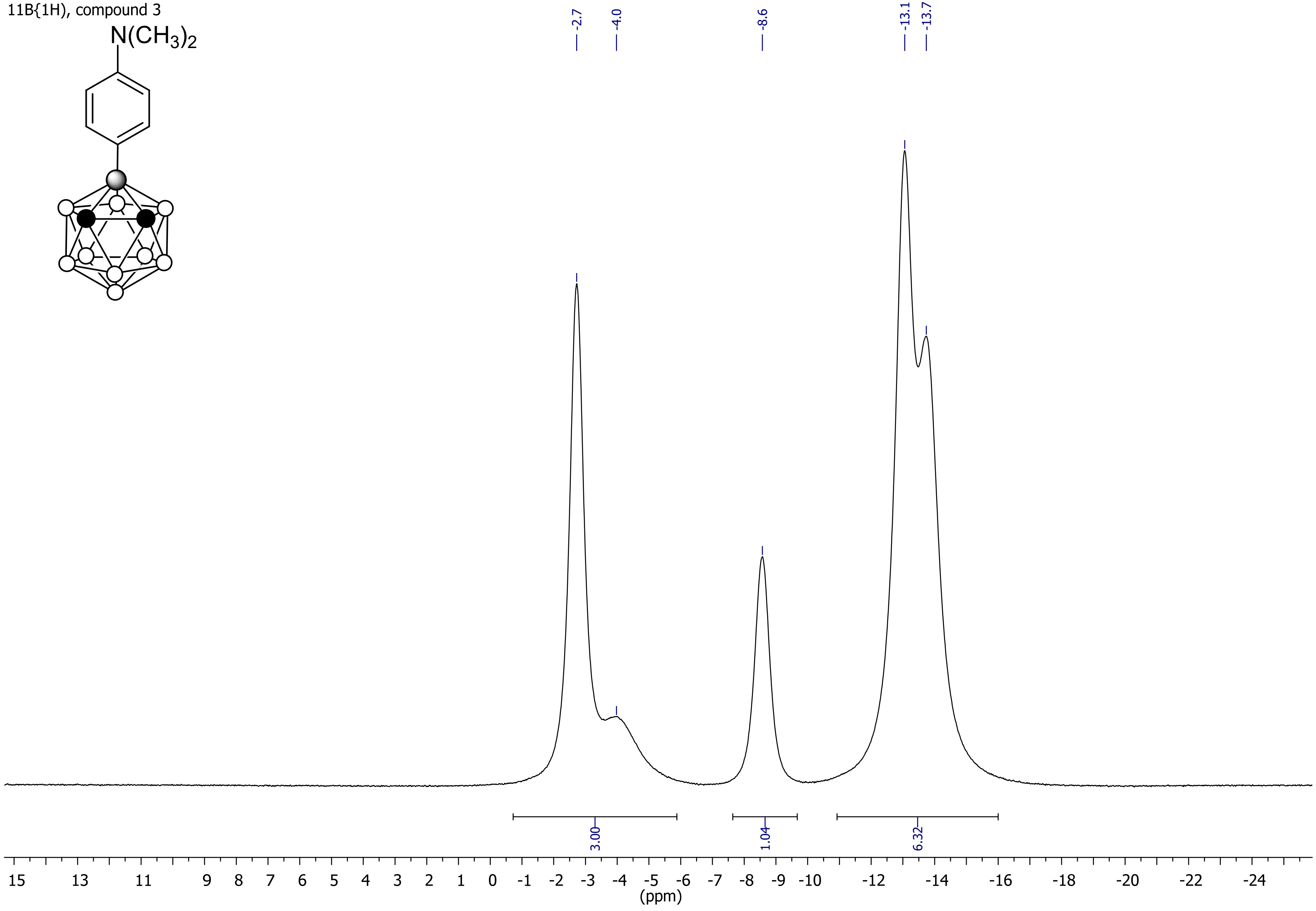

11B, compound 3

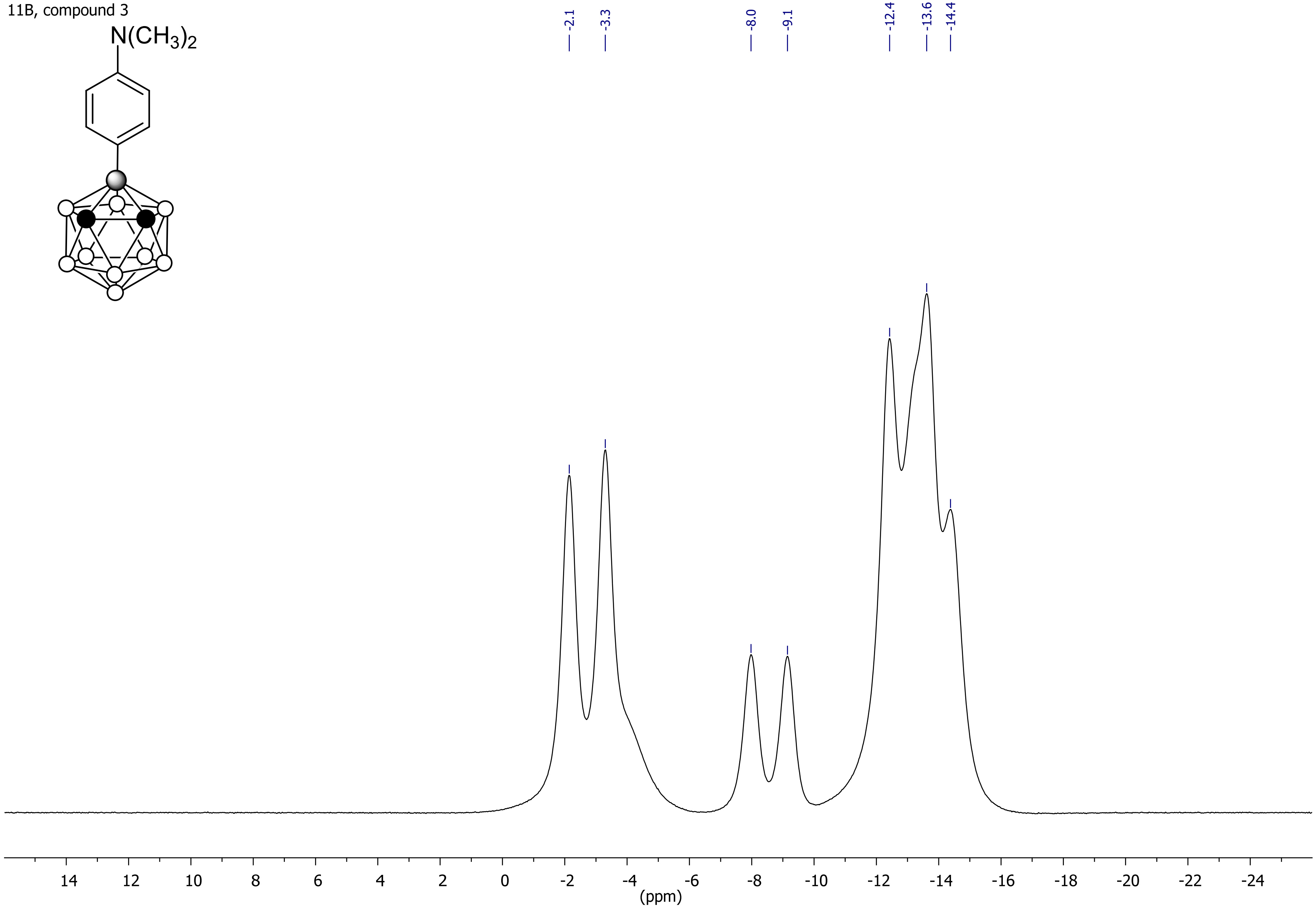

13C{1H}, compound 3

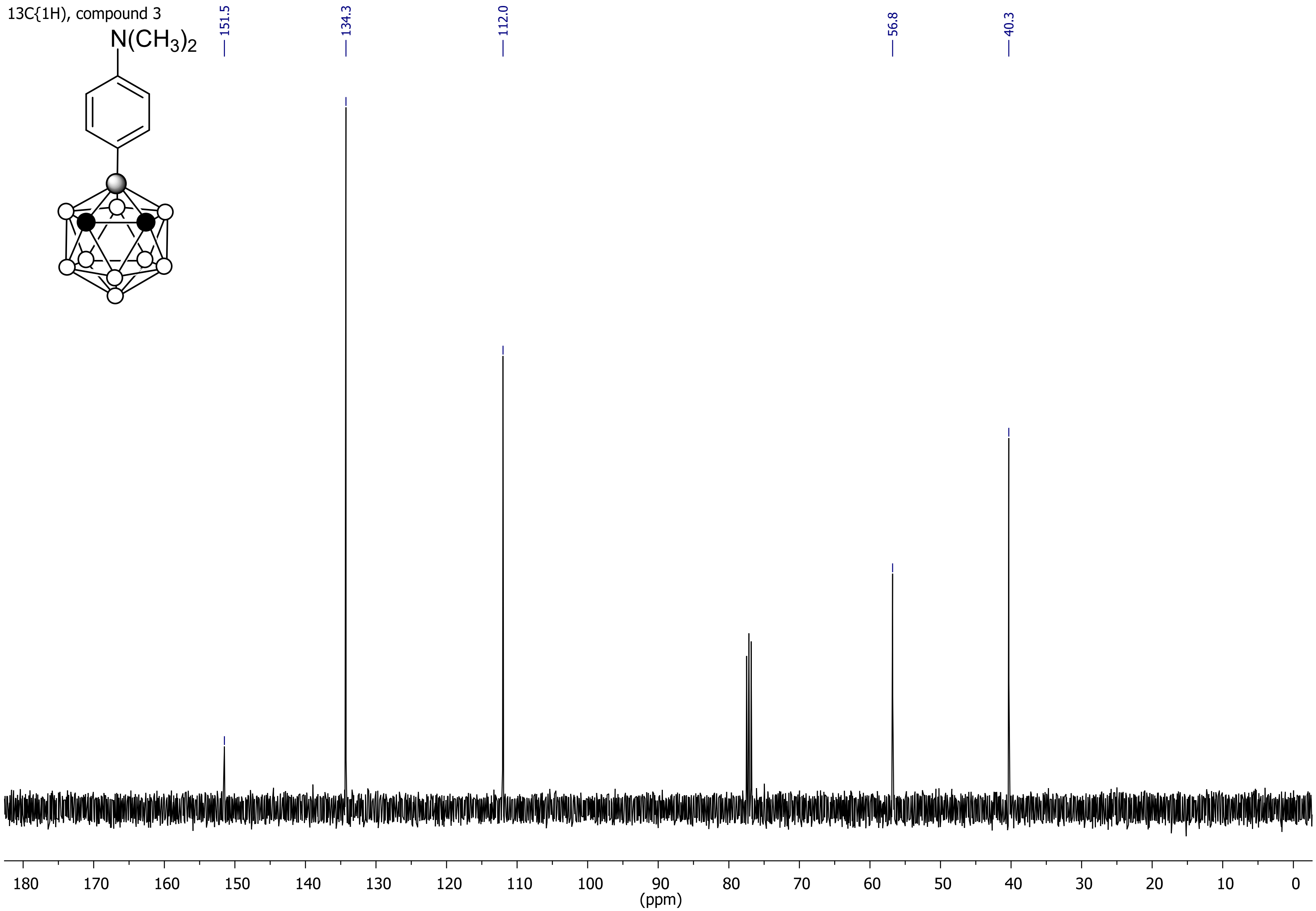

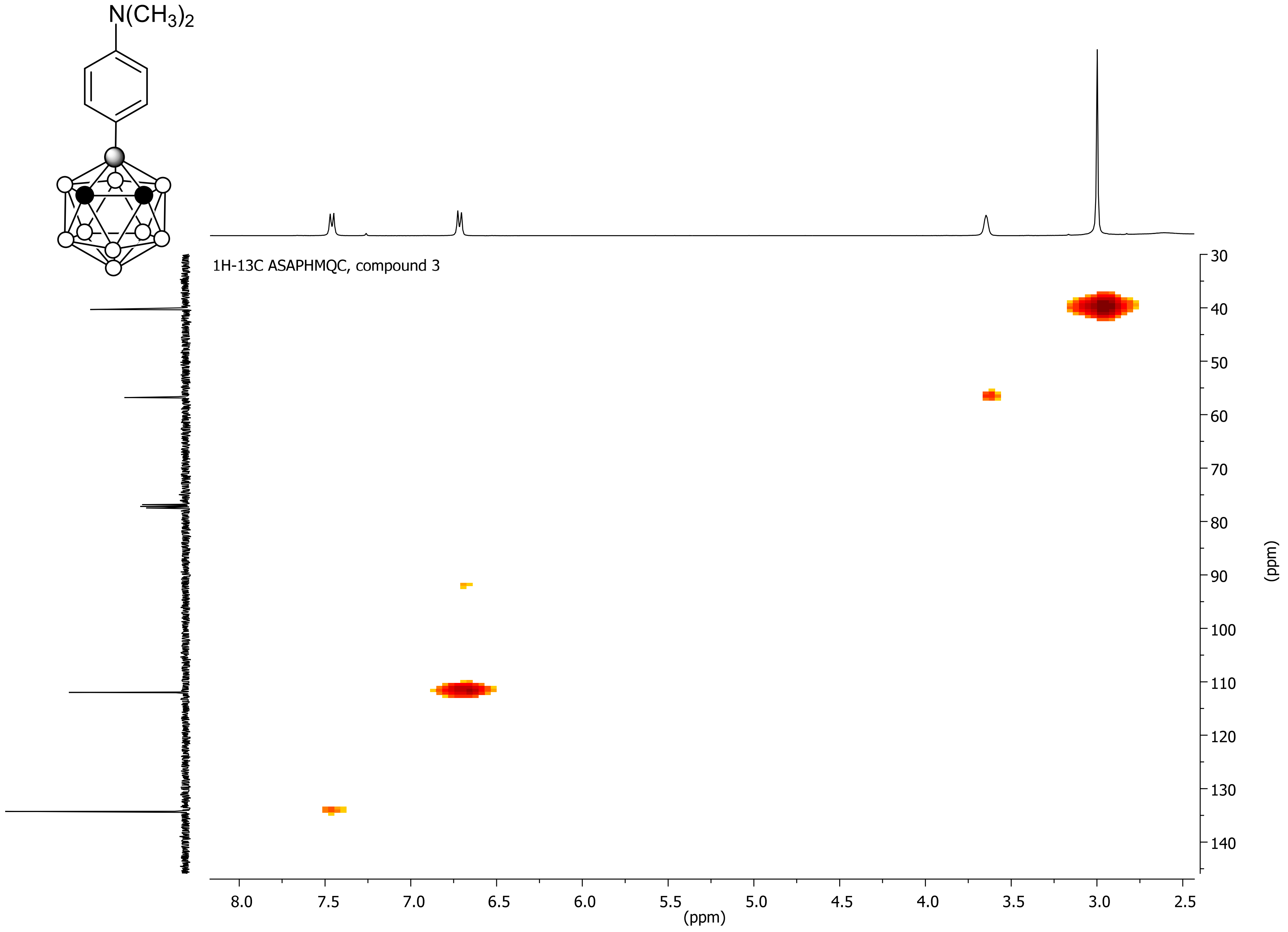
N(CH3)2
1H-13C ASAPHMQC, compound 3
30
40
50
60
70
80
90
100
110
120
130
140
(ppm)
8.0
7.5
7.0
6.5
6.0
5.5
5.0
4.5
4.0
3.5
3.0
2.5
(ppm)

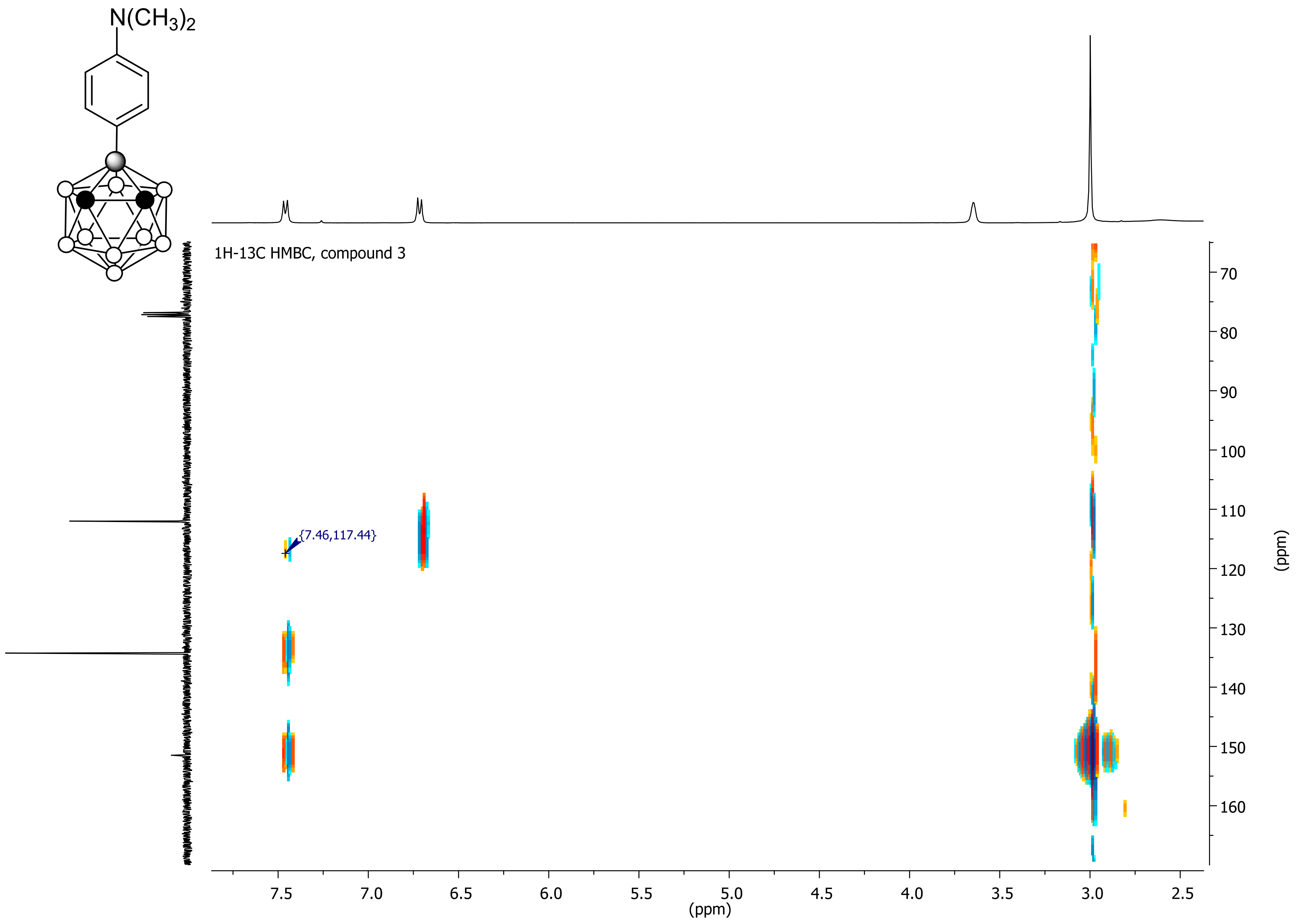

N(CH3)2
1H-13C HMBC, compound 3
{7.46,117.44}
70
80
90
100
110
120
130
140
150
160
(ppm)
7.5
7.0
6.5
6.0
5.5
5.0
4.5
4.0
3.5
3.0
2.5
(ppm)

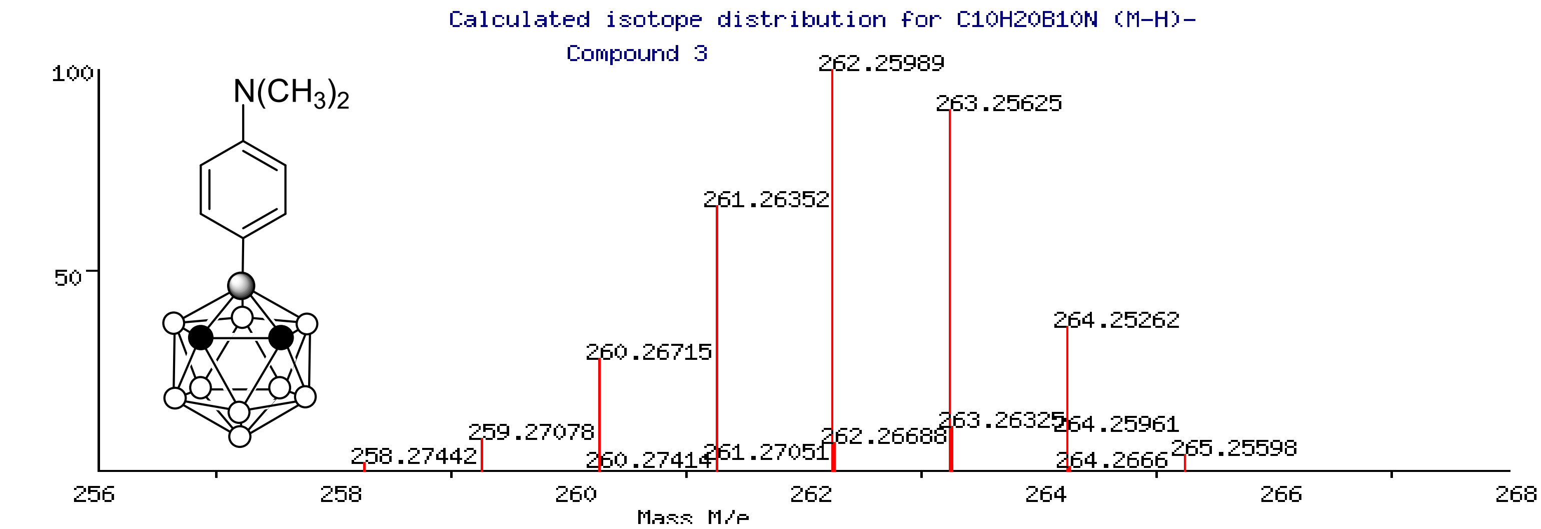
Calculated isotope distribution for C10H20B10N (M-H)-
Compound 3
N(CH3)2
100
50
262.25989
263.25625
261.26352
264.25262
260.26715
259.27078
258.27442
260.27414
261.27051
262.26688
263.26325
264.25961
264.2666
265.25598
256
258
260
262
264
266
268
Mass M/e

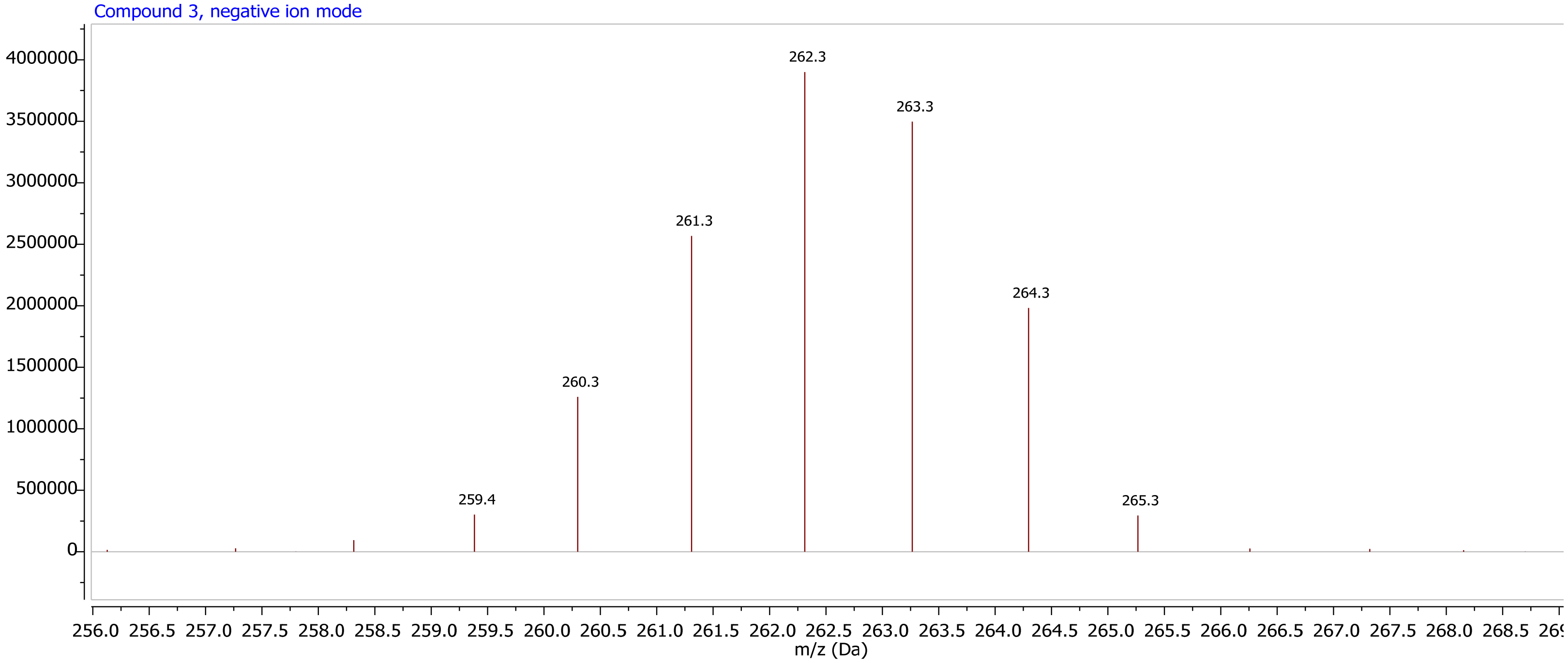
Compound 3, negative ion mode
4000000
3500000
3000000
2500000
2000000
1500000
1000000
500000
0
262.3
263.3
261.3
264.3
260.3
259.4
265.3
256.0 256.5 257.0 257.5 258.0 258.5 259.0 259.5 260.0 260.5 261.0 261.5 262.0 262.5 263.0 263.5 264.0 264.5 265.0 265.5 266.0 266.5 267.0 267.5 268.0 268.5
m/z (Da)

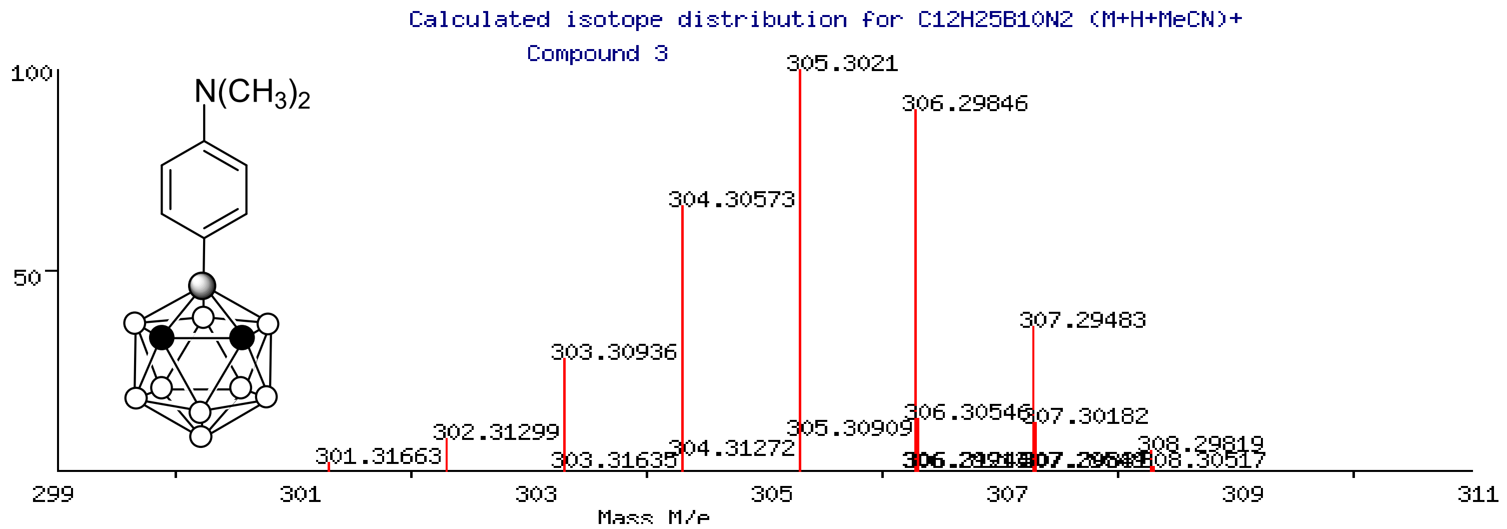
Calculated isotope distribution for C12H25B10N2 (M+H+MeCN)+
Compound 3
N(CH3)2
100
50
305.3021
306.29846
304.30573
307.29483
303.30936
302.31299
301.31663
303.31635
304.31272
305.30909
306.30546
307.30182
308.29819
308.30517
299
301
303
305
307
309
311
Mass M/e

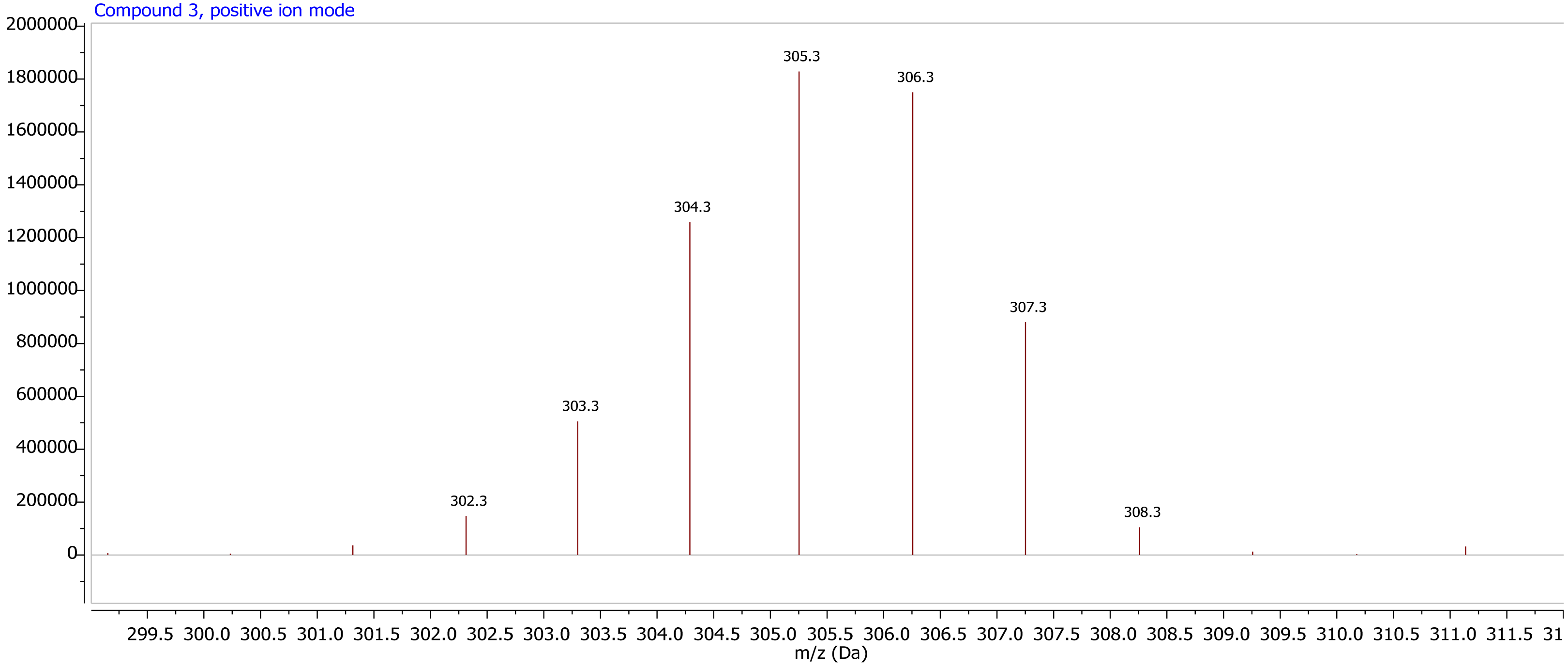
Compound 3, positive ion mode
2000000
1800000
1600000
1400000
1200000
1000000
800000
600000
400000
200000
0
305.3
306.3
304.3
307.3
303.3
302.3
308.3
299.5 300.0 300.5 301.0 301.5 302.0 302.5 303.0 303.5 304.0 304.5 305.0 305.5 306.0 306.5 307.0 307.5 308.0 308.5 309.0 309.5 310.0 310.5 311.0 311.5
m/z (Da)

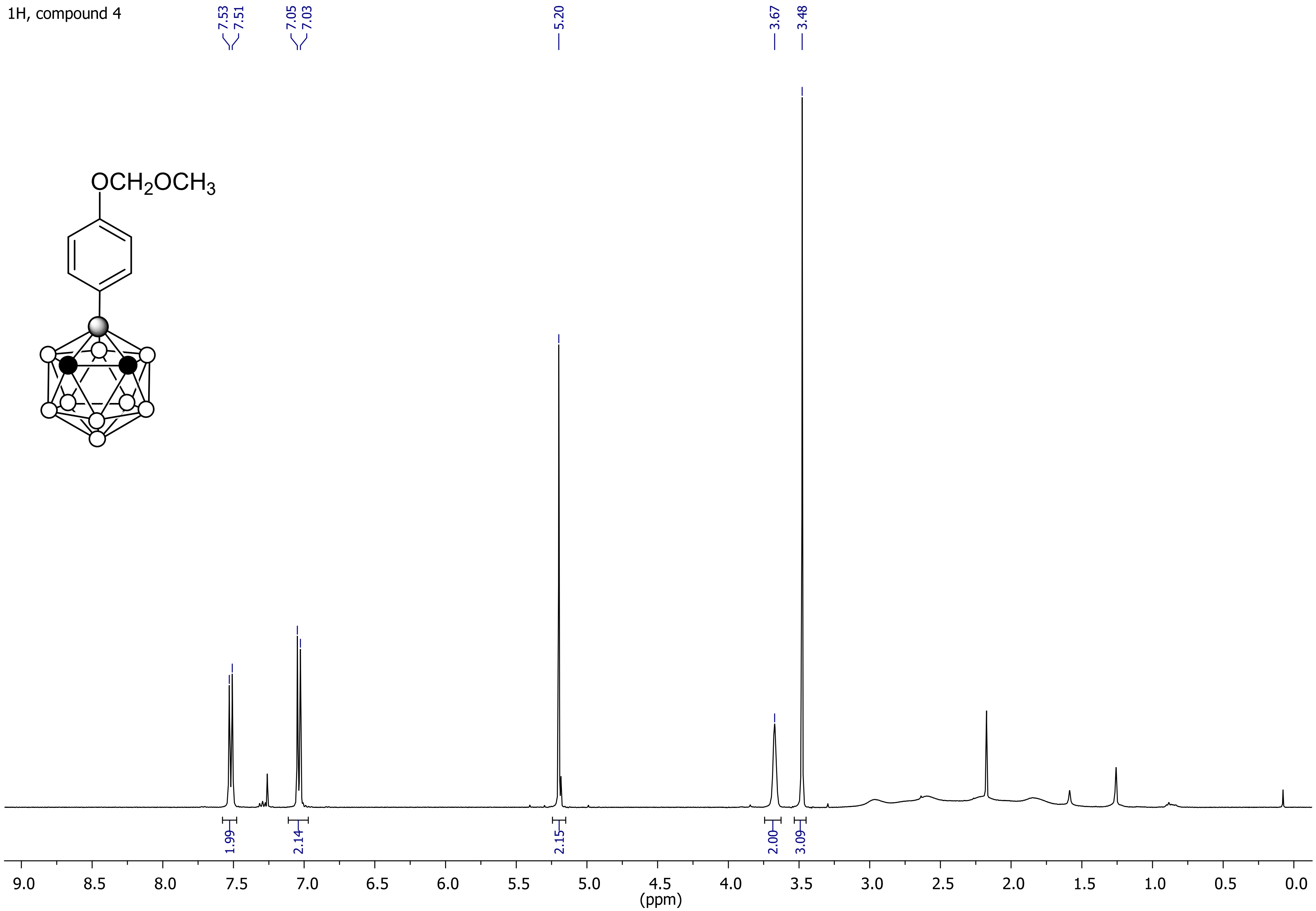
1H, compound 4
7.53
7.51
7.05
7.03
5.20
3.67
3.48
OCH2OCH3
1.99
2.14
2.15
2.00
3.09
9.0 8.5 8.0 7.5 7.0 6.5 6.0 5.5 5.0 4.5 4.0 3.5 3.0 2.5 2.0 1.5 1.0 0.5 0.0
(ppm)

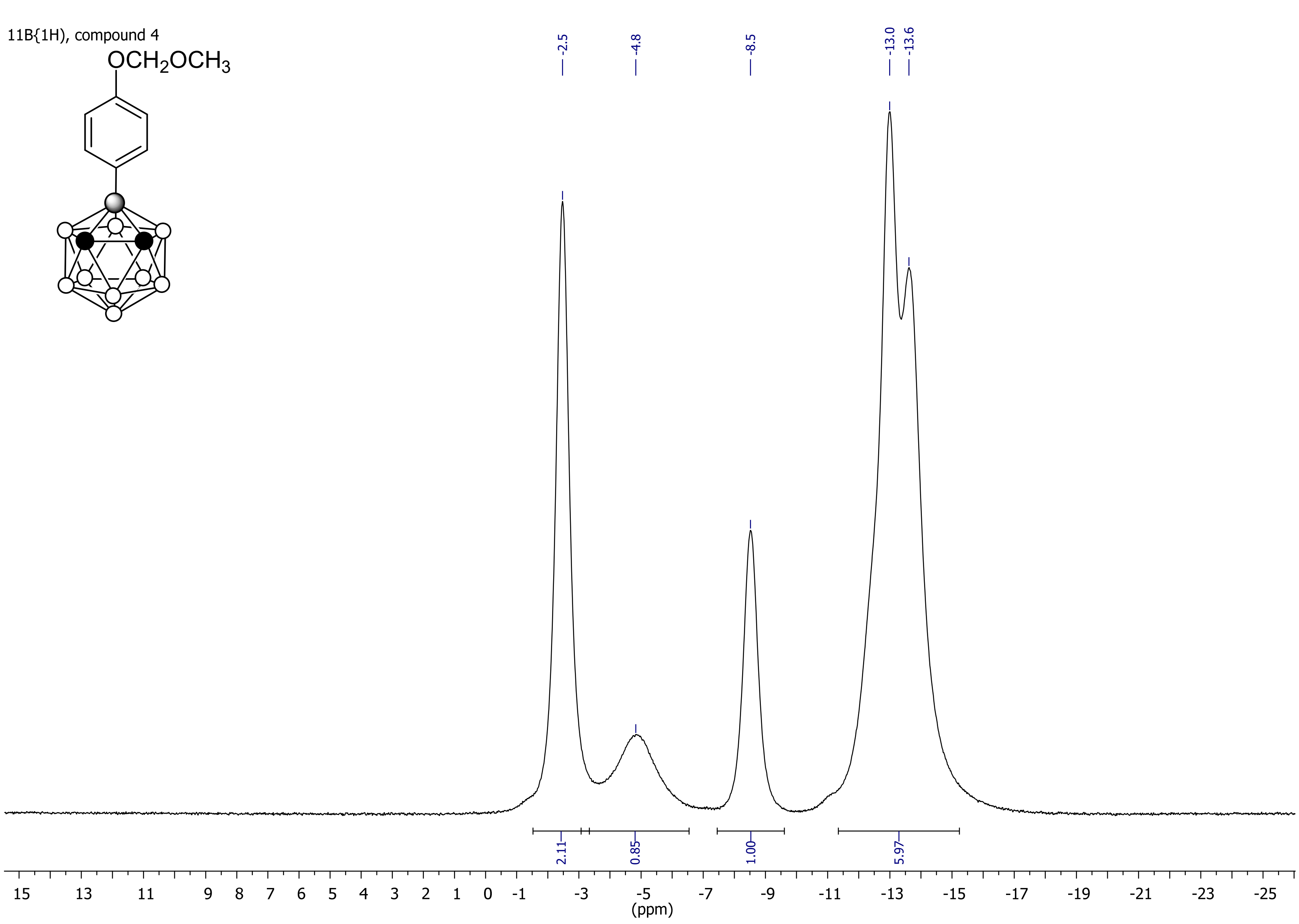
11B{1H}, compound 4
OCH_2OCH_3
-2.5
-4.8
-8.5
-13.0
-13.6
2.11
0.85
1.00
5.97
15 13 11 9 8 7 6 5 4 3 2 1 0 -1 -3 -5 -7 -9 -11 -13 -15 -17 -19 -21 -23 -25
(ppm)

11B, compound 4

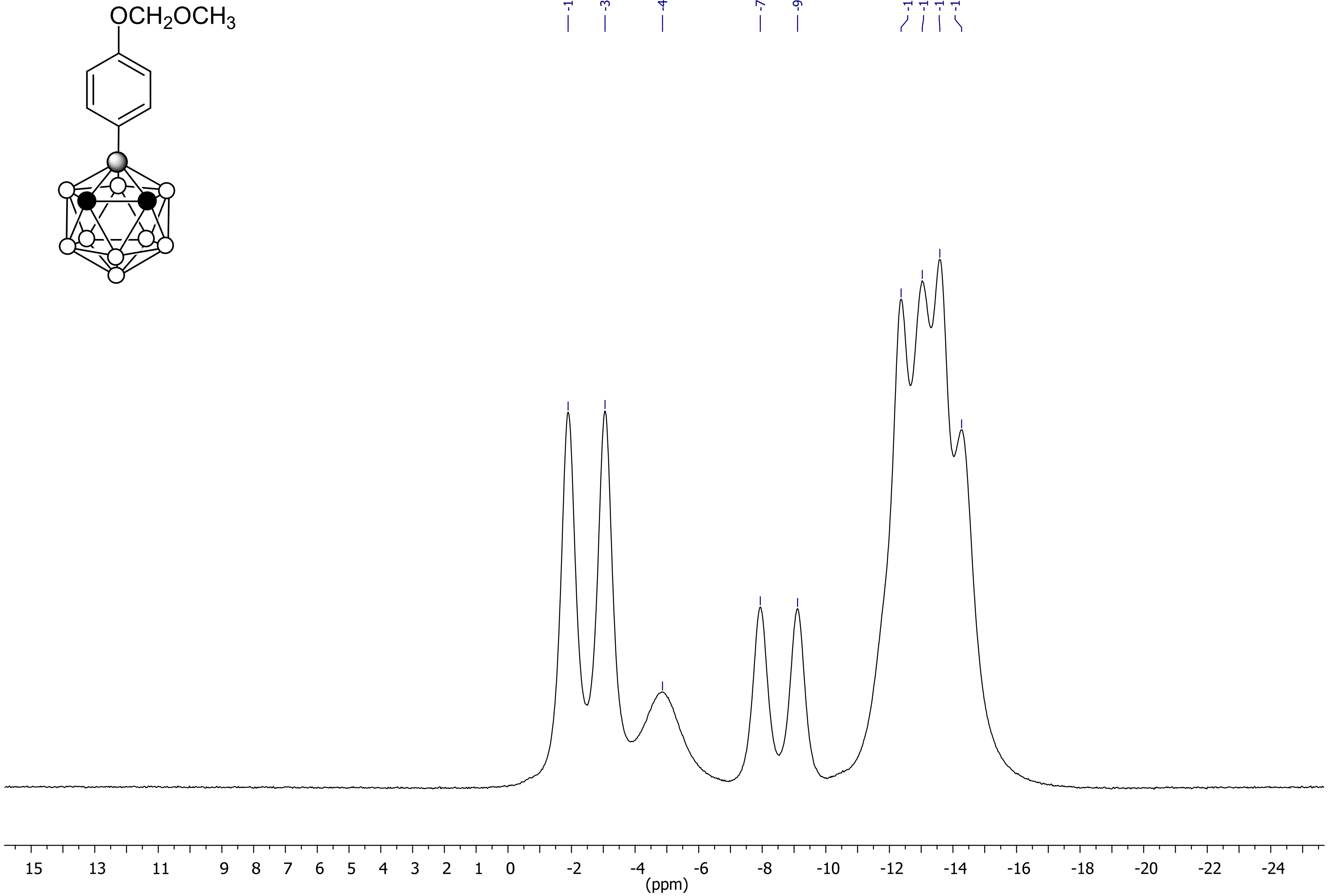

13C{1H), compound 4

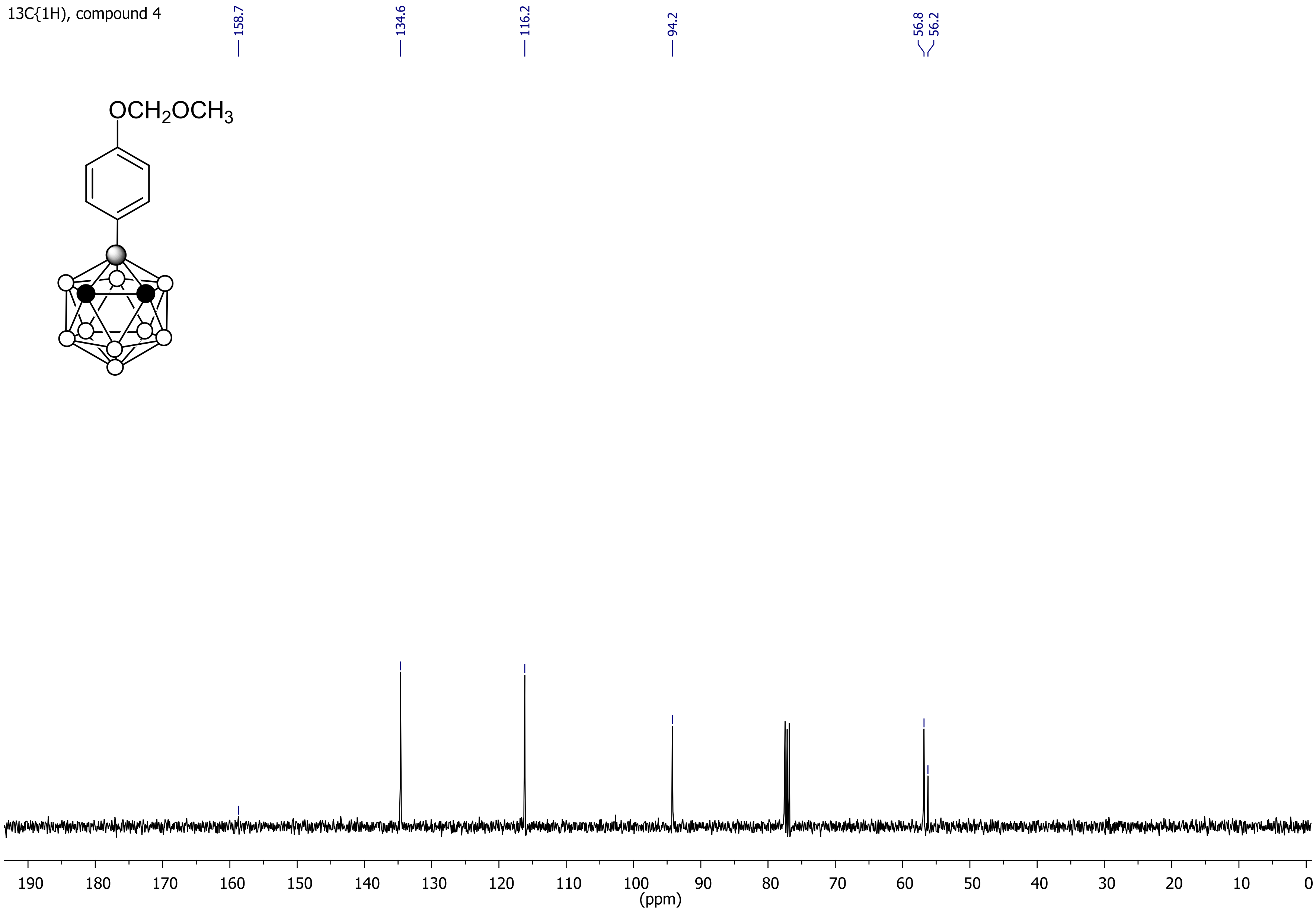

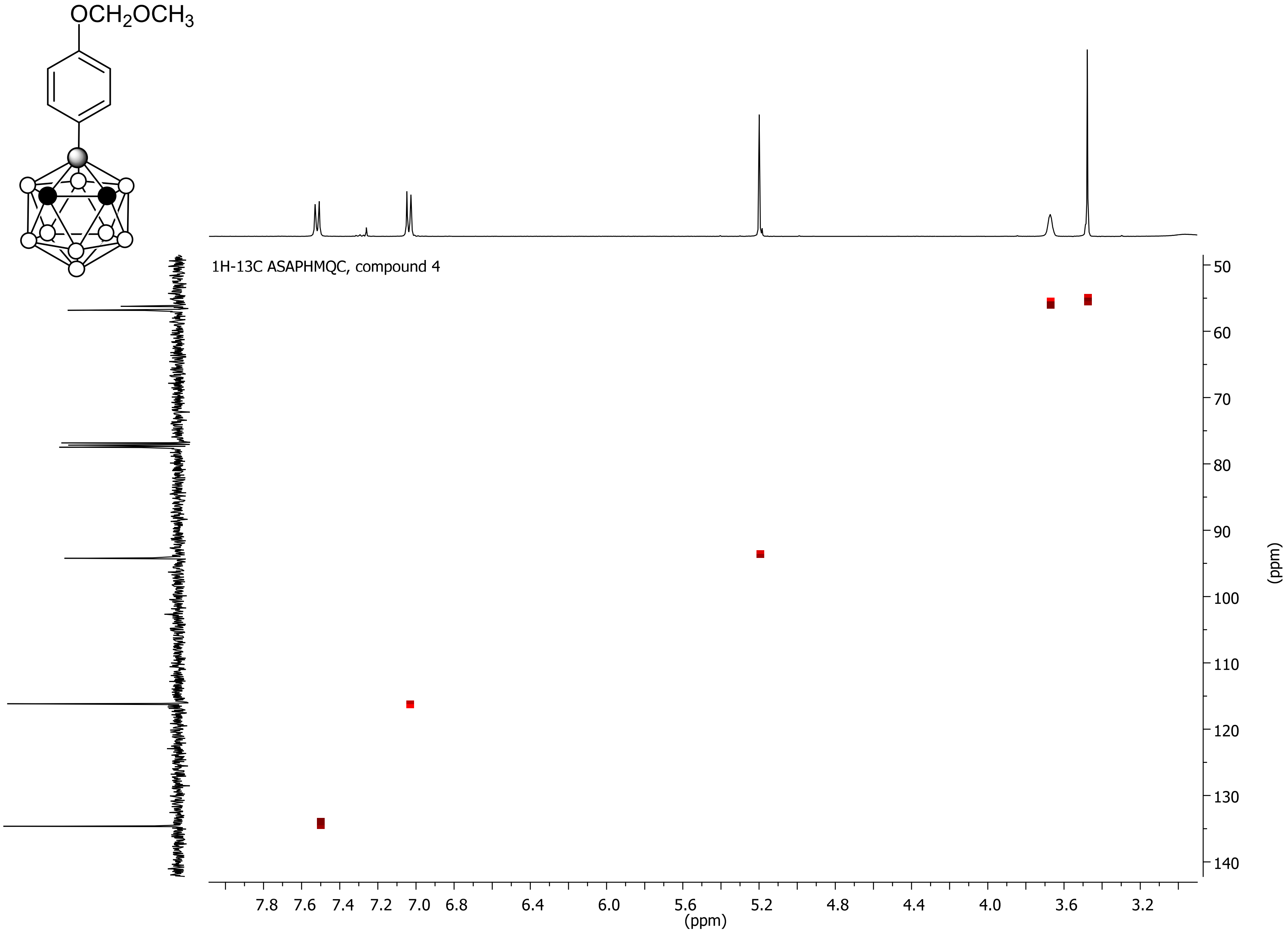
OCH2OCH3
1H-13C ASAPHMQC, compound 4
50
60
70
80
90
100
110
120
130
140
(ppm)
7.8 7.6 7.4 7.2 7.0 6.8 6.4 6.0 5.6 5.2 4.8 4.4 4.0 3.6 3.2
(ppm)

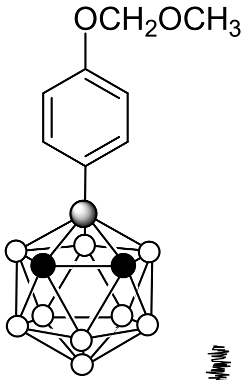

OCH_2OCH_3

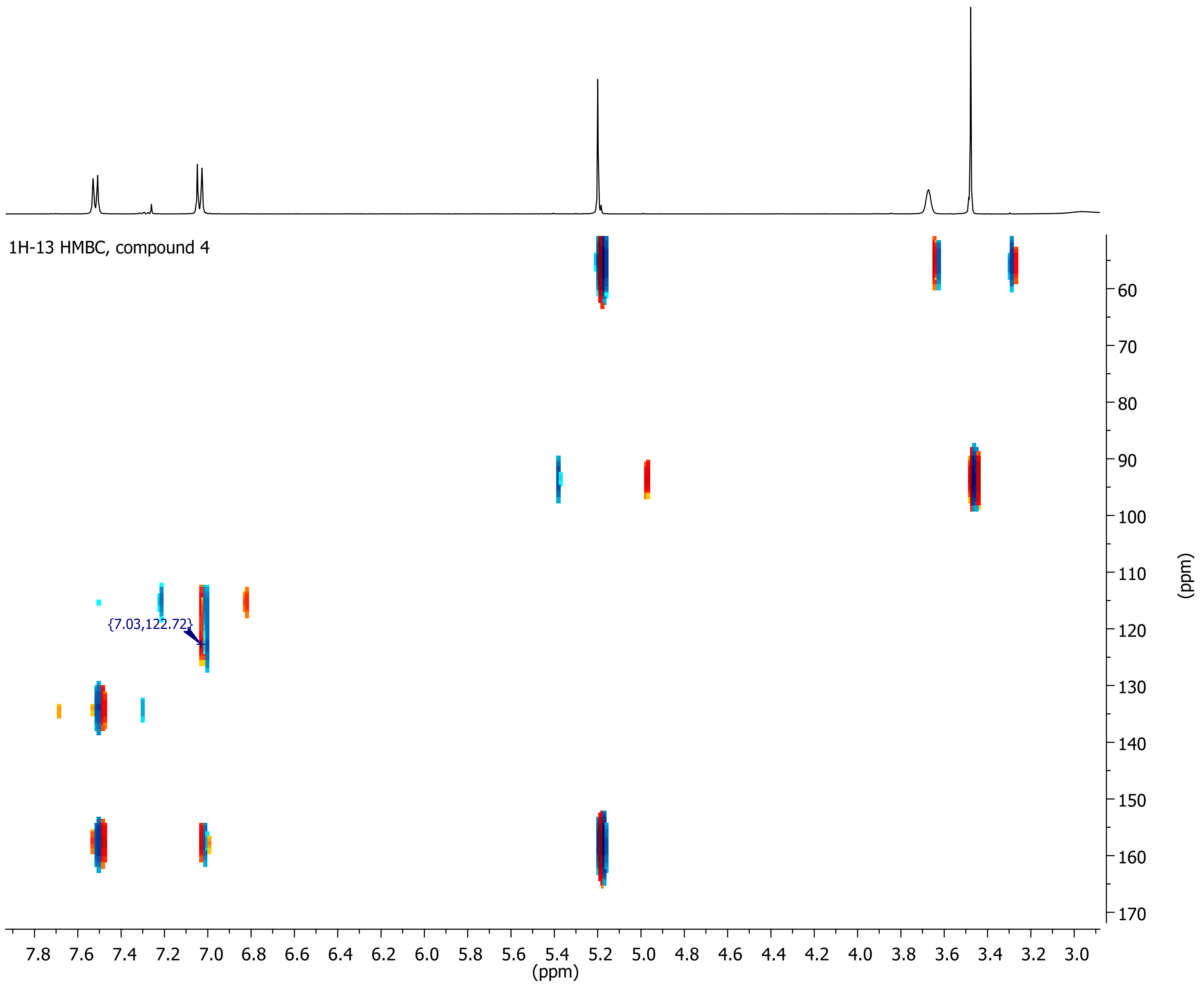

1H-13 HMBC, compound 4
{7.03,122.72}
(ppm)
(ppm)

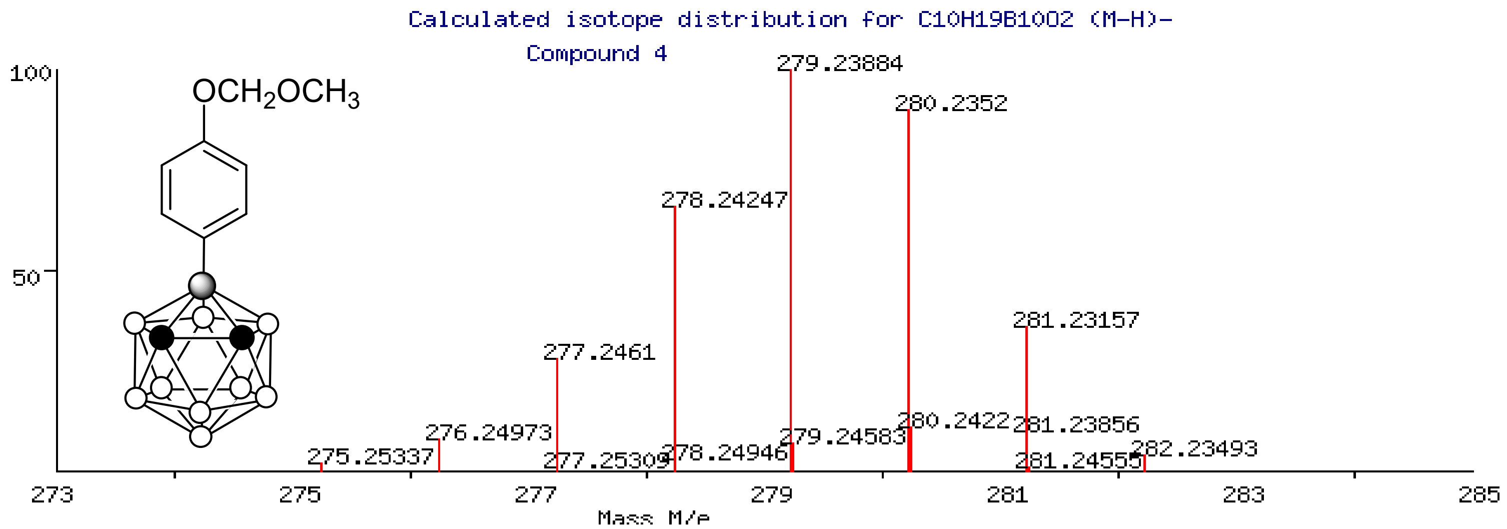
Calculated isotope distribution for C10H19B10O2 (M-H)-
Compound 4
OCH2OCH3
100
50
279.23884
280.2352
278.24247
281.23157
277.2461
276.24973
275.25337
277.25309
278.24946
279.24583
280.2422
281.23856
281.24555
282.23493
273
275
277
279
281
283
285
Mass M/e

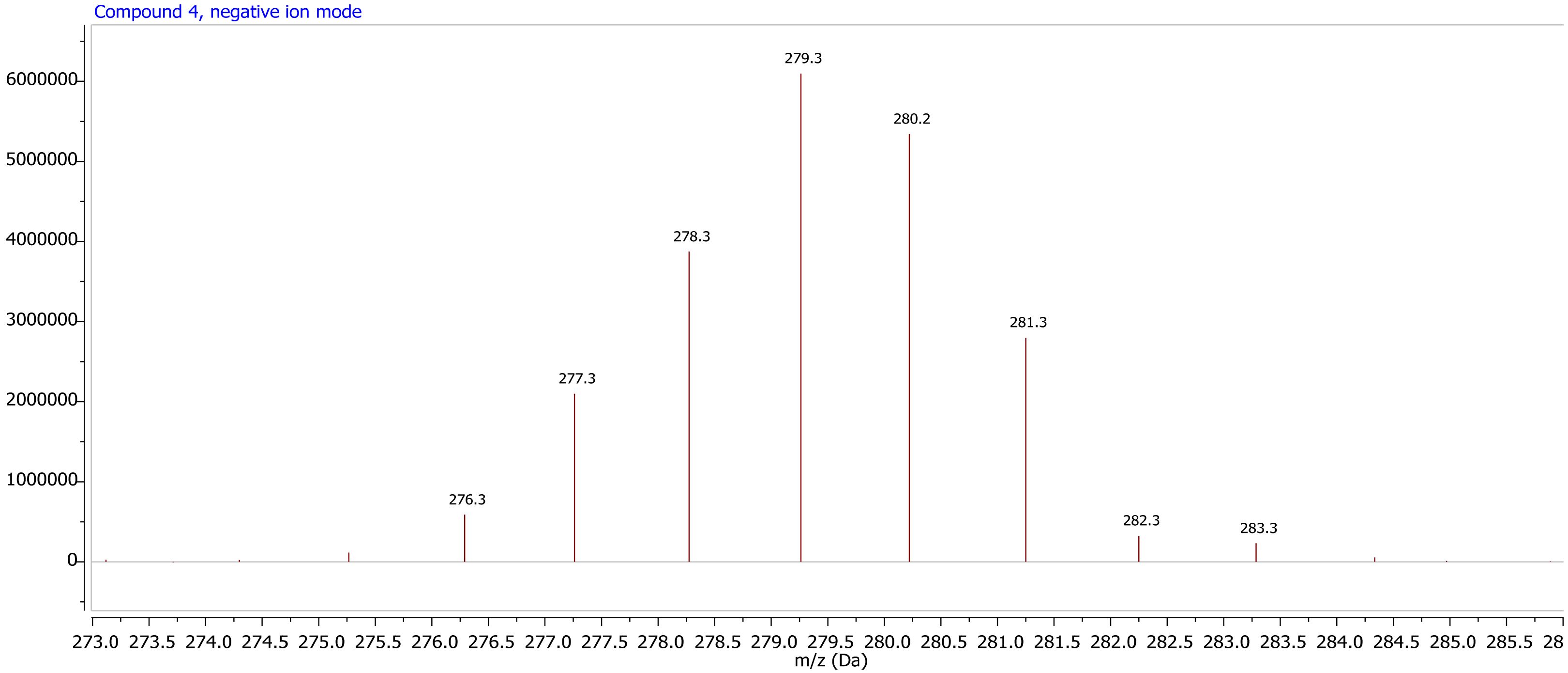
Compound 4, negative ion mode
6000000
5000000
4000000
3000000
2000000
1000000
0
279.3
280.2
278.3
281.3
277.3
276.3
282.3
283.3
273.0 273.5 274.0 274.5 275.0 275.5 276.0 276.5 277.0 277.5 278.0 278.5 279.0 279.5 280.0 280.5 281.0 281.5 282.0 282.5 283.0 283.5 284.0 284.5 285.0 285.5 28
m/z (Da)

1H, compound 5

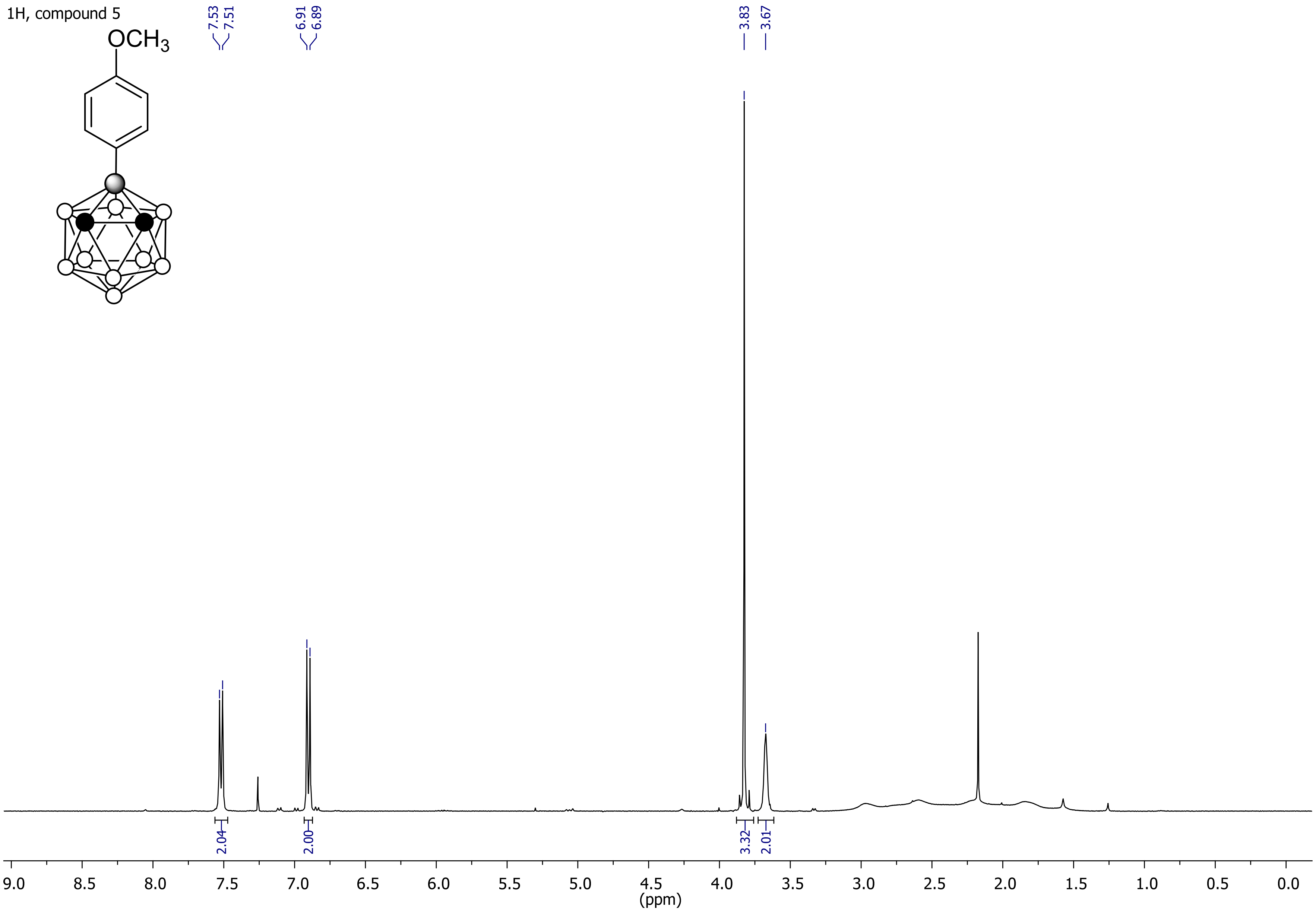

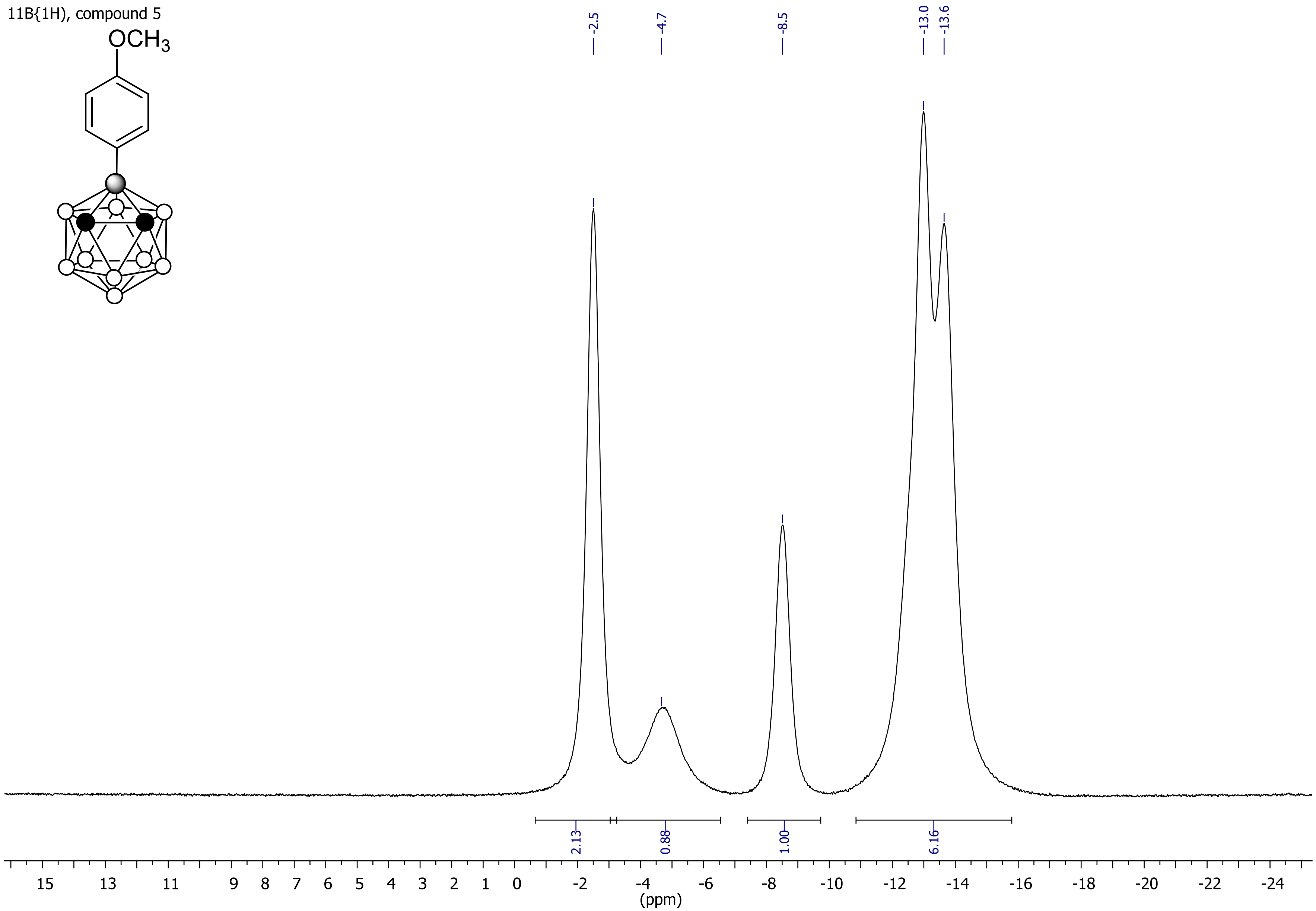
11B{1H}, compound 5
OCH3
-2.5
-4.7
-8.5
-13.0
-13.6
2.13
0.88
1.00
6.16
15 13 11 9 8 7 6 5 4 3 2 1 0 -2 -4 -6 -8 -10 -12 -14 -16 -18 -20 -22 -24
(ppm)

11B, compound 5

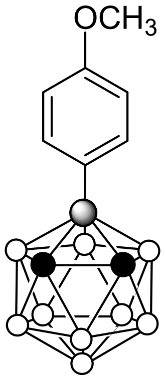

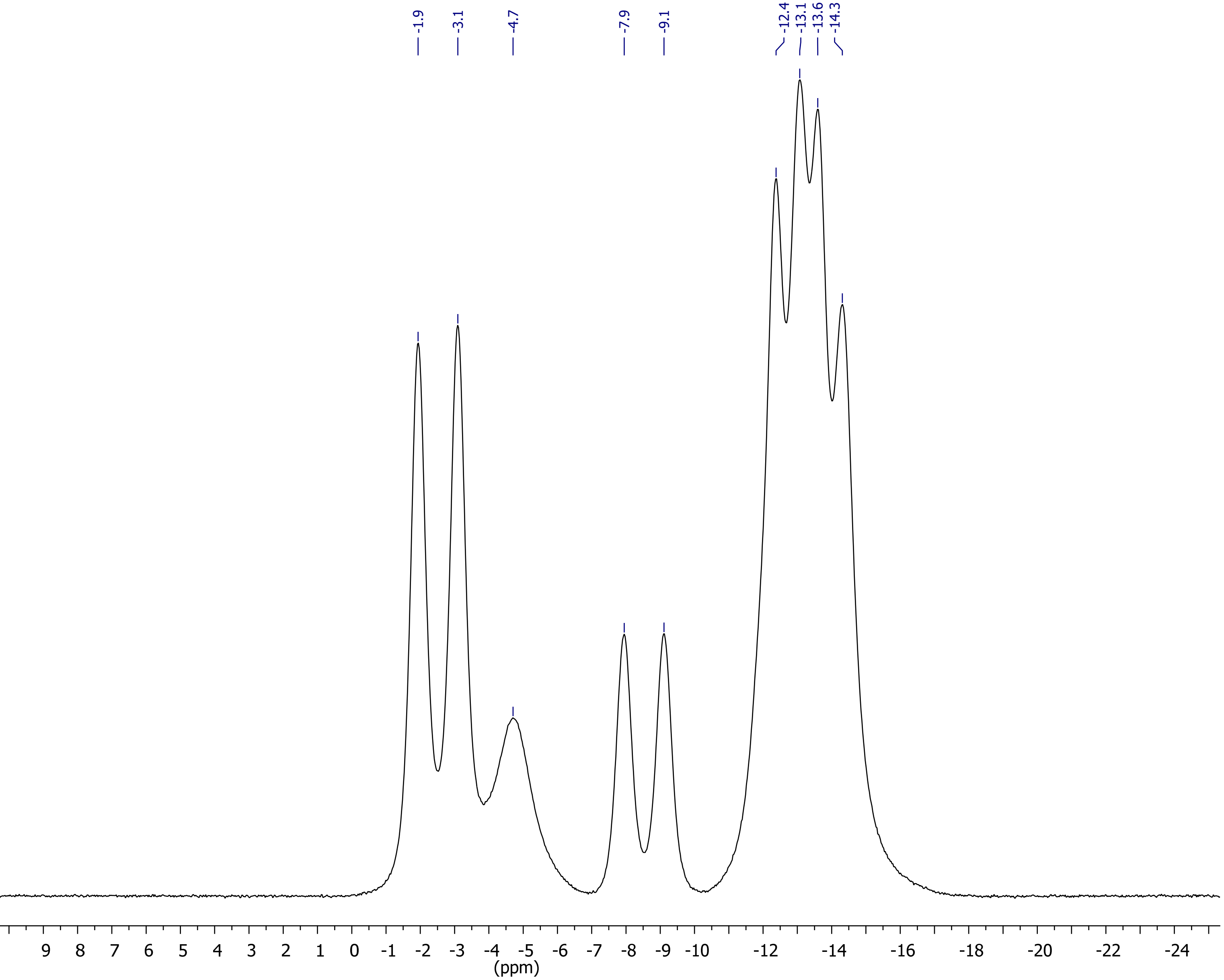

13C{1H}, compound 5

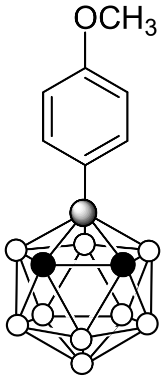

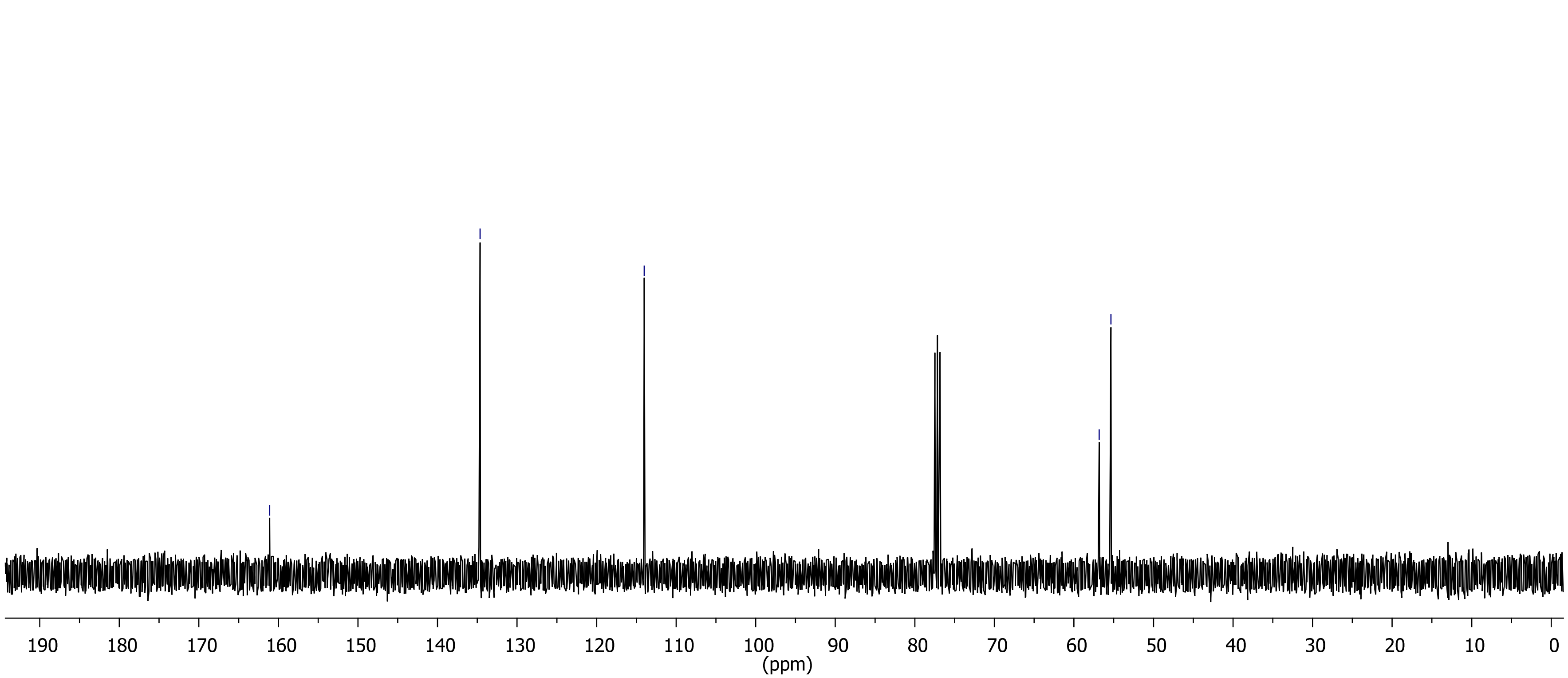

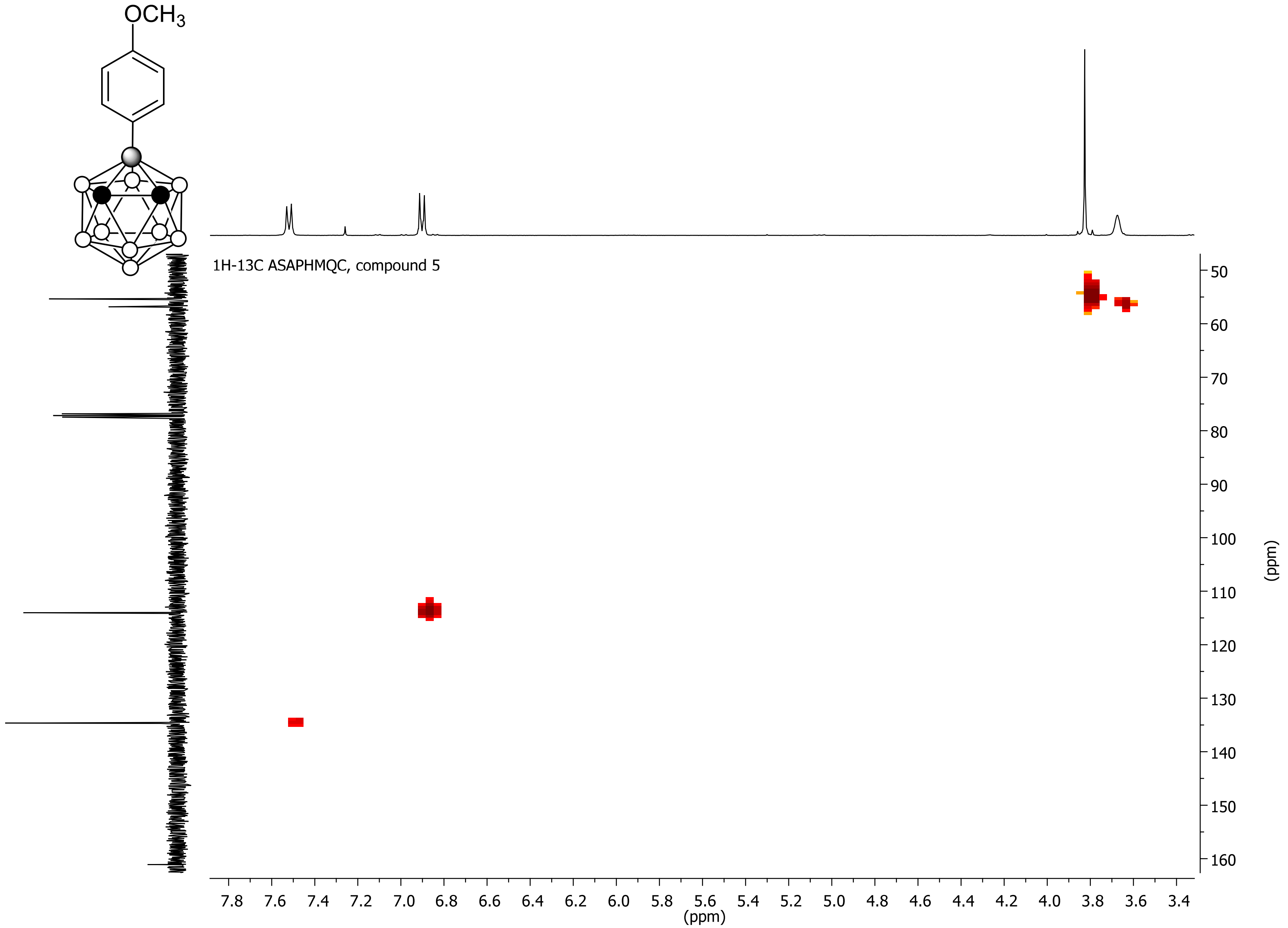
OCH3
1H-13C ASAPHMQC, compound 5
7.8
7.6
7.4
7.2
7.0
6.8
6.6
6.4
6.2
6.0
5.8
5.6
5.4
5.2
5.0
4.8
4.6
4.4
4.2
4.0
3.8
3.6
3.4
(ppm)
50
60
70
80
90
100
110
120
130
140
150
160
(ppm)

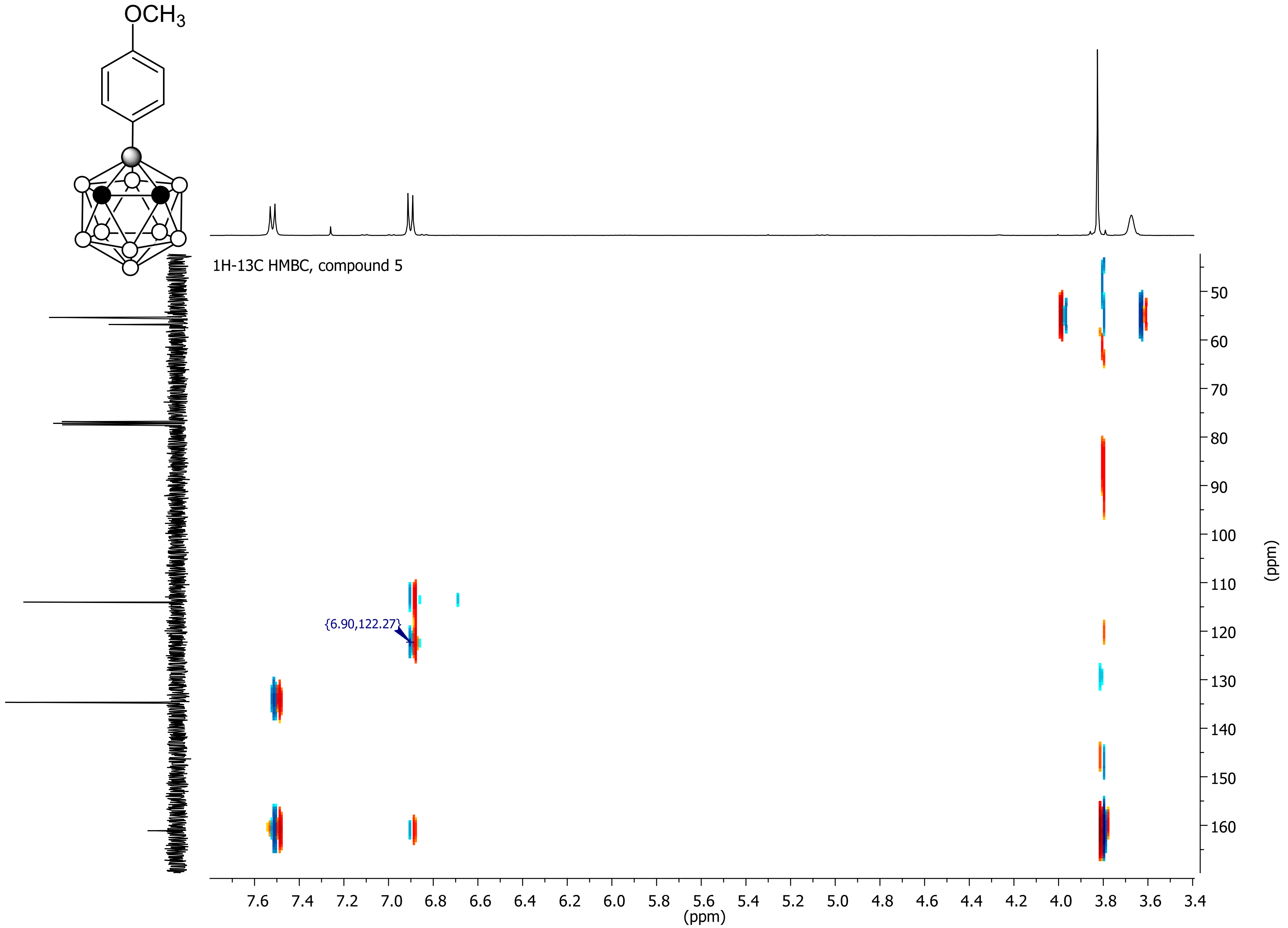
OCH3
1H-13C HMBC, compound 5
{6.90,122.27}
(ppm)
(ppm)

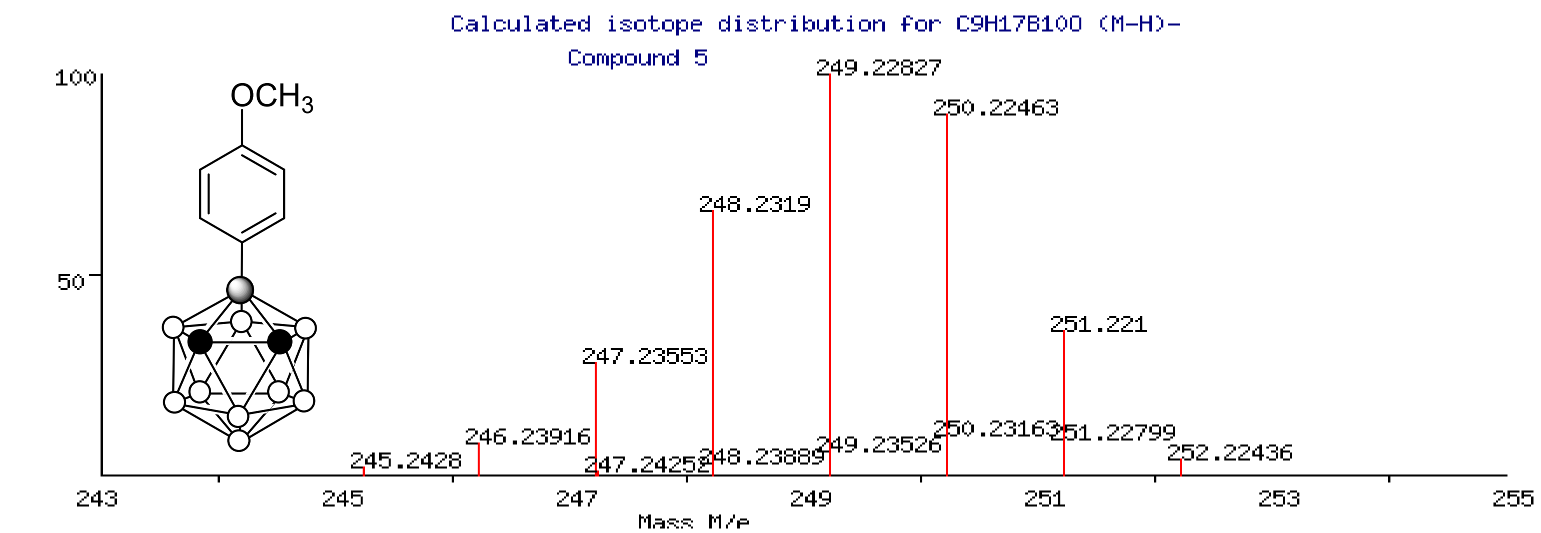
Calculated isotope distribution for C9H17B10O (M-H)-
Compound 5
OCH3
100
50
249.22827
250.22463
248.2319
251.221
247.23553
246.23916
245.2428
247.24252
248.23889
249.23526
250.23163
251.22799
252.22436
243
245
247
249
251
253
255
Mass M/e

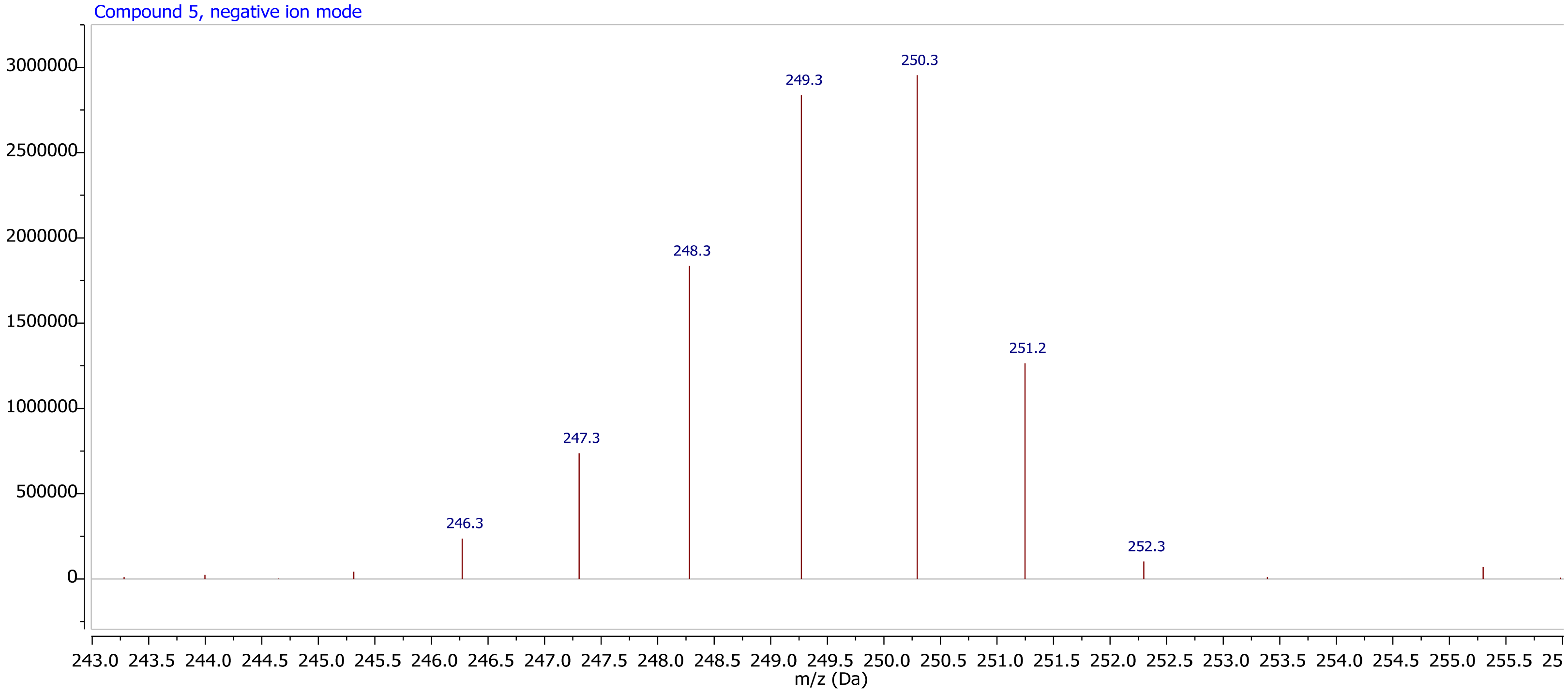
Compound 5, negative ion mode
3000000
2500000
2000000
1500000
1000000
500000
0
249.3
250.3
248.3
251.2
247.3
246.3
252.3
243.0 243.5 244.0 244.5 245.0 245.5 246.0 246.5 247.0 247.5 248.0 248.5 249.0 249.5 250.0 250.5 251.0 251.5 252.0 252.5 253.0 253.5 254.0 254.5 255.0 255.5 25
m/z (Da)

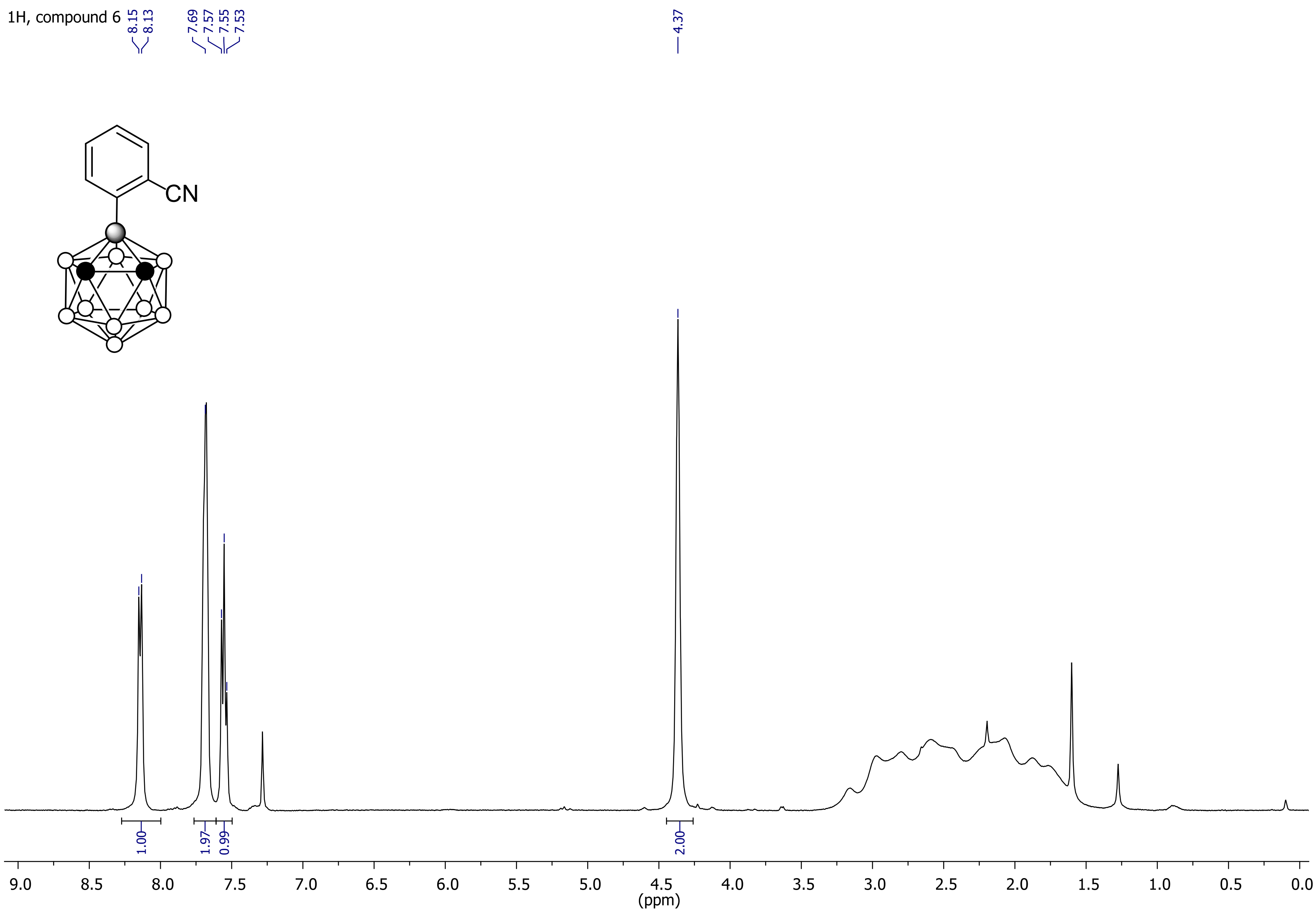
1H, compound 6
8.15
8.13
7.69
7.57
7.55
7.53
4.37
CN
1.00
1.97
0.99
2.00
9.0 8.5 8.0 7.5 7.0 6.5 6.0 5.5 5.0 4.5 4.0 3.5 3.0 2.5 2.0 1.5 1.0 0.5 0.0
(ppm)

11B{1H}, compound 6

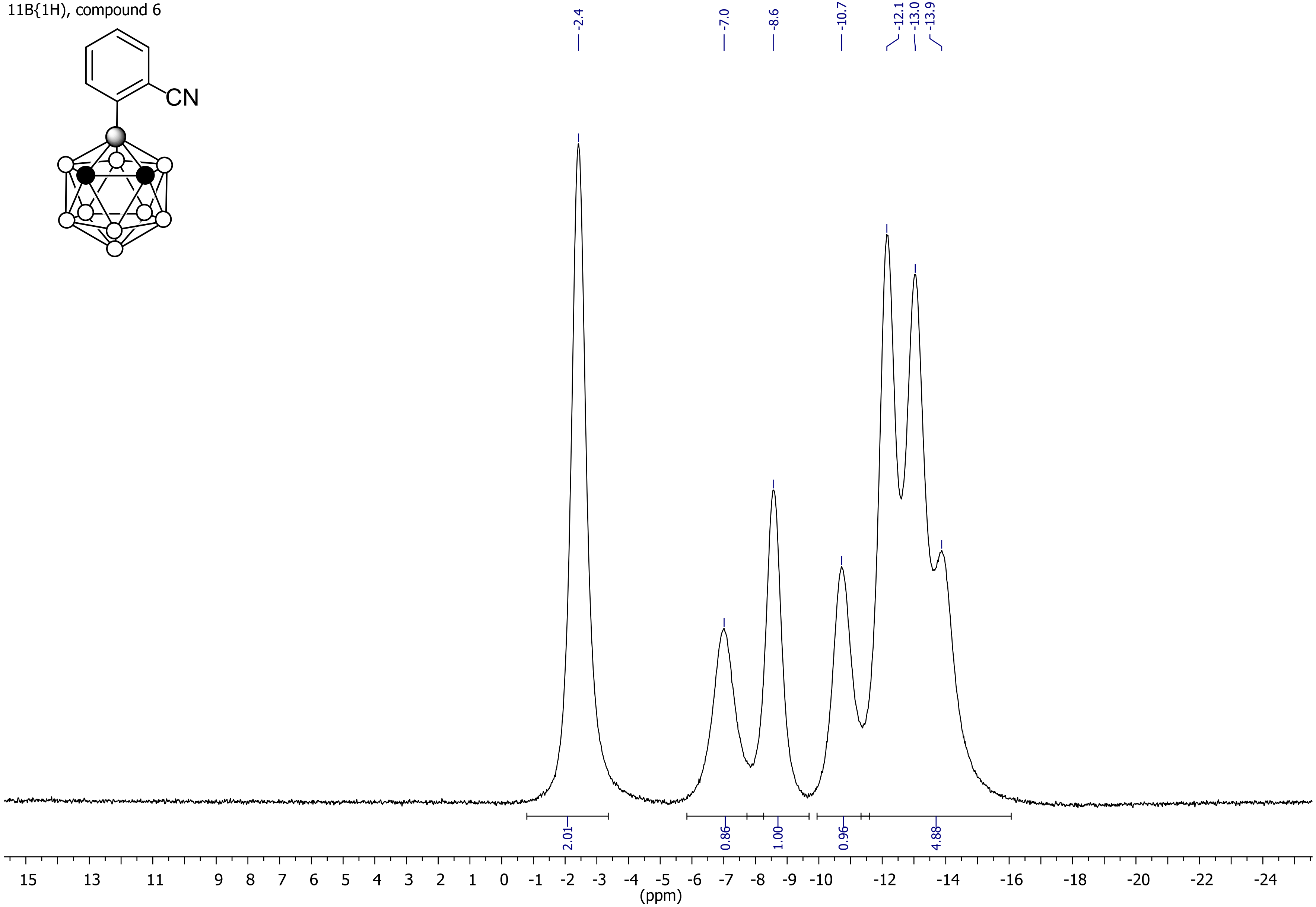

11B, compound 6

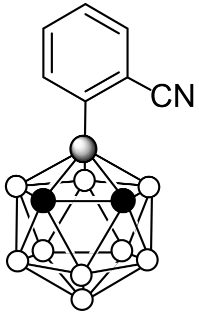

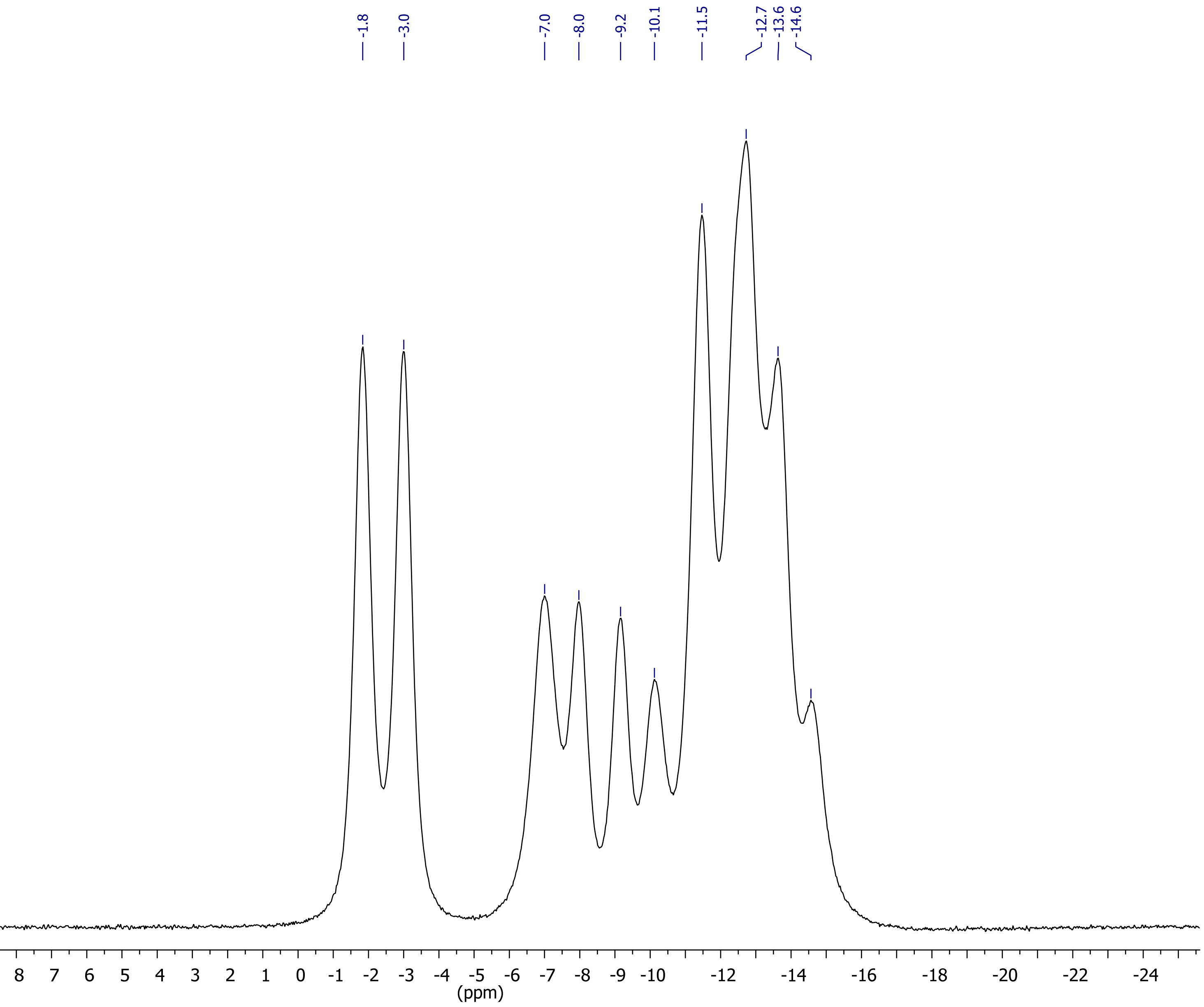

13C{1H}, compound 6

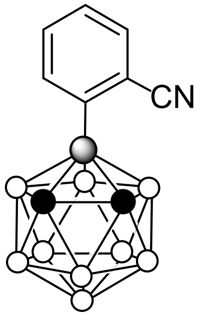

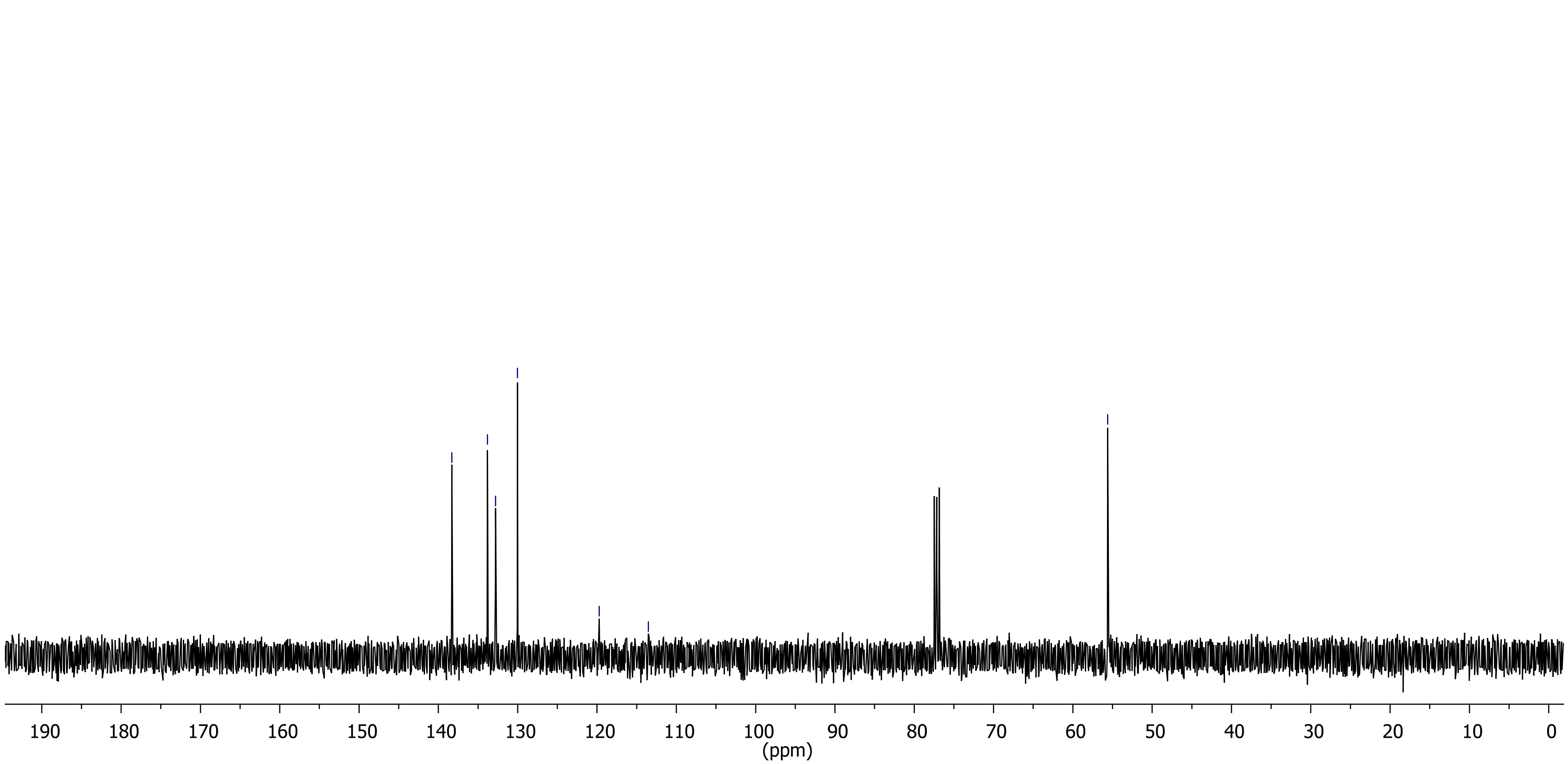

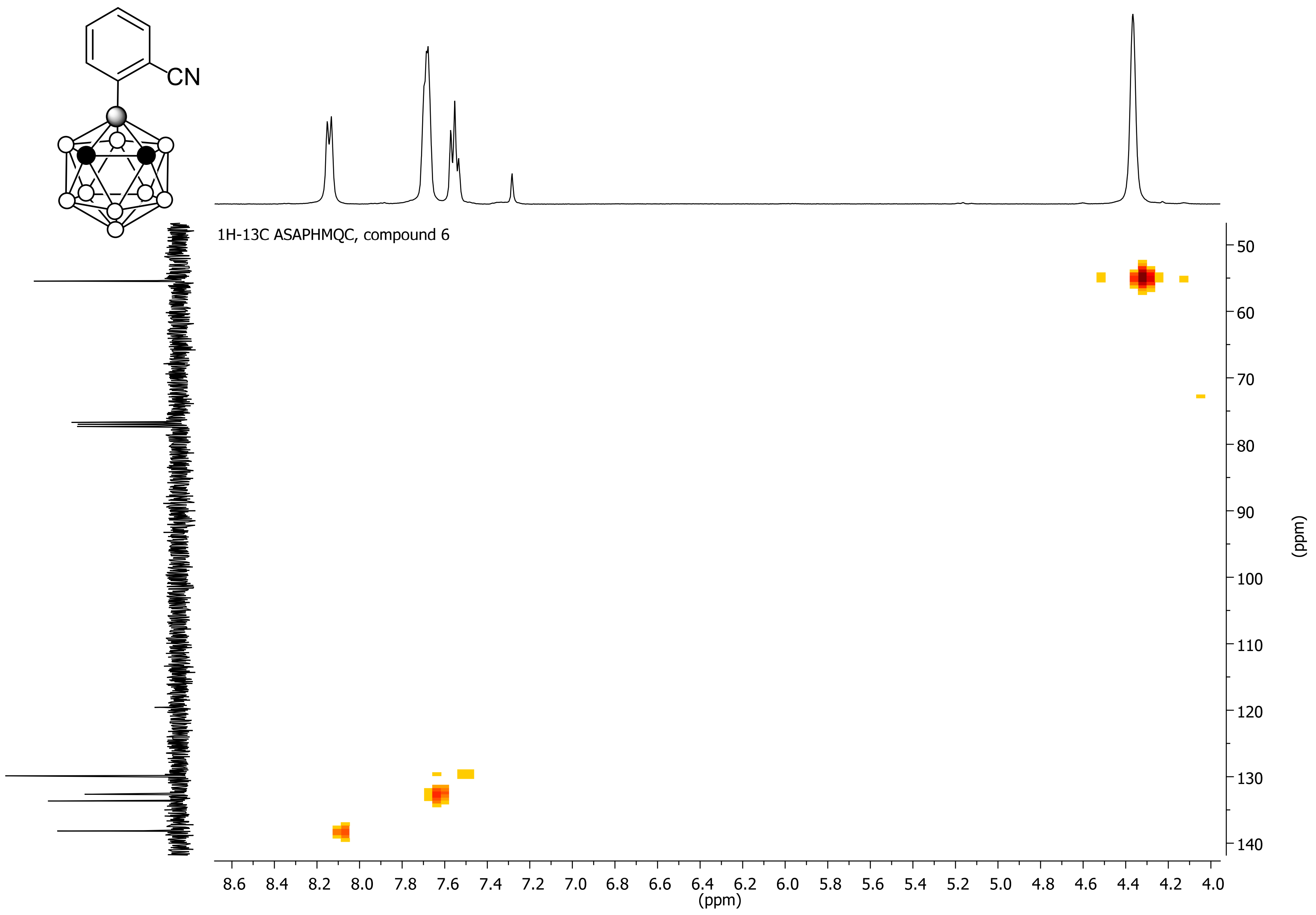
CN
1H-13C ASAPHMQC, compound 6
50
60
70
80
90
100
110
120
130
140
(ppm)
8.6 8.4 8.2 8.0 7.8 7.6 7.4 7.2 7.0 6.8 6.6 6.4 6.2 6.0 5.8 5.6 5.4 5.2 5.0 4.8 4.6 4.4 4.2 4.0
(ppm)

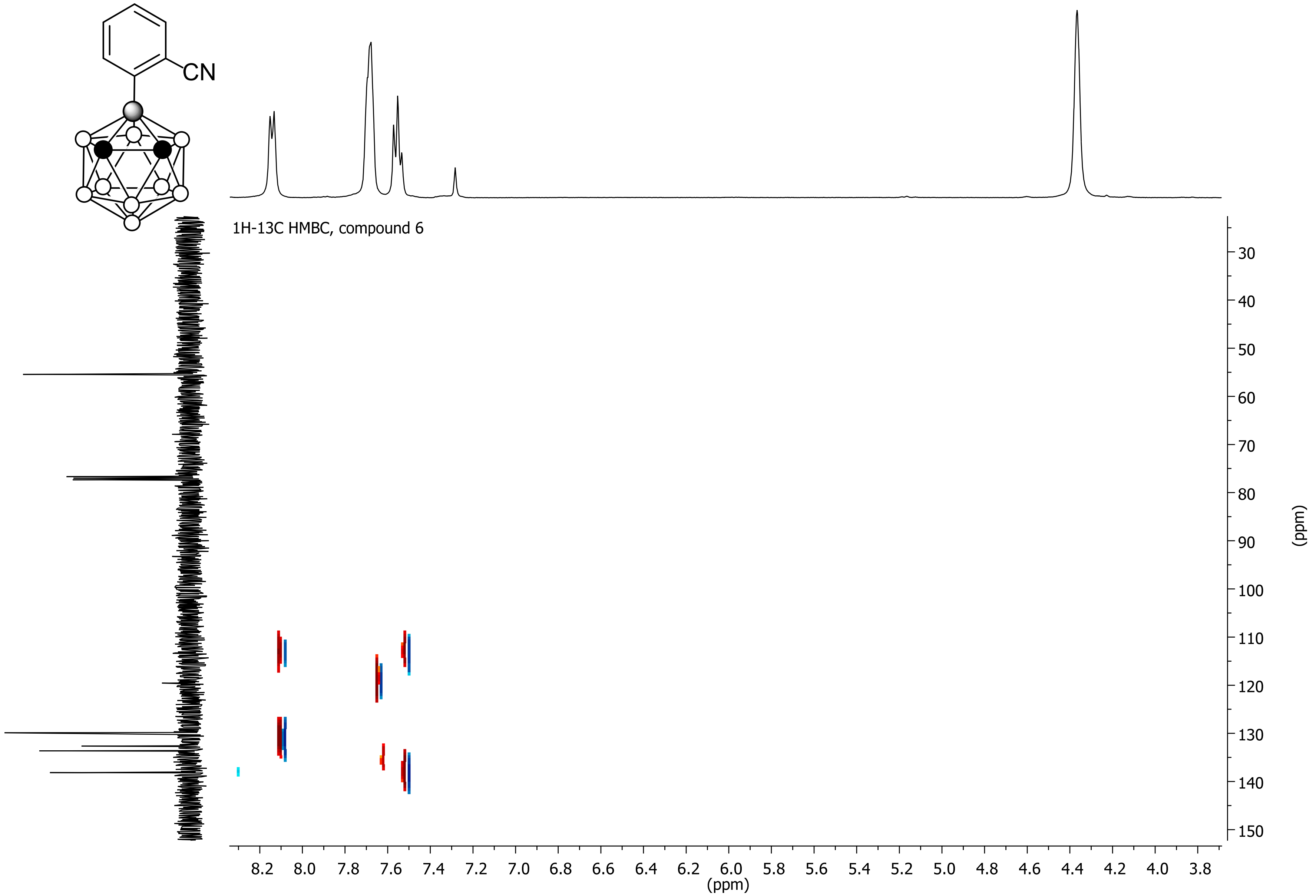
CN
1H-13C HMBC, compound 6
30
40
50
60
70
80
90
100
110
120
130
140
150
(ppm)
8.2 8.0 7.8 7.6 7.4 7.2 7.0 6.8 6.6 6.4 6.2 6.0 5.8 5.6 5.4 5.2 5.0 4.8 4.6 4.4 4.2 4.0 3.8
(ppm)

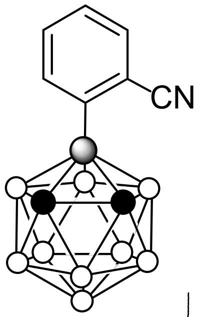

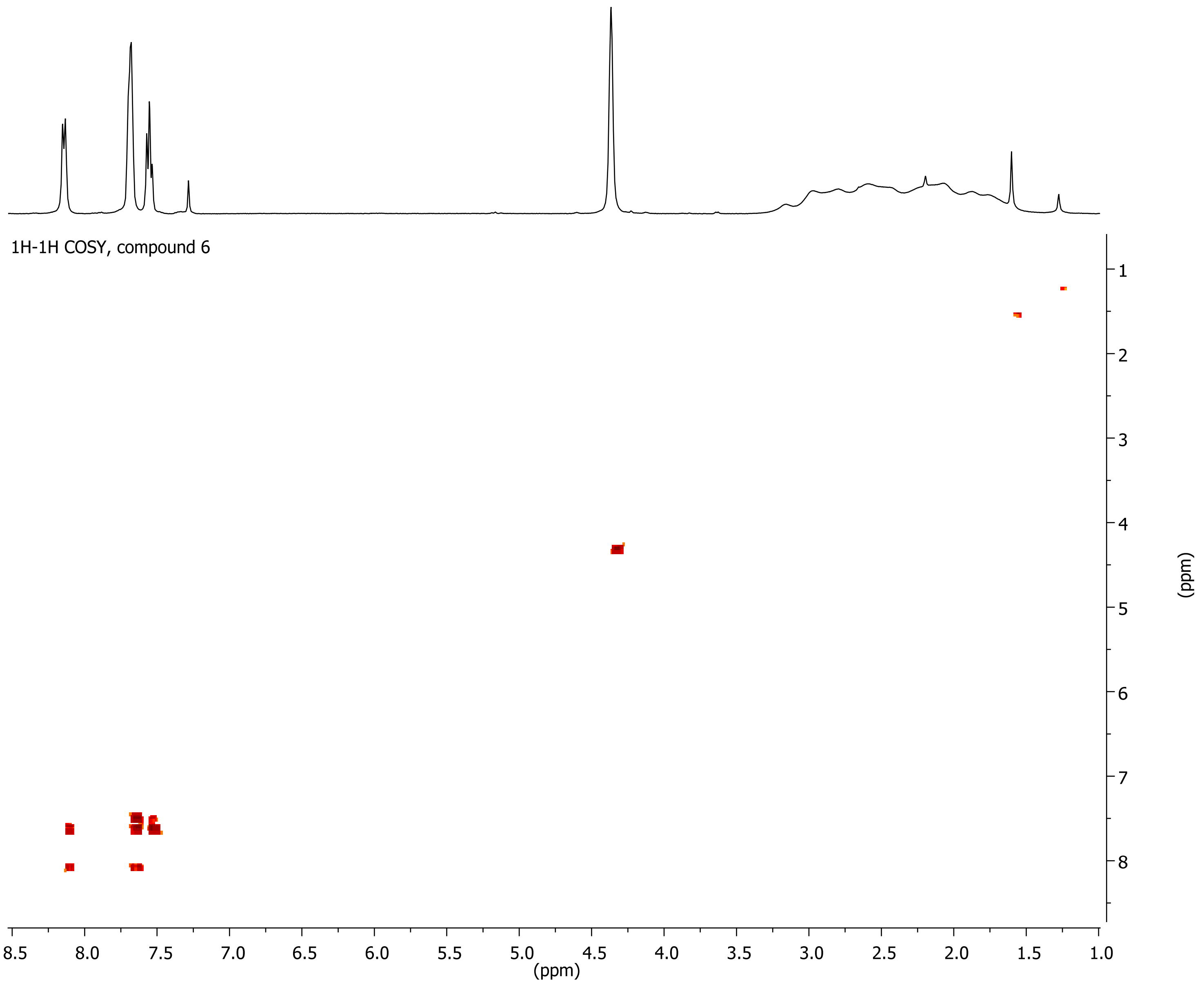

1H-1H COSY, compound 6
8.5
8.0
7.5
7.0
6.5
6.0
5.5
5.0
4.5
4.0
3.5
3.0
2.5
2.0
1.5
1.0
(ppm)
1
2
3
4
5
6
7
8
(ppm)

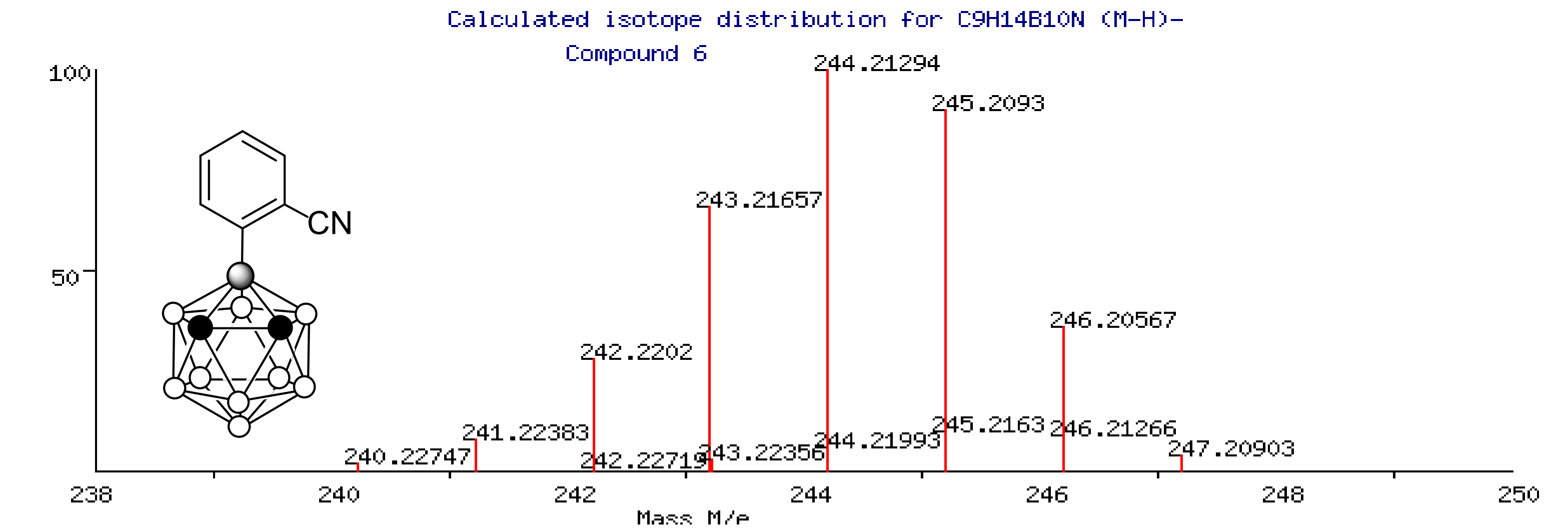
Calculated isotope distribution for C9H14B10N (M-H)-
Compound 6
CN
100
50
240.22747
241.22383
242.2202
242.22719
243.21657
243.22356
244.21294
244.21993
245.2093
245.2163
246.20567
246.21266
247.20903
238
240
242
244
246
248
250
Mass M/e

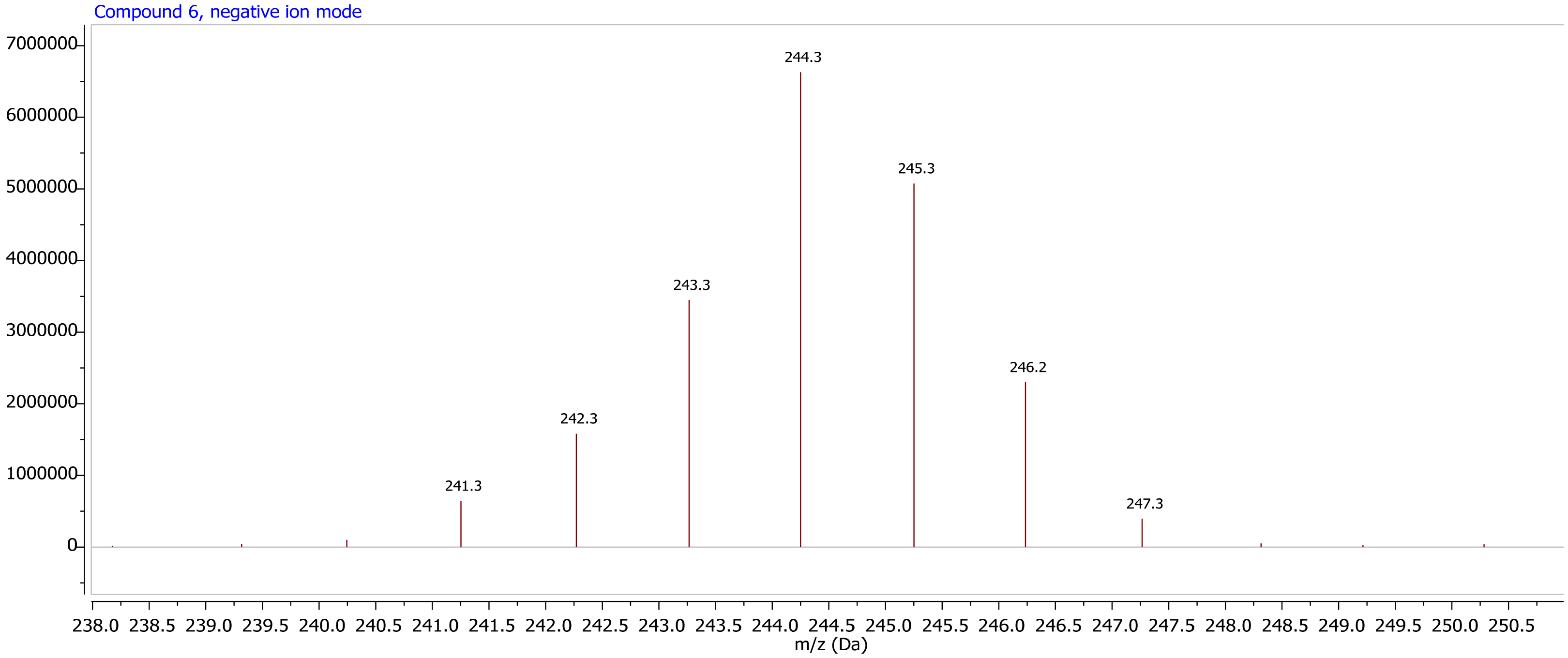
Compound 6, negative ion mode
7000000
6000000
5000000
4000000
3000000
2000000
1000000
0
241.3
242.3
243.3
244.3
245.3
246.2
247.3
238.0 238.5 239.0 239.5 240.0 240.5 241.0 241.5 242.0 242.5 243.0 243.5 244.0 244.5 245.0 245.5 246.0 246.5 247.0 247.5 248.0 248.5 249.0 249.5 250.0 250.5
m/z (Da)

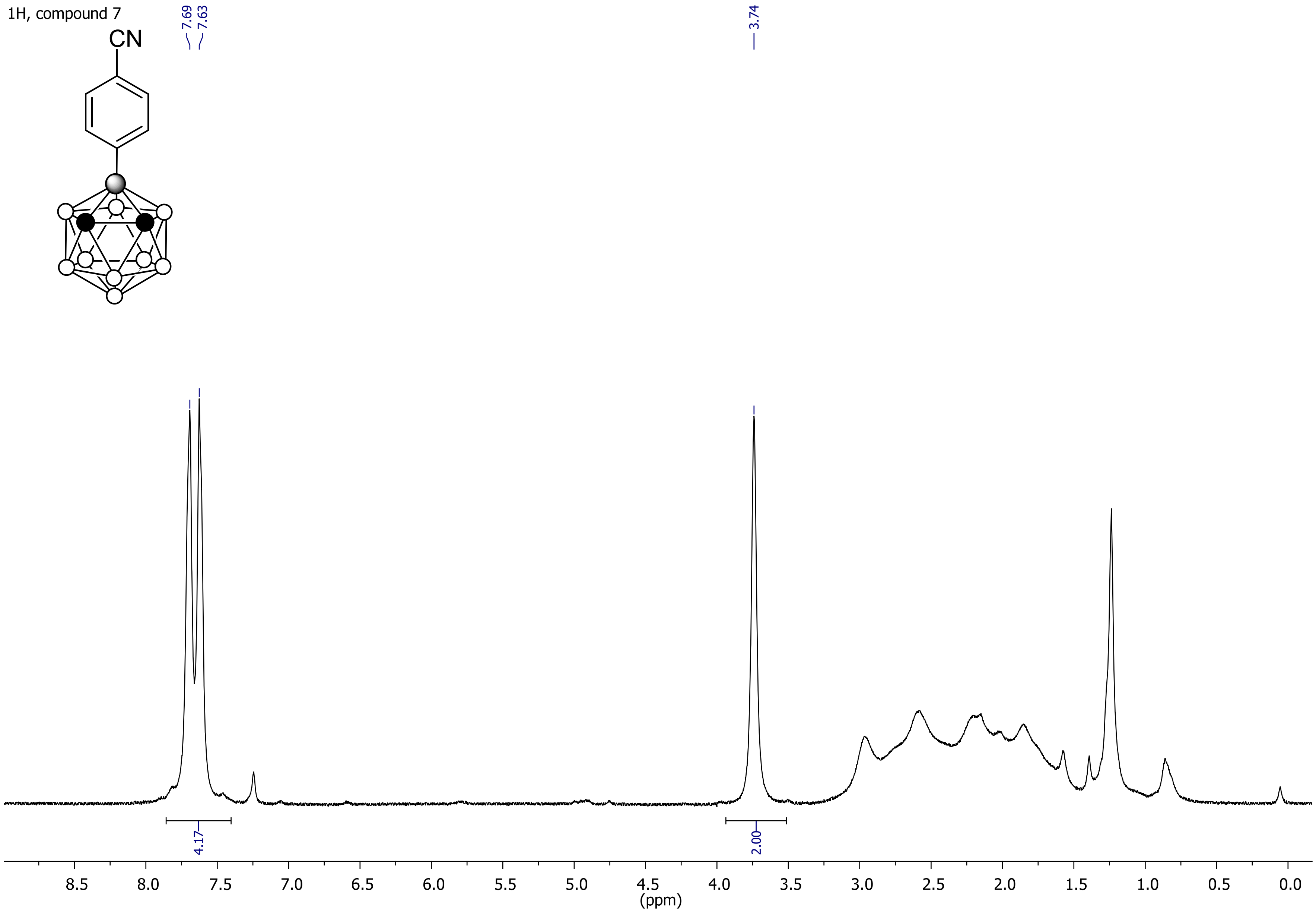

1H, compound 7
CN
7.69
7.63
3.74
4.17
2.00
8.5 8.0 7.5 7.0 6.5 6.0 5.5 5.0 4.5 4.0 3.5 3.0 2.5 2.0 1.5 1.0 0.5 0.0
(ppm)

11B{1H}, compound 7

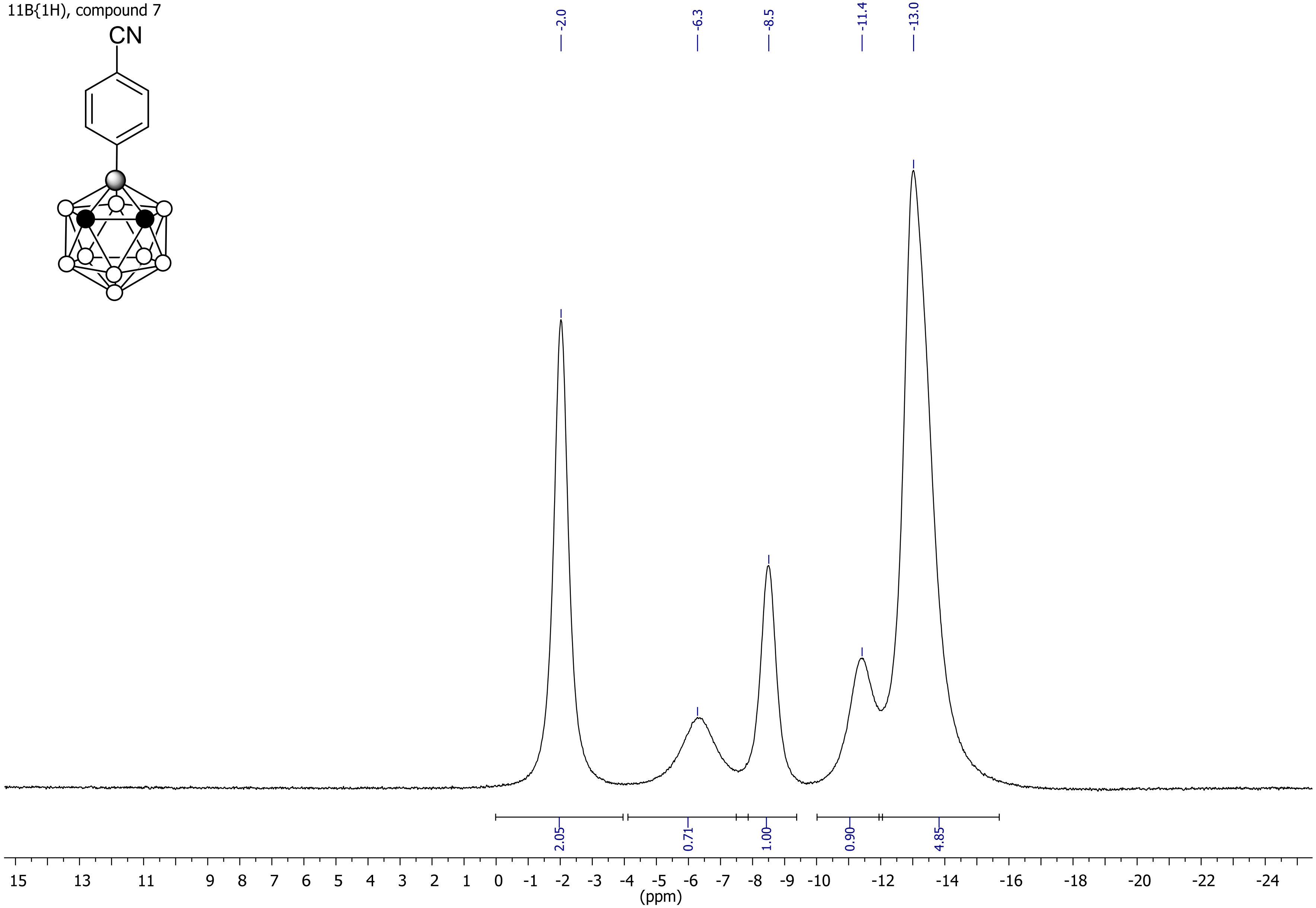

11B, compound 7

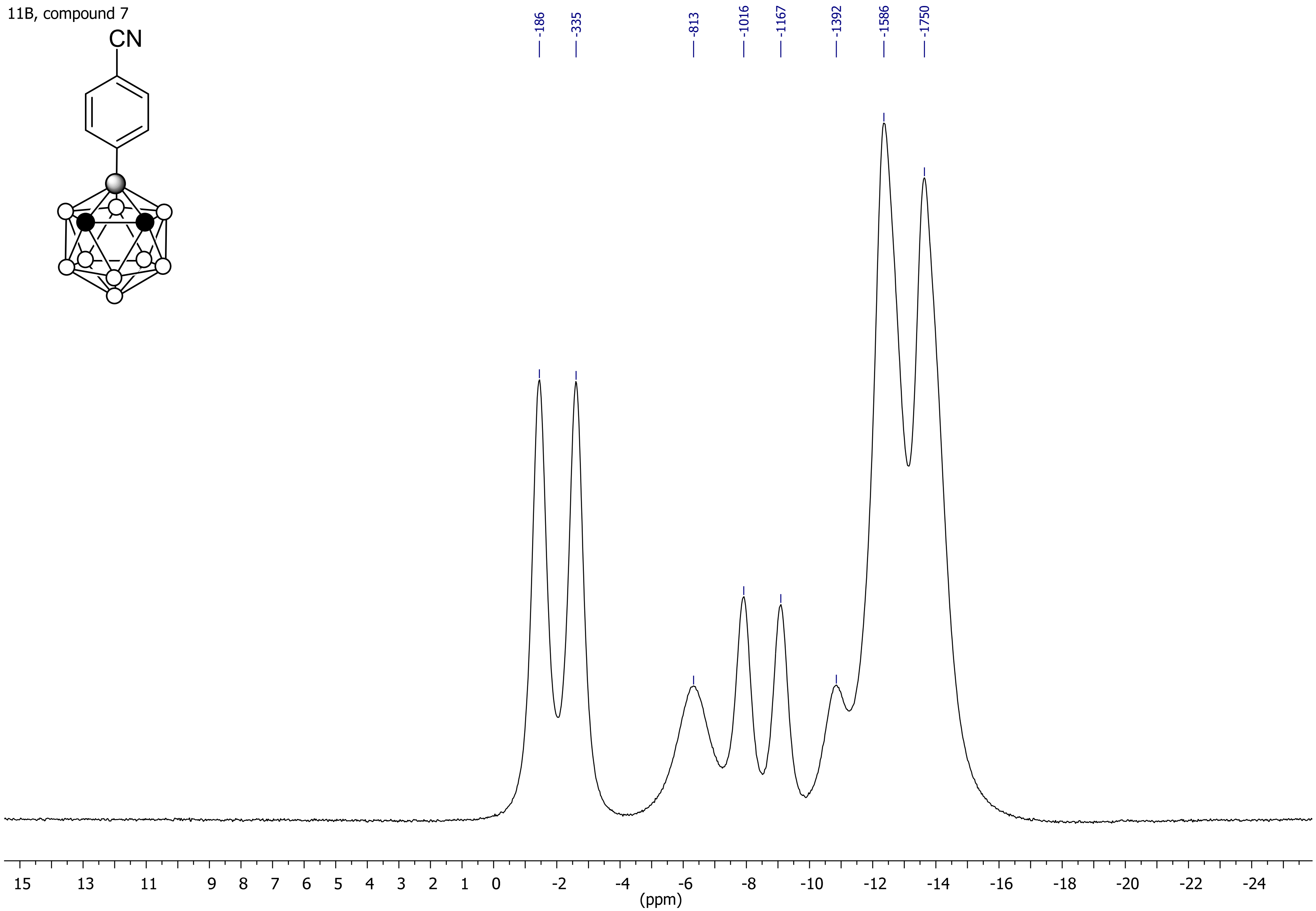

13C{1H}, compound 7

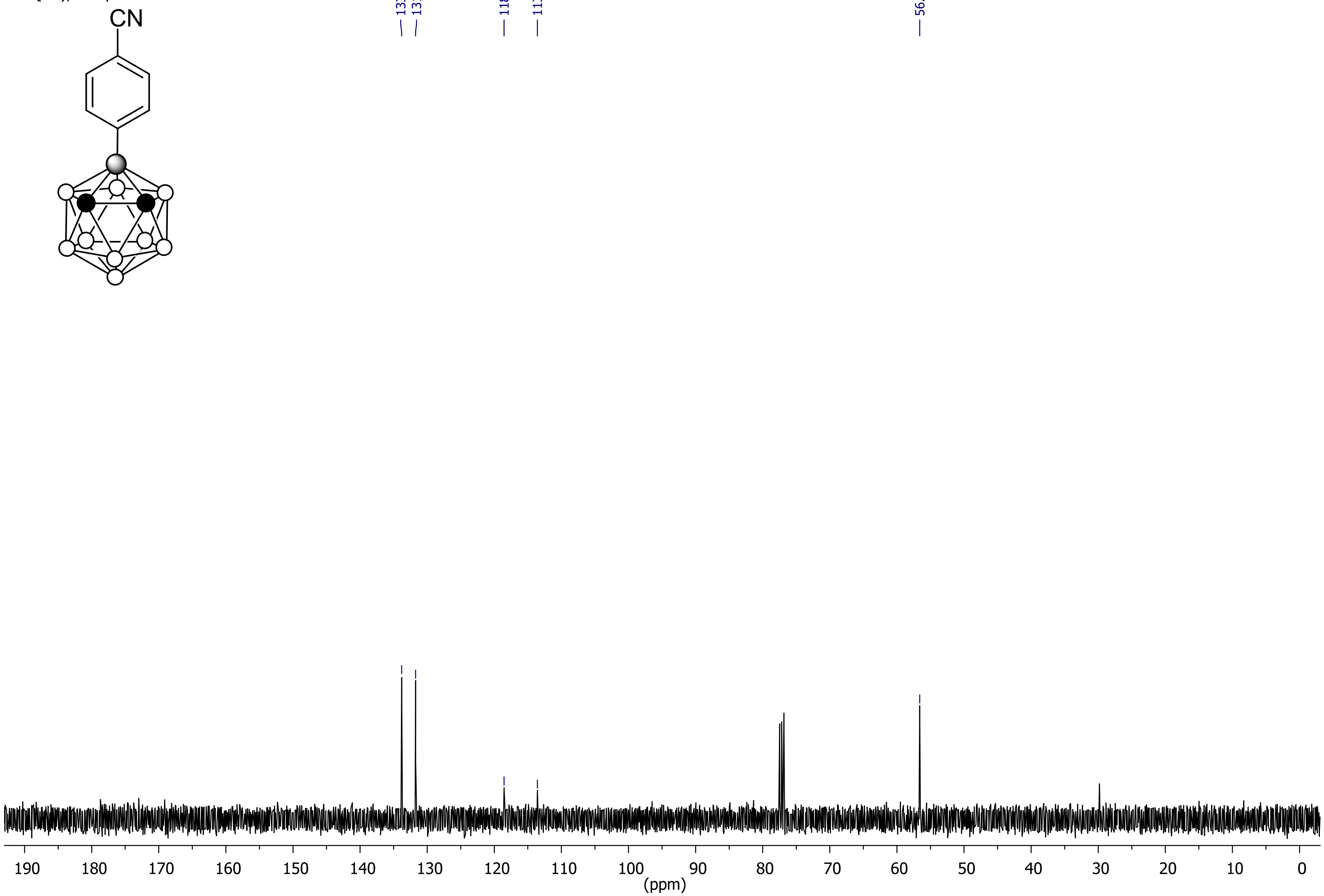

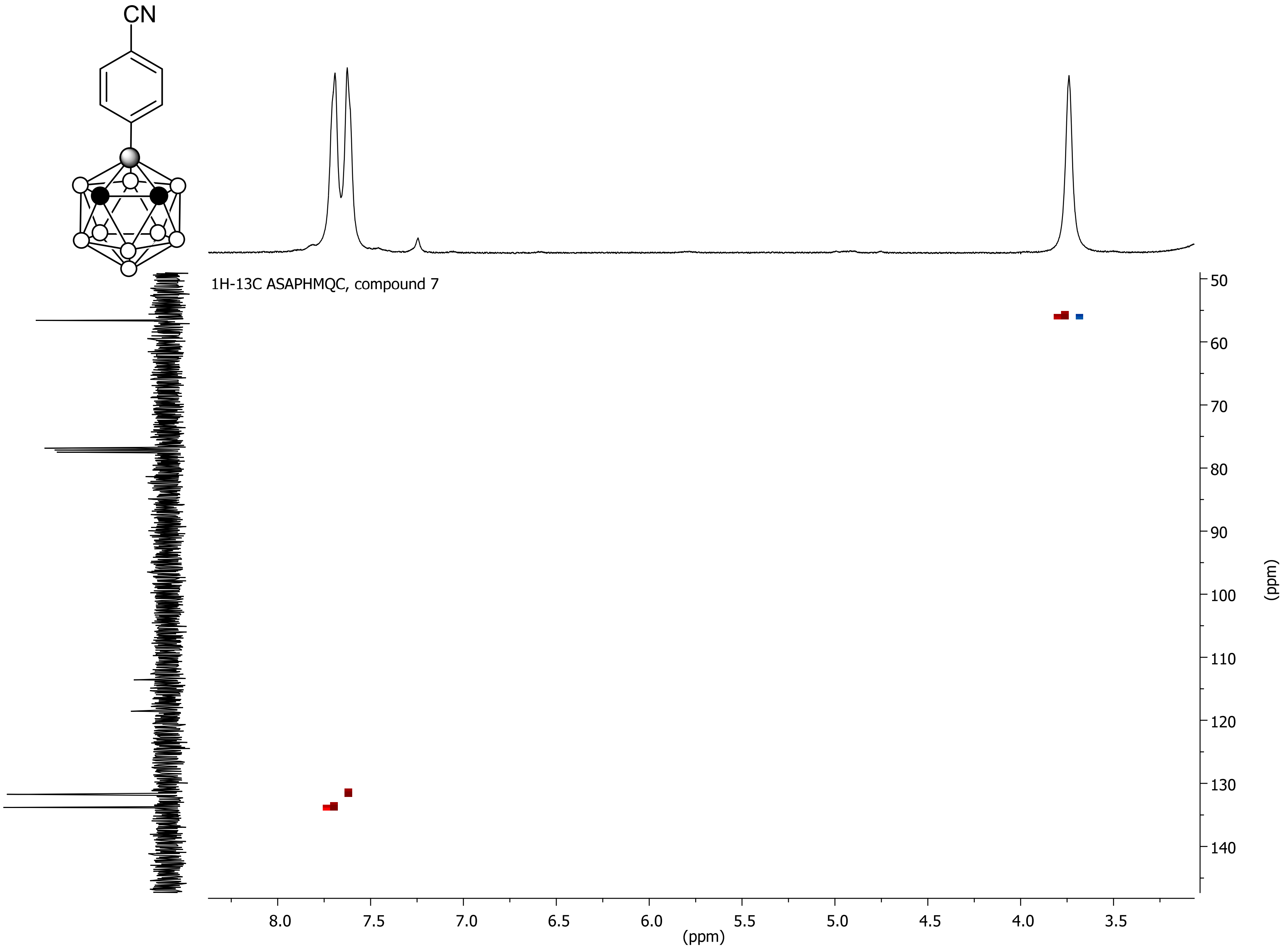
CN
1H-13C ASAPHMQC, compound 7
8.0
7.5
7.0
6.5
6.0
5.5
5.0
4.5
4.0
3.5
(ppm)
50
60
70
80
90
100
110
120
130
140
(ppm)

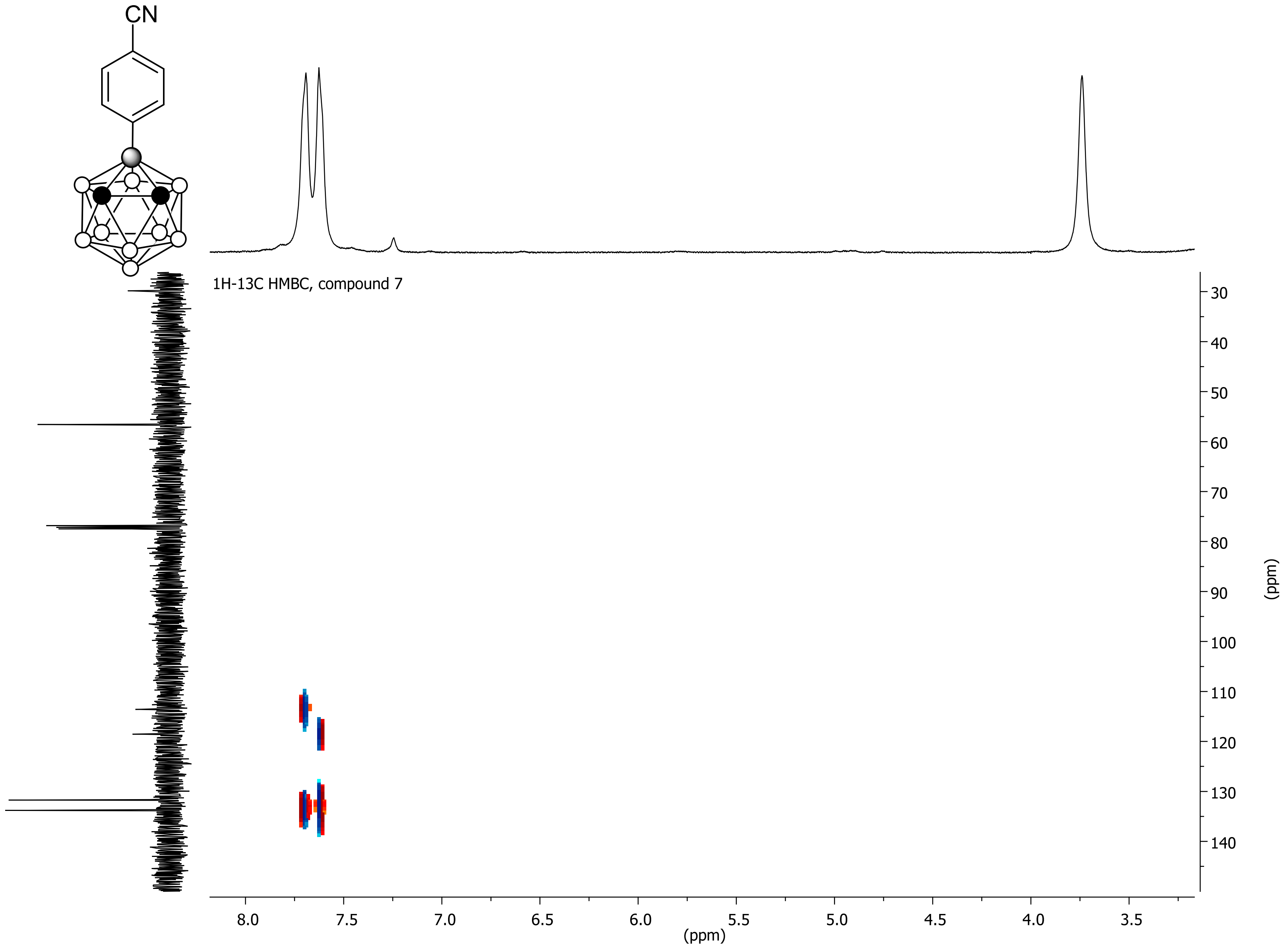
CN
1H-13C HMBC, compound 7
30
40
50
60
70
80
90
100
110
120
130
140
(ppm)
8.0
7.5
7.0
6.5
6.0
5.5
5.0
4.5
4.0
3.5
(ppm)

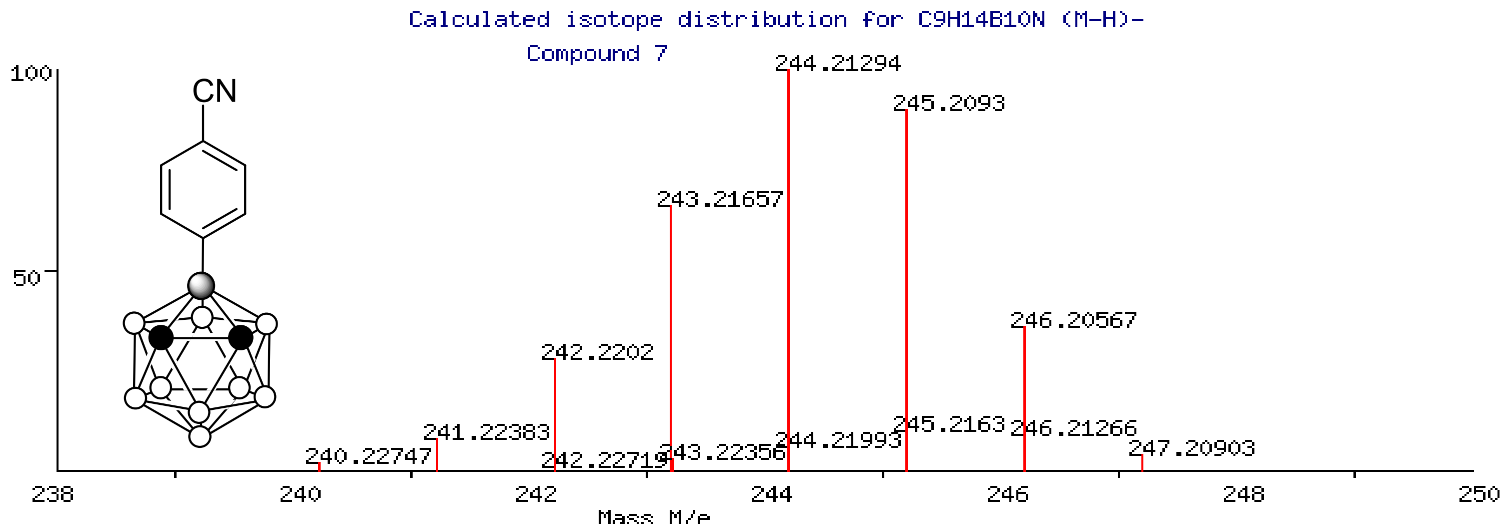
Calculated isotope distribution for C9H14B10N (M-H)-
Compound 7
CN
100
50
244.21294
245.2093
243.21657
246.20567
242.2202
241.22383
240.22747
242.22719
243.22356
244.21993
245.2163
246.21266
247.20903
238
240
242
244
246
248
250
Mass M/e

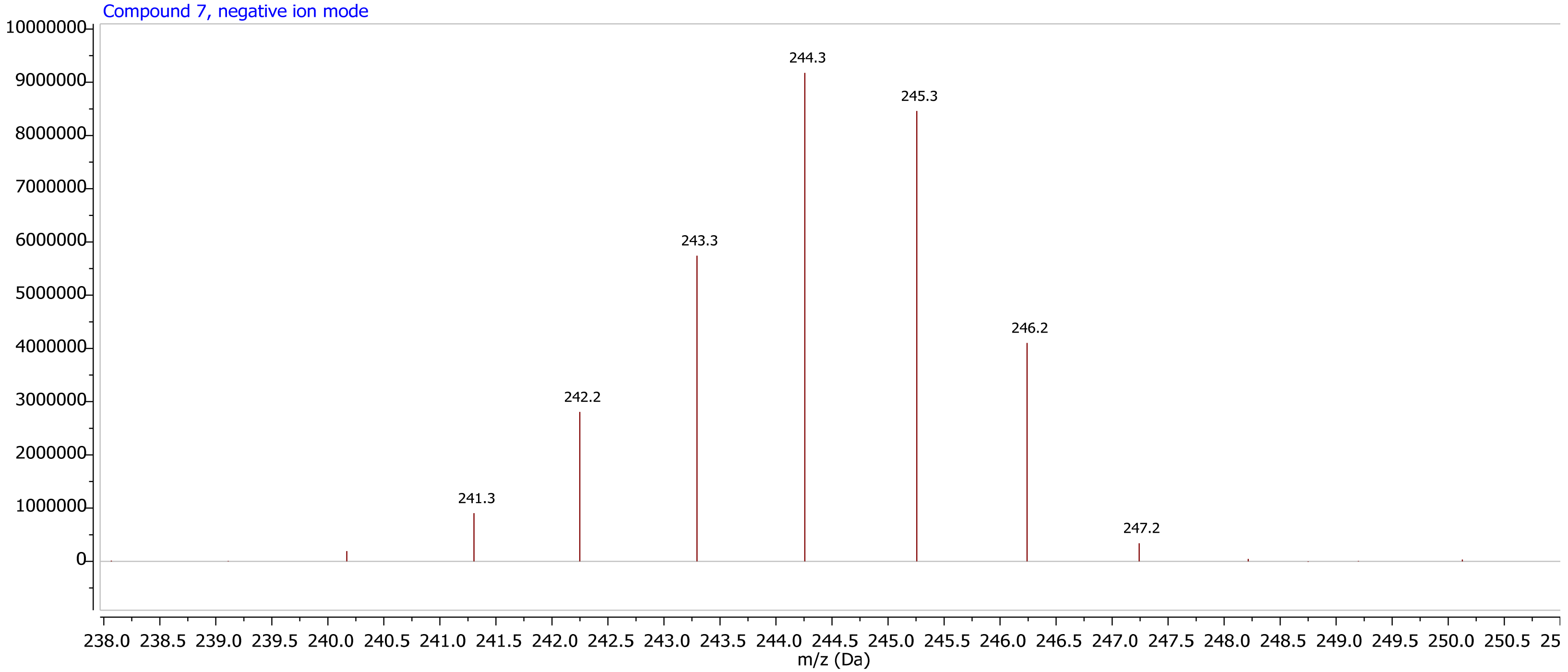
Compound 7, negative ion mode
10000000
9000000
8000000
7000000
6000000
5000000
4000000
3000000
2000000
1000000
0
244.3
245.3
243.3
246.2
242.2
241.3
247.2
238.0 238.5 239.0 239.5 240.0 240.5 241.0 241.5 242.0 242.5 243.0 243.5 244.0 244.5 245.0 245.5 246.0 246.5 247.0 247.5 248.0 248.5 249.0 249.5 250.0 250.5 25
m/z (Da)

1H, compound 8

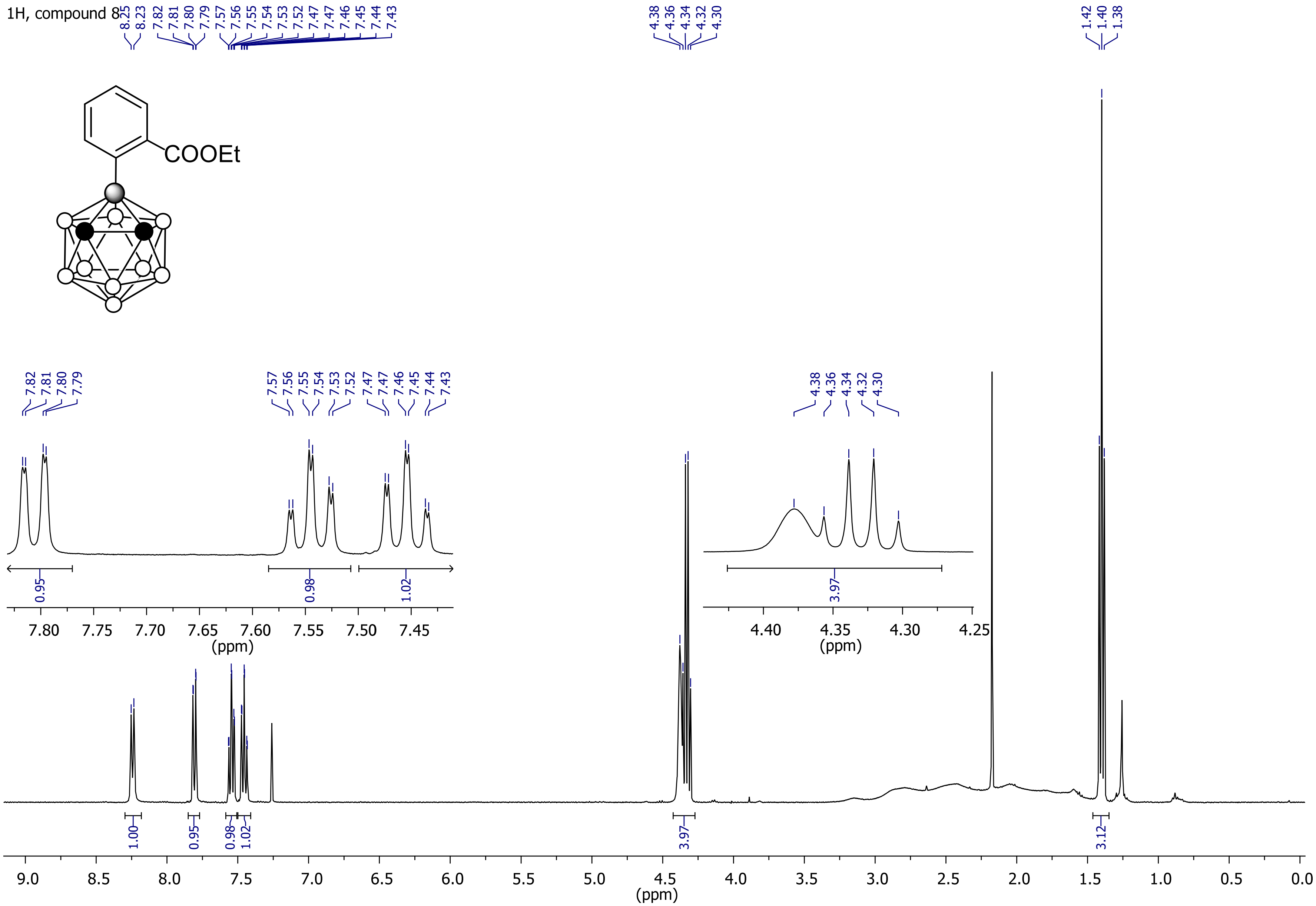

11B{1H}, compound 8

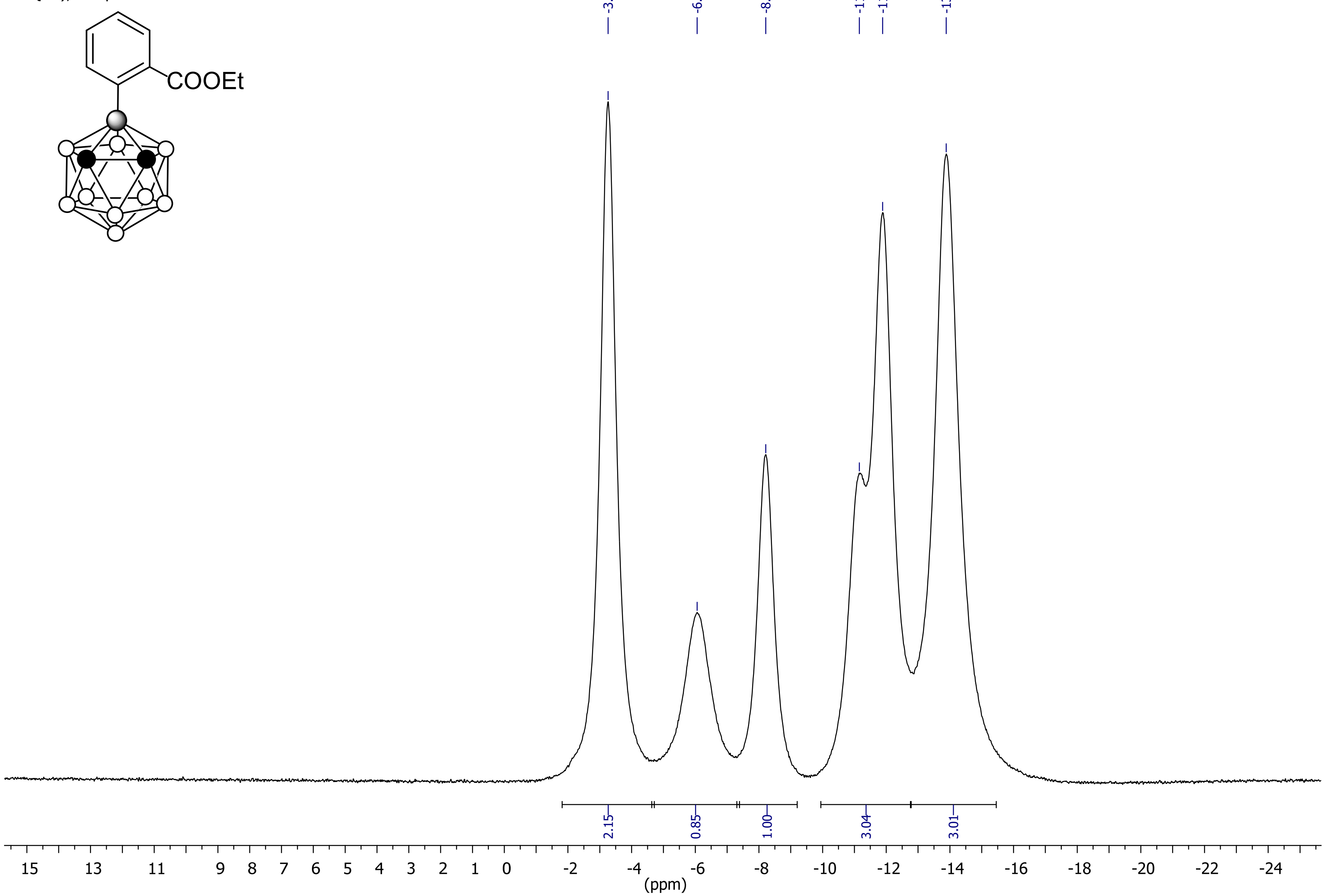

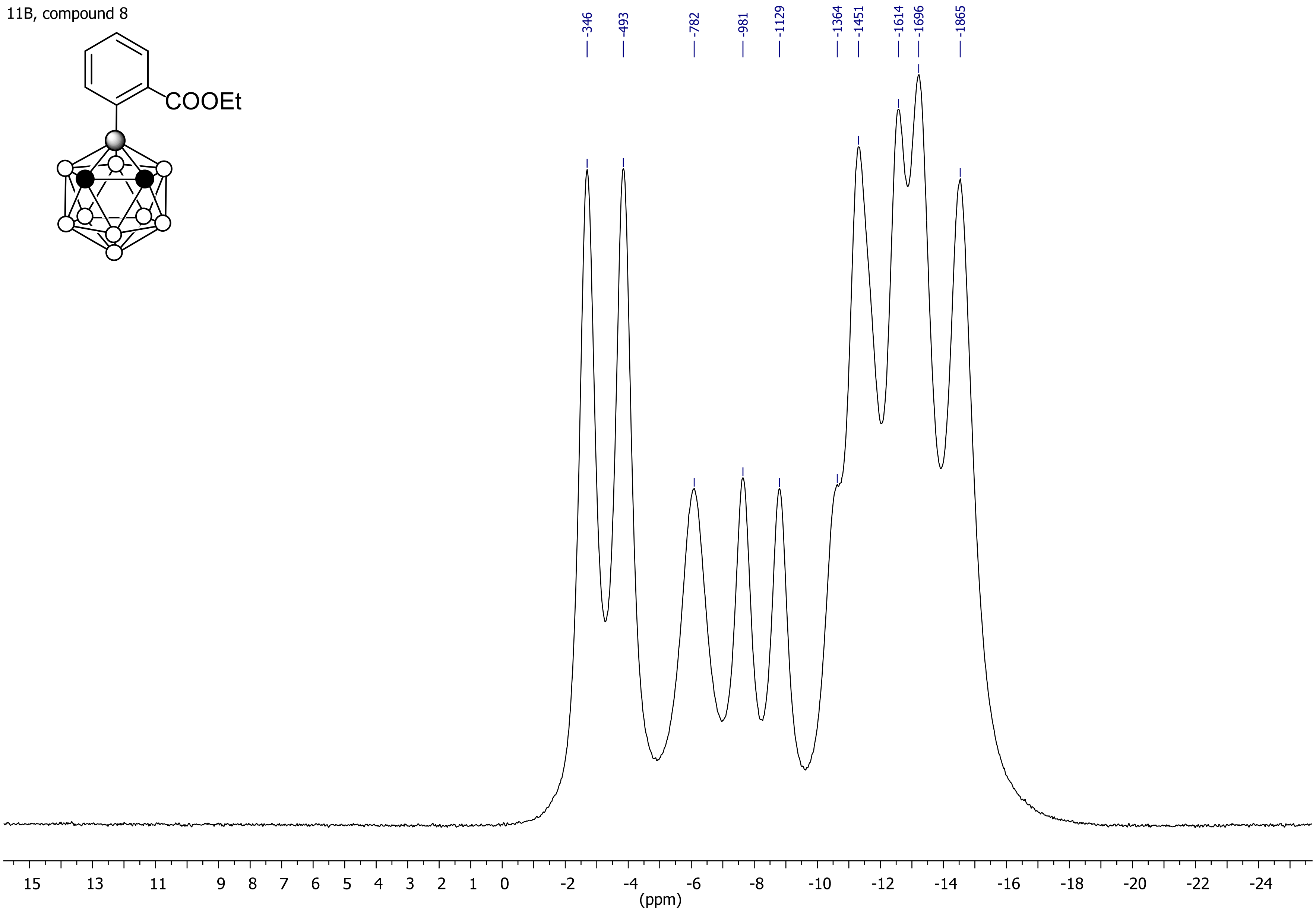
11B, compound 8
COOEt
-346
-493
-782
-981
-1129
-1364
-1451
-1614
-1696
-1865
15 13 11 9 8 7 6 5 4 3 2 1 0 -2 -4 -6 -8 -10 -12 -14 -16 -18 -20 -22 -24
(ppm)

13C{1H}, compound 8

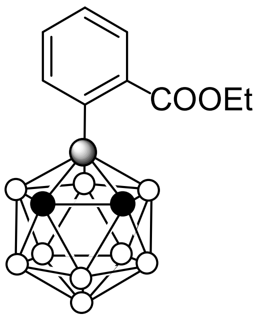

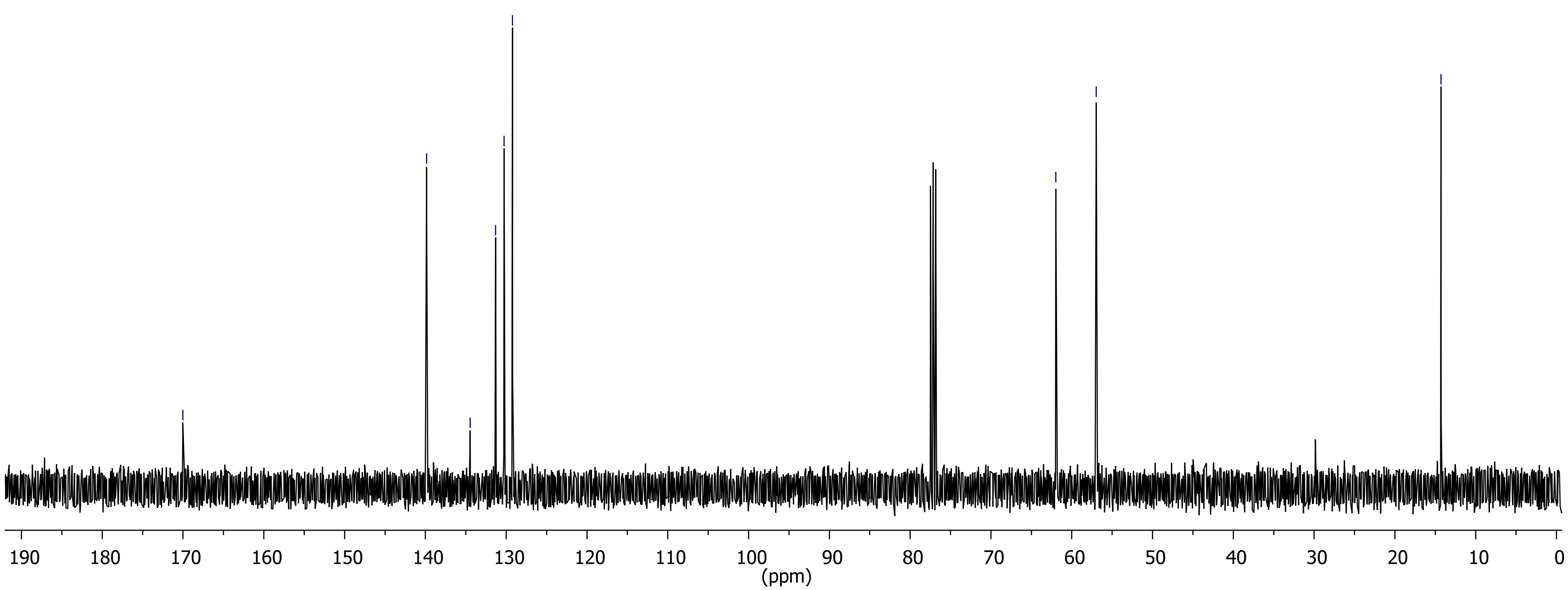

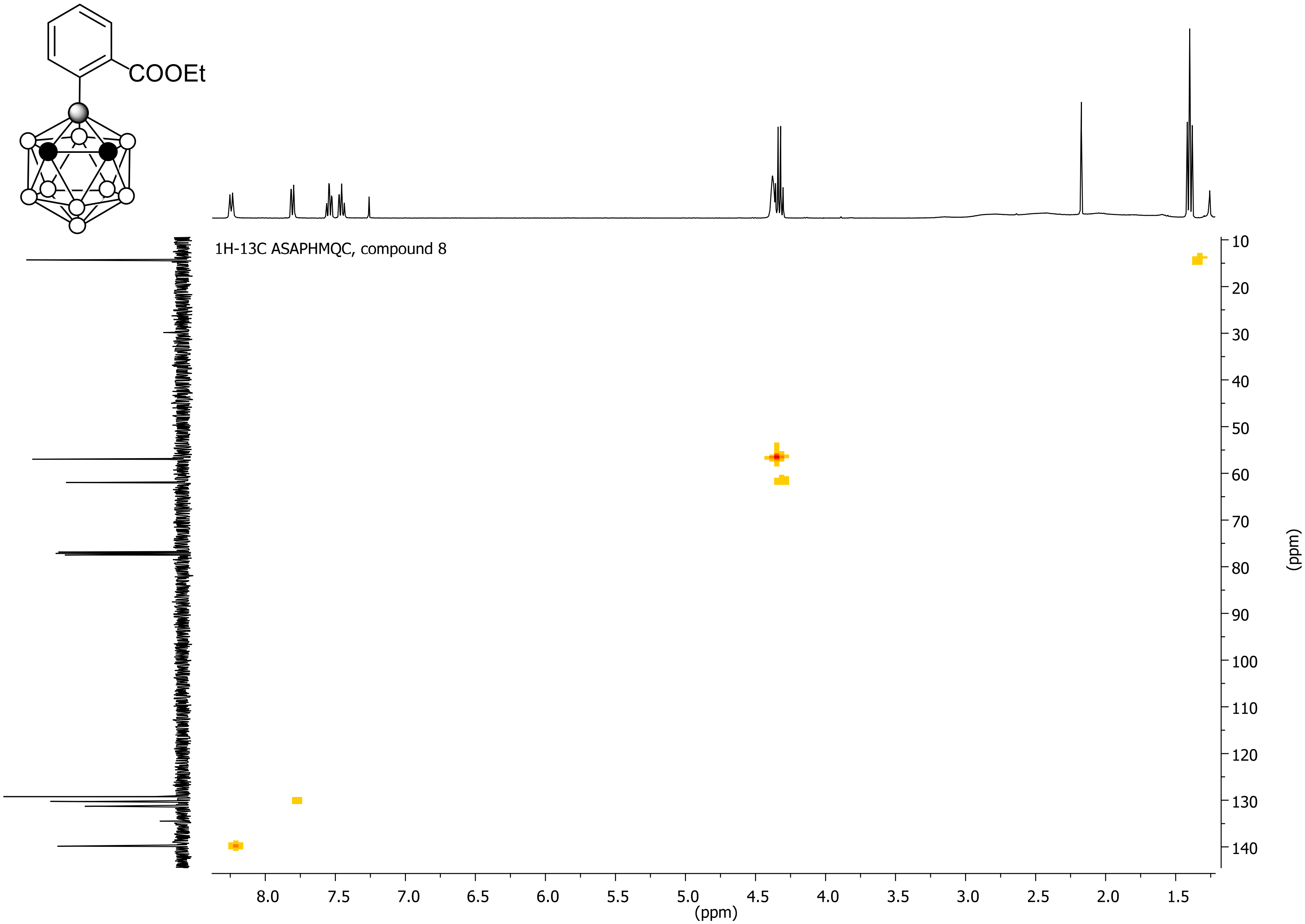
COOEt
1H-13C ASAPHMQC, compound 8
10
20
30
40
50
60
70
80
90
100
110
120
130
140
(ppm)
8.0
7.5
7.0
6.5
6.0
5.5
5.0
4.5
4.0
3.5
3.0
2.5
2.0
1.5
(ppm)

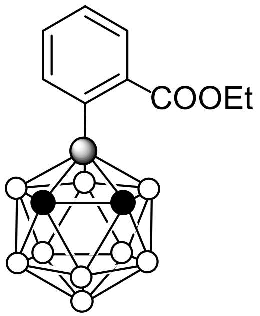
COOEt

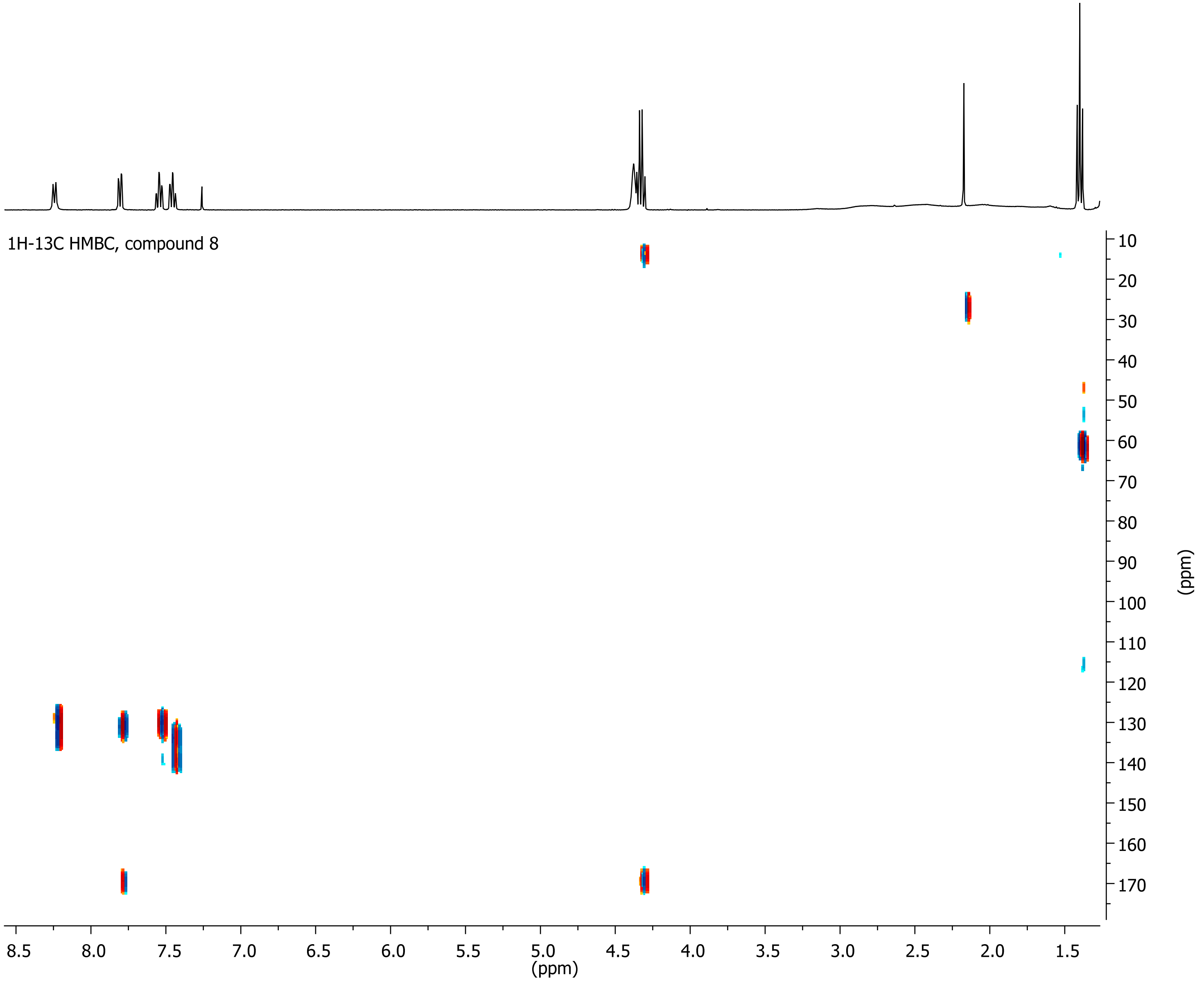
1H-13C HMBC, compound 8
8.5
8.0
7.5
7.0
6.5
6.0
5.5
5.0
(ppm)
4.5
4.0
3.5
3.0
2.5
2.0
1.5
10
20
30
40
50
60
70
80
90
100
110
120
130
140
150
160
170
(ppm)

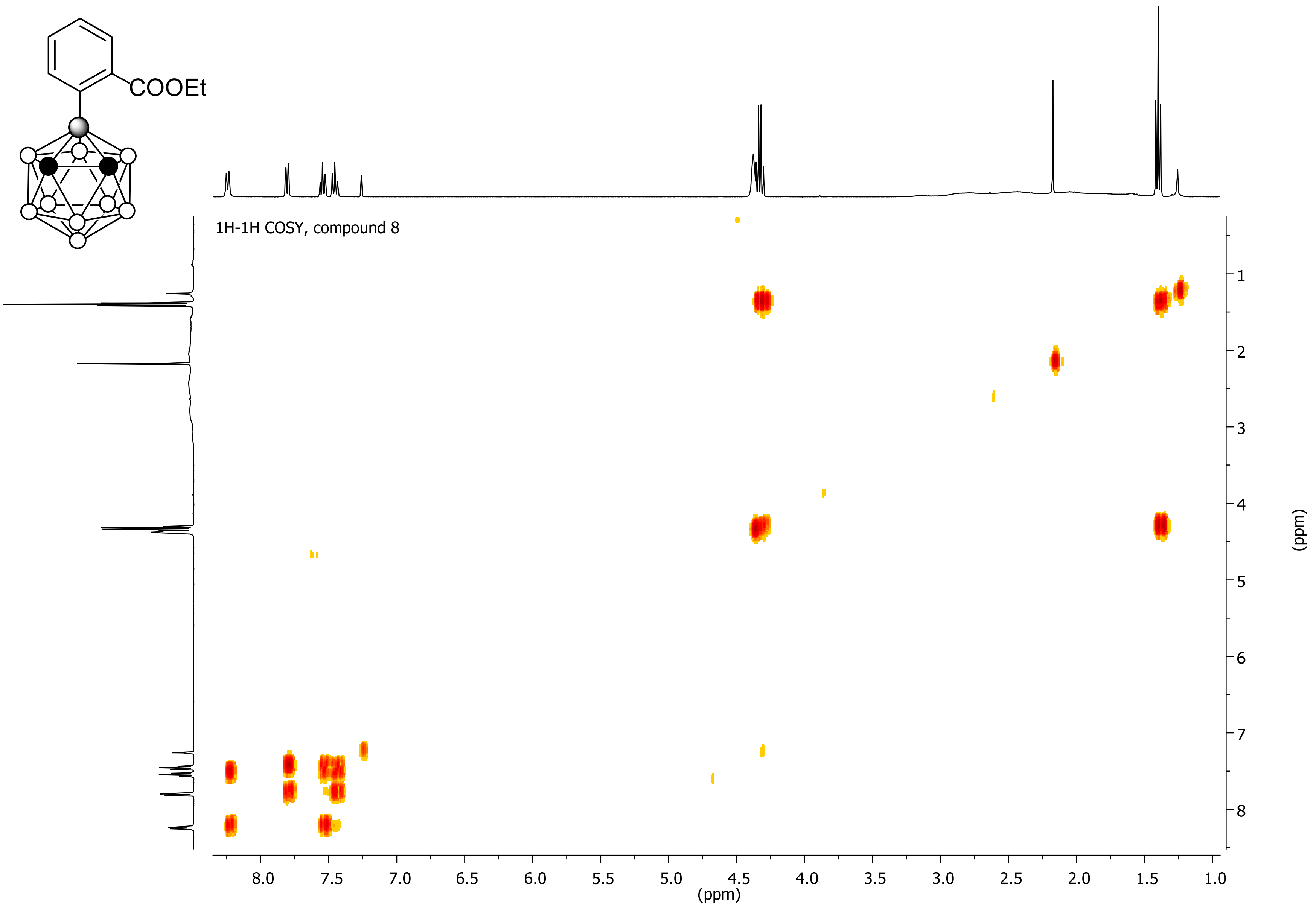

COOEt
1H-1H COSY, compound 8
8.0
7.5
7.0
6.5
6.0
5.5
5.0
4.5
4.0
3.5
3.0
2.5
2.0
1.5
1.0
(ppm)
1
2
3
4
5
6
7
8
(ppm)

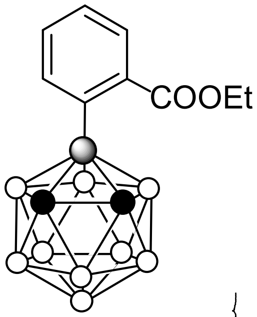
COOEt

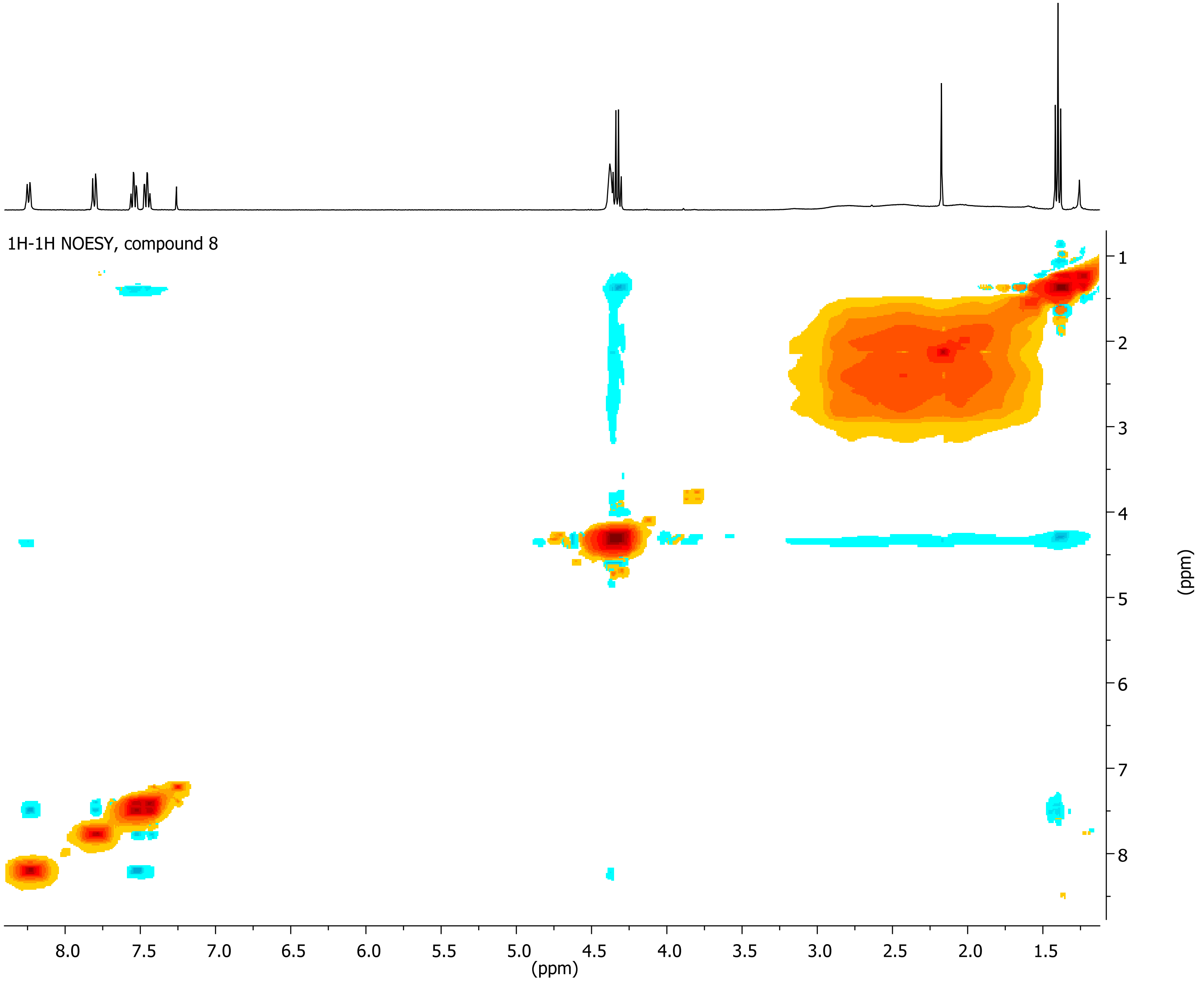
1H-1H NOESY, compound 8
8.0
7.5
7.0
6.5
6.0
5.5
5.0
4.5
4.0
3.5
3.0
2.5
2.0
1.5
(ppm)
1
2
3
4
5
6
7
8
(ppm)

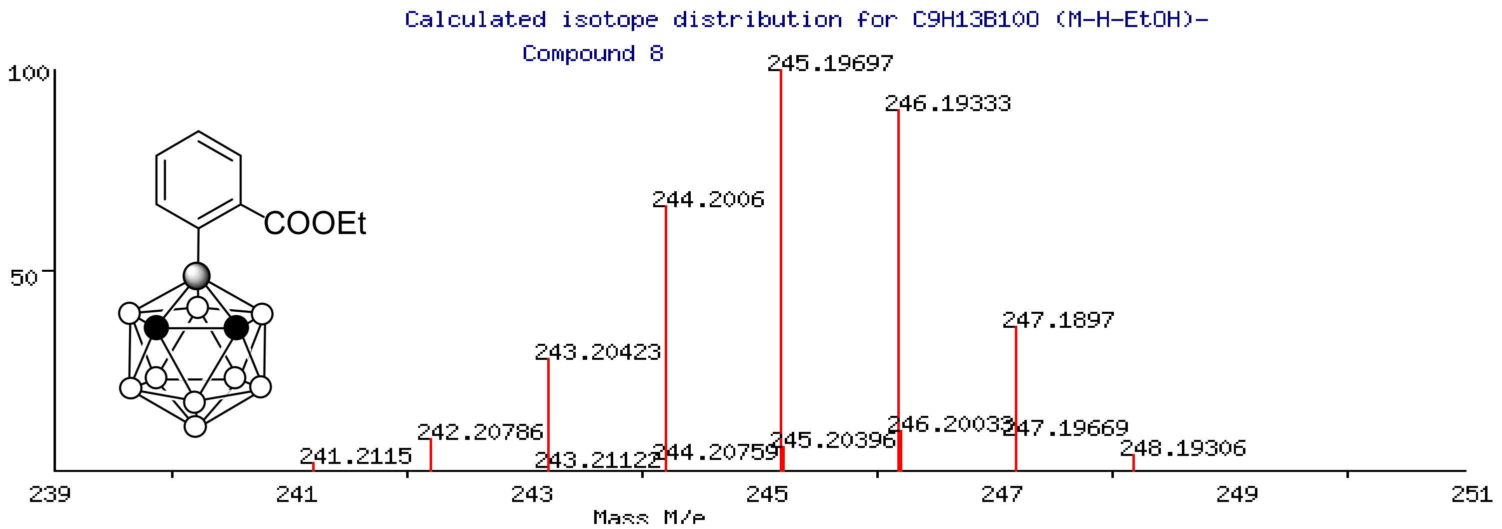
Calculated isotope distribution for C9H13B10O (M-H-EtOH)-
Compound 8
COOEt
245.19697
246.19333
244.2006
247.1897
243.20423
242.20786
241.2115
243.21122
244.20759
245.20396
246.20033
247.19669
248.19306
100
50
239
241
243
245
247
249
251
Mass M/e

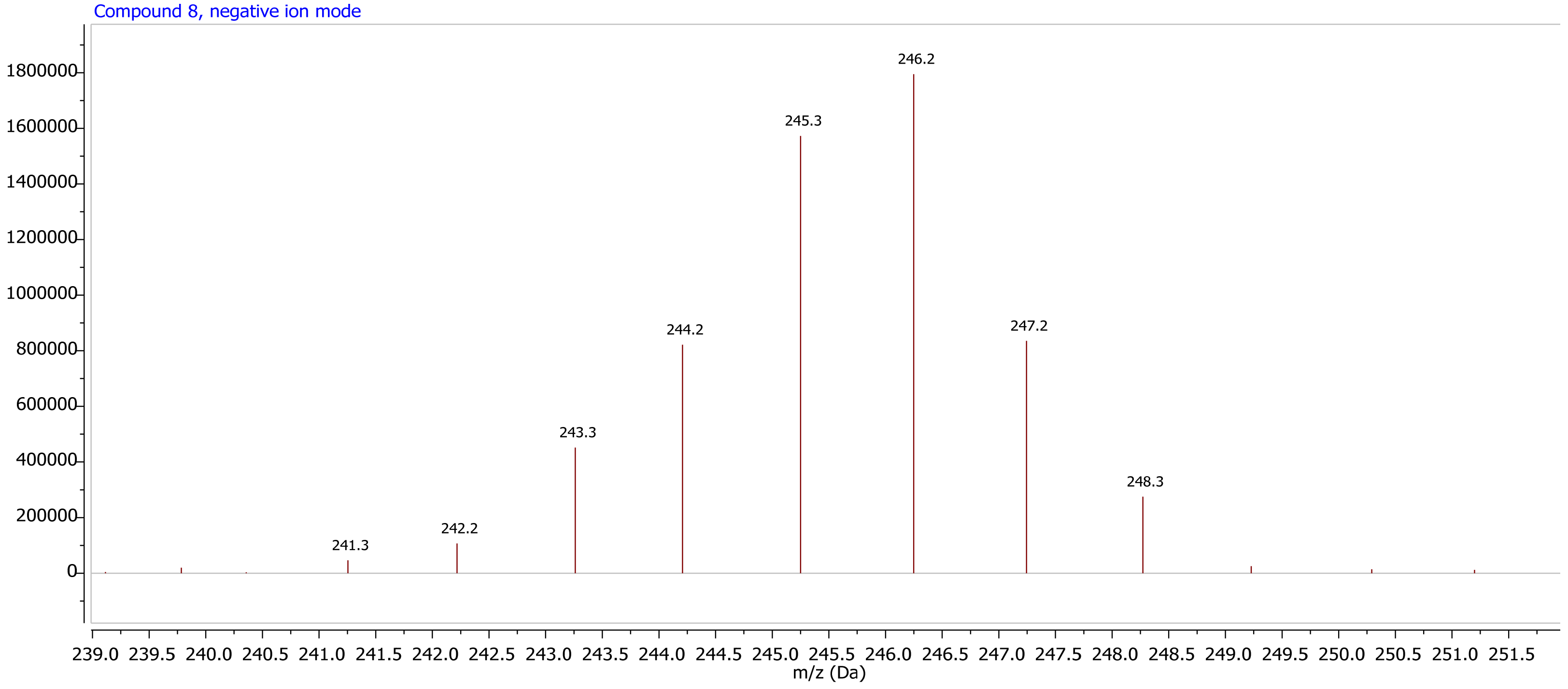
Compound 8, negative ion mode
246.2
245.3
244.2
247.2
243.3
248.3
242.2
241.3
1800000
1600000
1400000
1200000
1000000
800000
600000
400000
200000
0
239.0 239.5 240.0 240.5 241.0 241.5 242.0 242.5 243.0 243.5 244.0 244.5 245.0 245.5 246.0 246.5 247.0 247.5 248.0 248.5 249.0 249.5 250.0 250.5 251.0 251.5
m/z (Da)

1H, compound 9

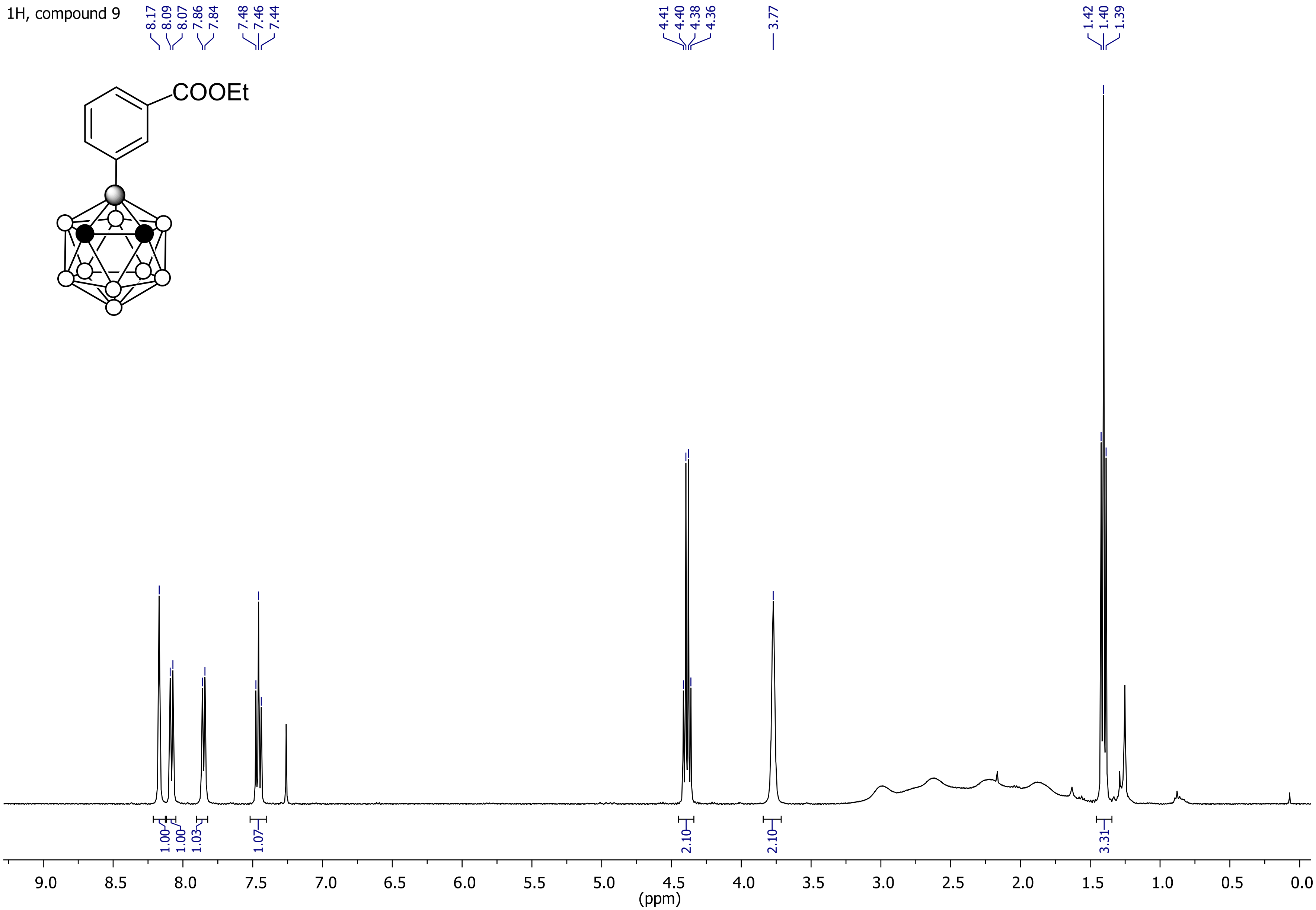

11B{1H}, compound 9

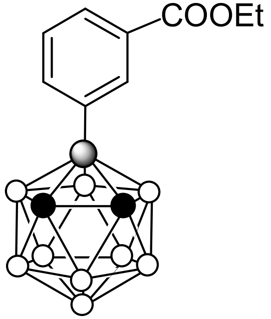

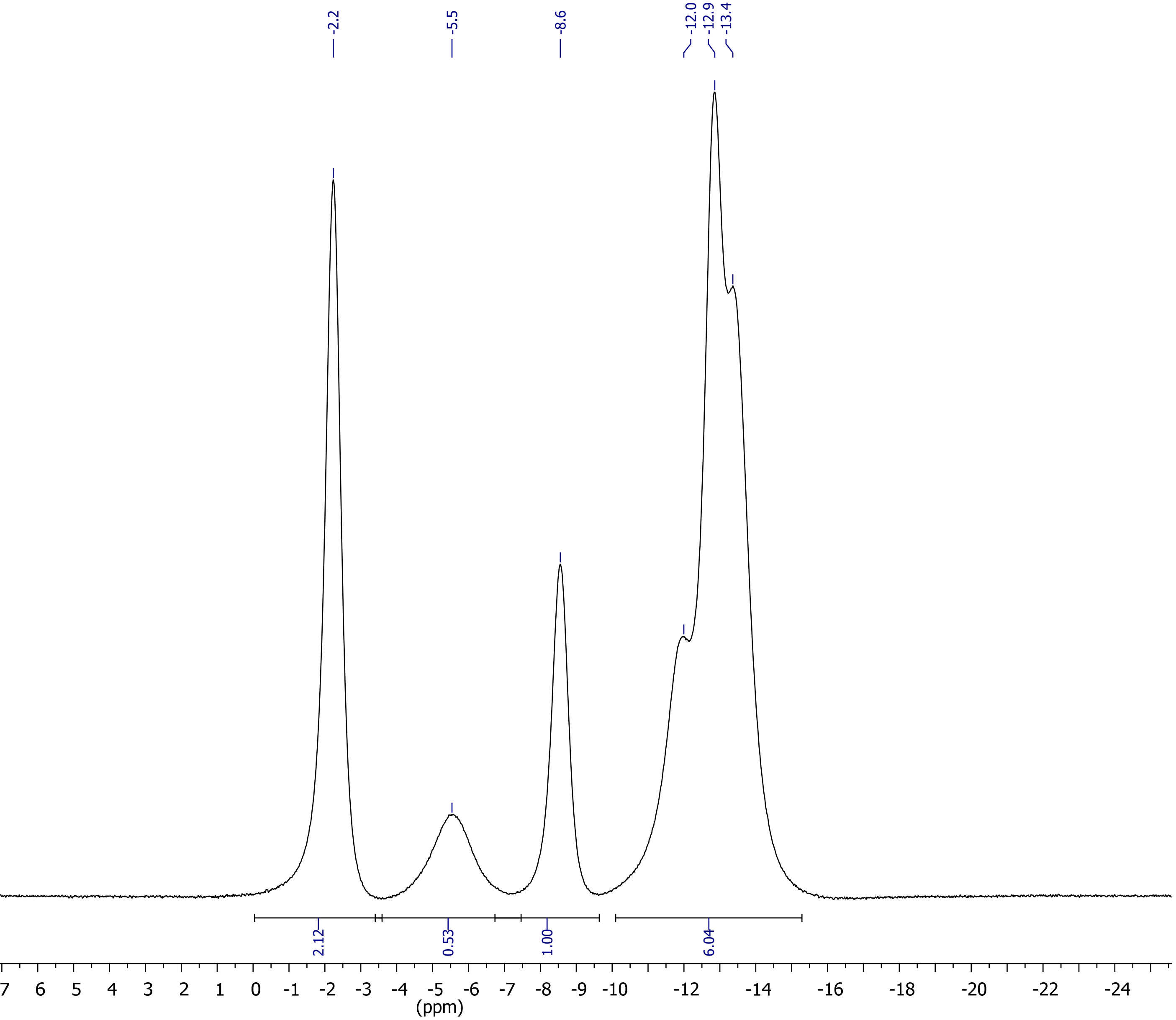

11B, compound 9

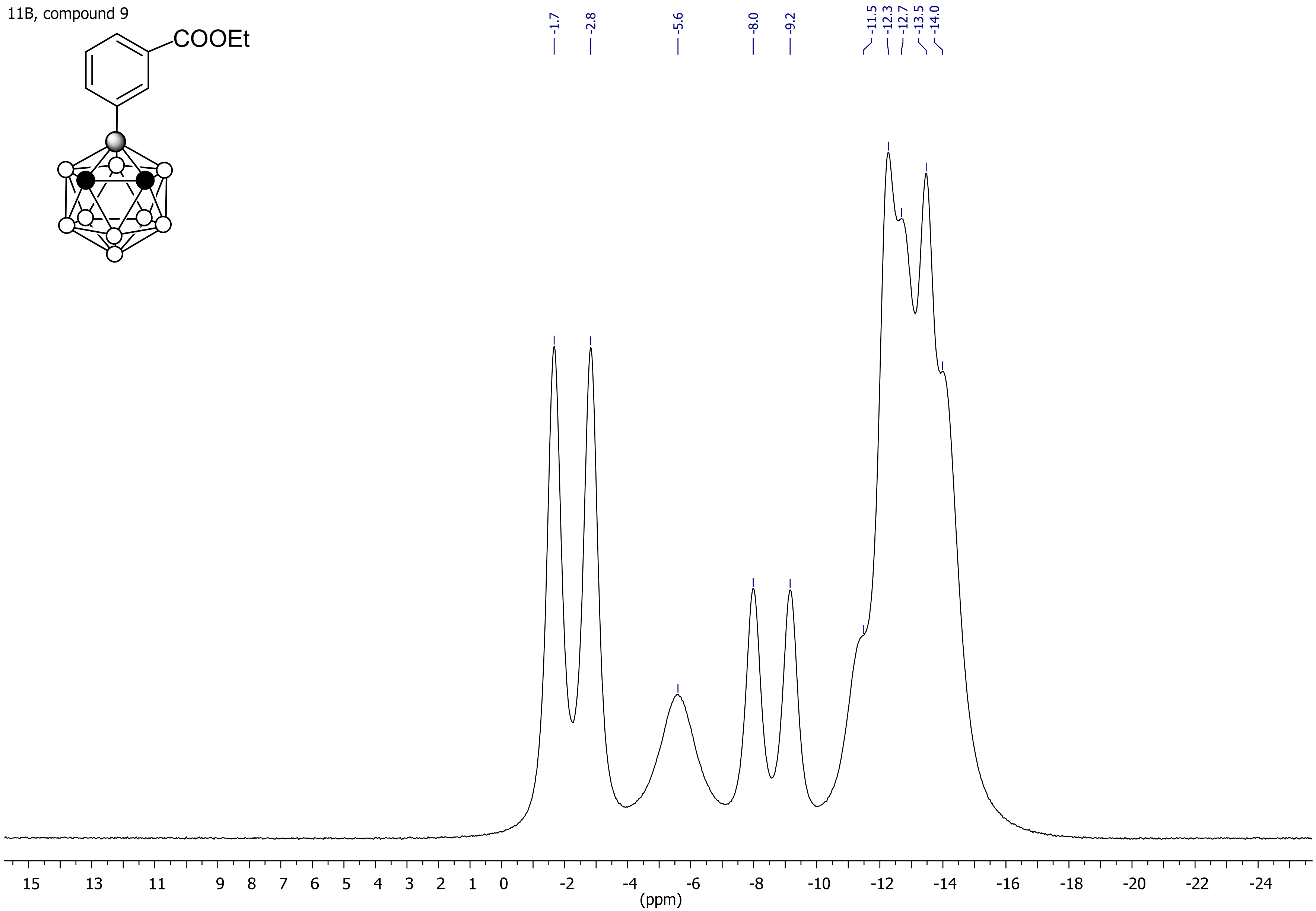

13C{1H}, compound 9

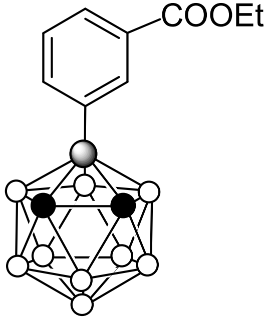

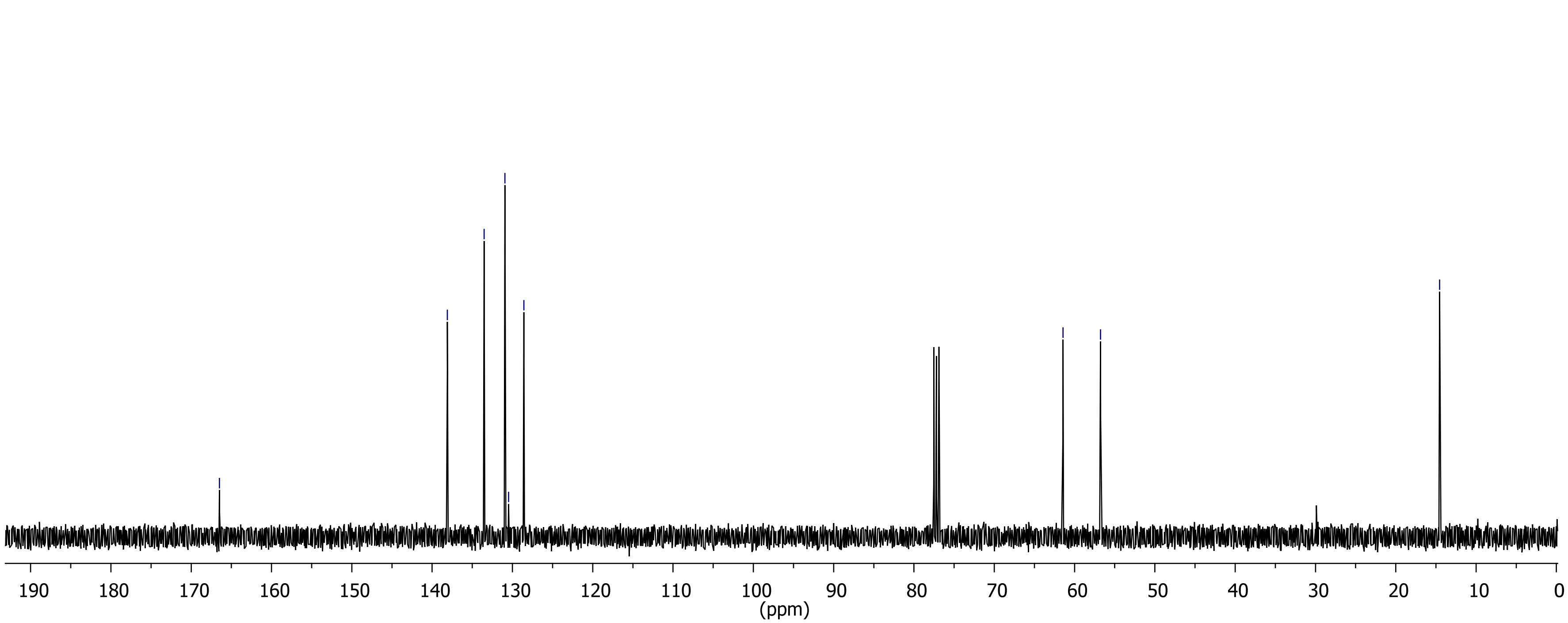

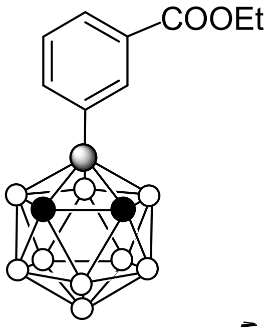
COOEt

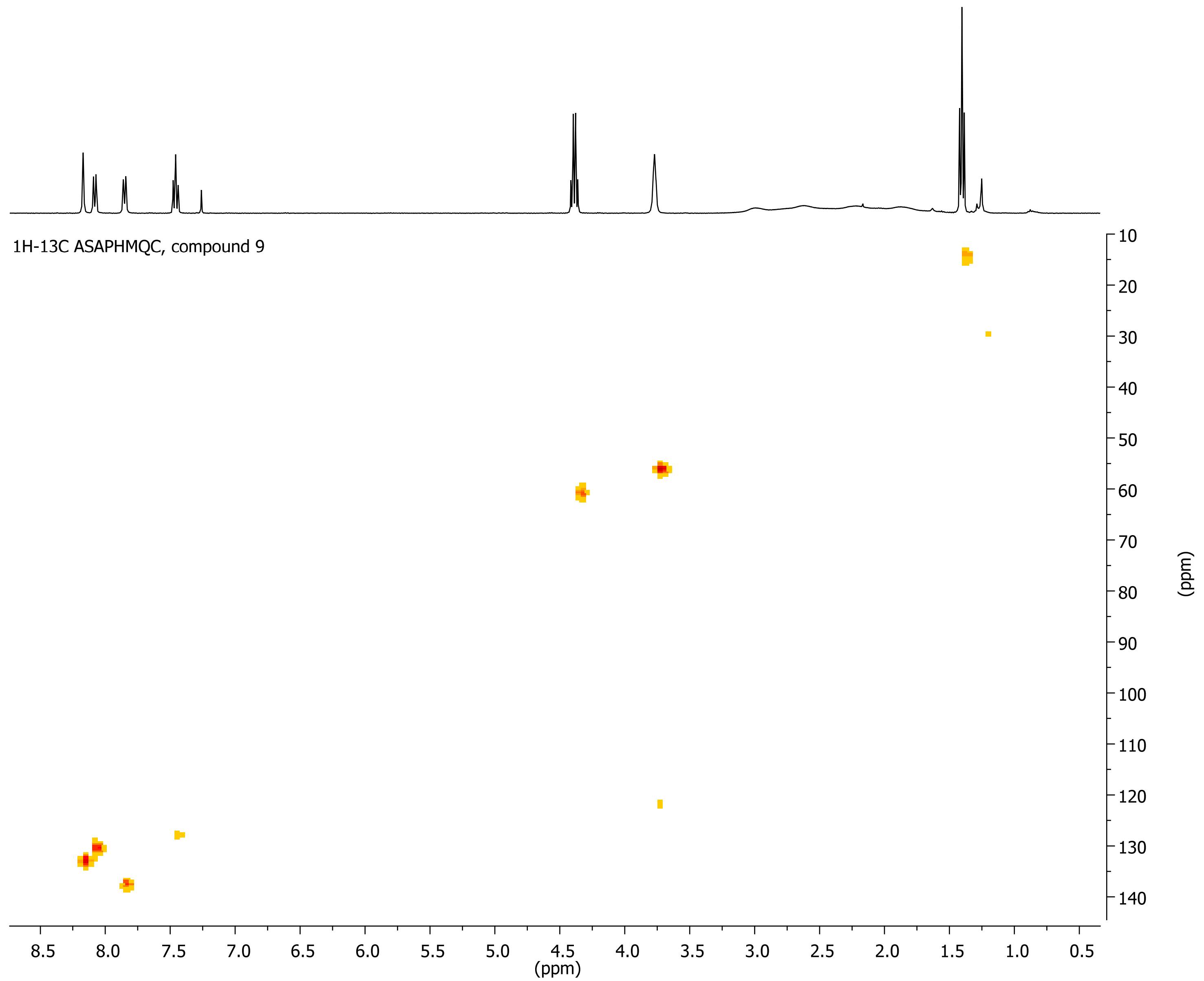
1H-13C ASAPHMQC, compound 9
8.5
8.0
7.5
7.0
6.5
6.0
5.5
5.0
4.5
4.0
3.5
3.0
2.5
2.0
1.5
1.0
0.5
(ppm)
10
20
30
40
50
60
70
80
90
100
110
120
130
140
(ppm)

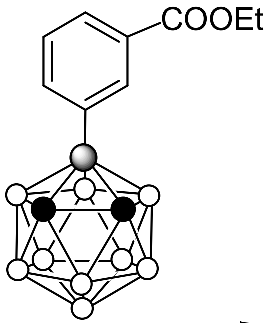
COOEt

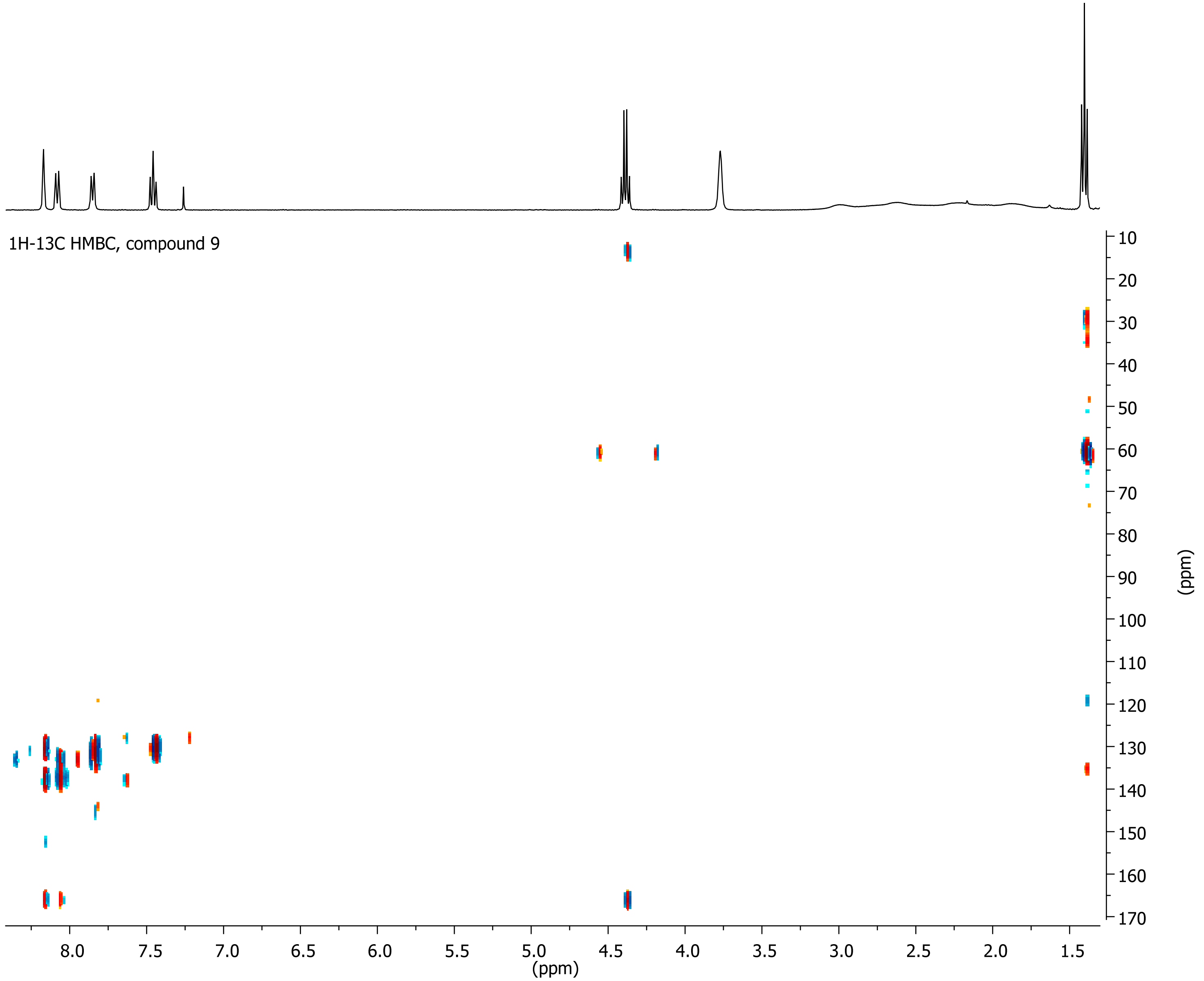
1H-13C HMBC, compound 9
(ppm)
(ppm)

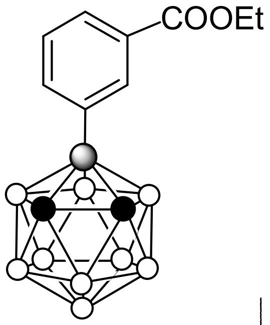
COOEt

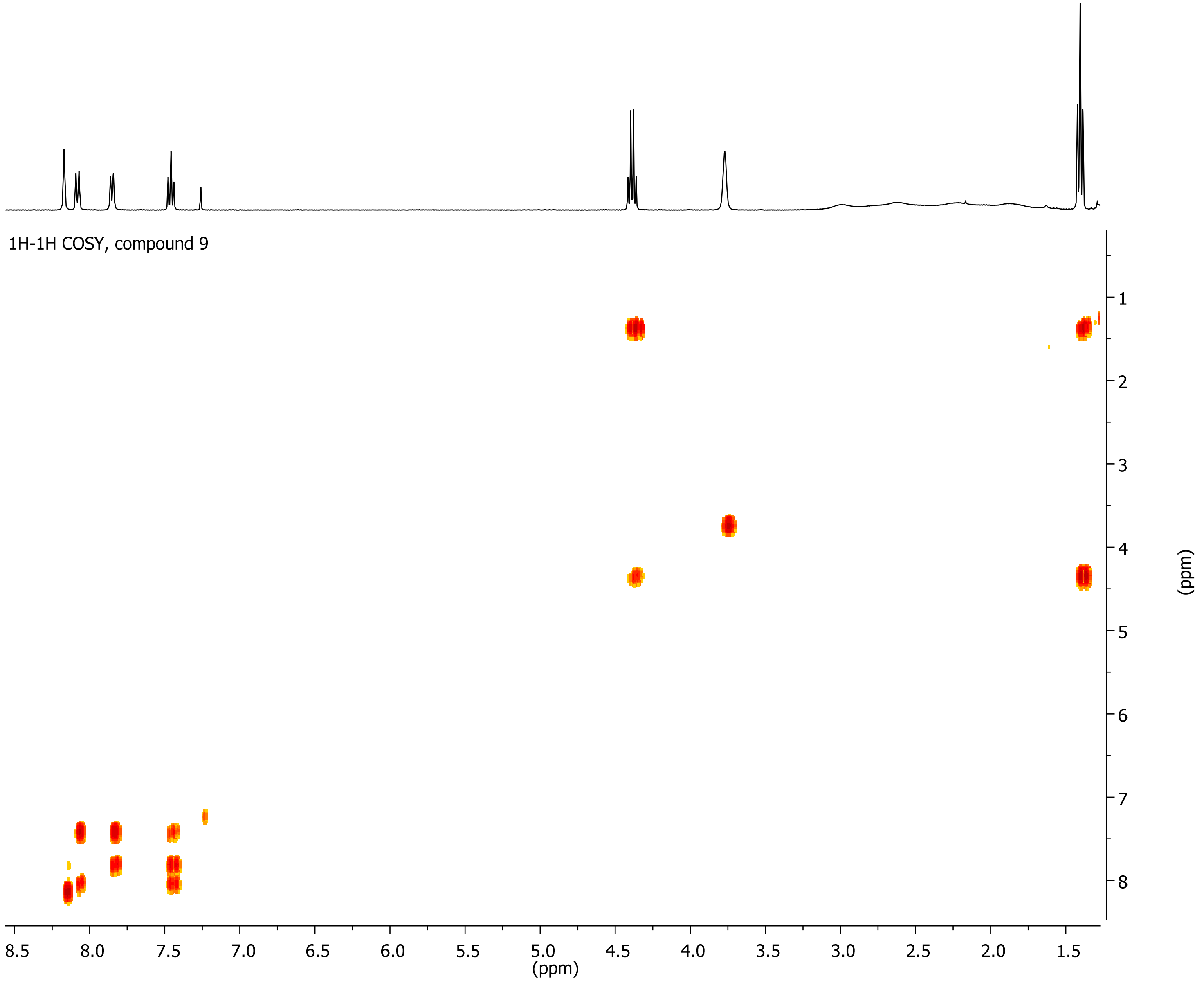
1H-1H COSY, compound 9
8.5
8.0
7.5
7.0
6.5
6.0
5.5
5.0
4.5
4.0
3.5
3.0
2.5
2.0
1.5
(ppm)
1
2
3
4
5
6
7
8
(ppm)

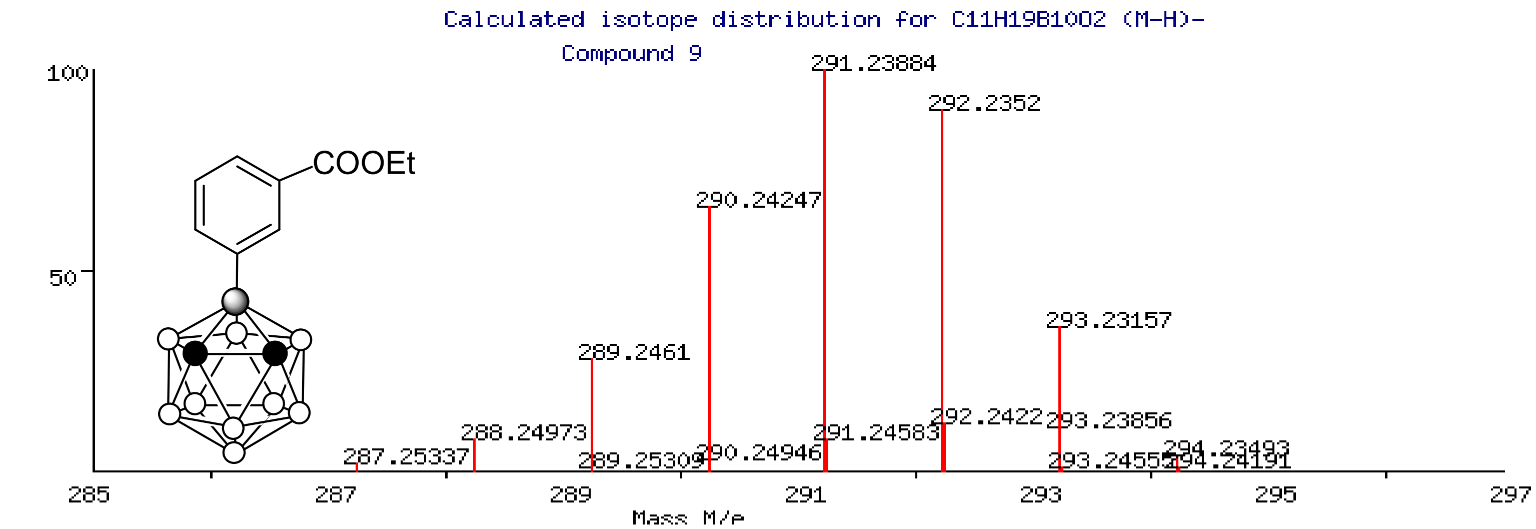
Calculated isotope distribution for C11H19B10O2 (M-H)-
Compound 9
COOEt
100
50
291.23884
292.2352
290.24247
293.23157
289.2461
288.24973
287.25337
289.25309
290.24946
291.24583
292.2422
293.23856
293.2455
294.23493
294.24191
285
287
289
291
293
295
297
Mass M/e

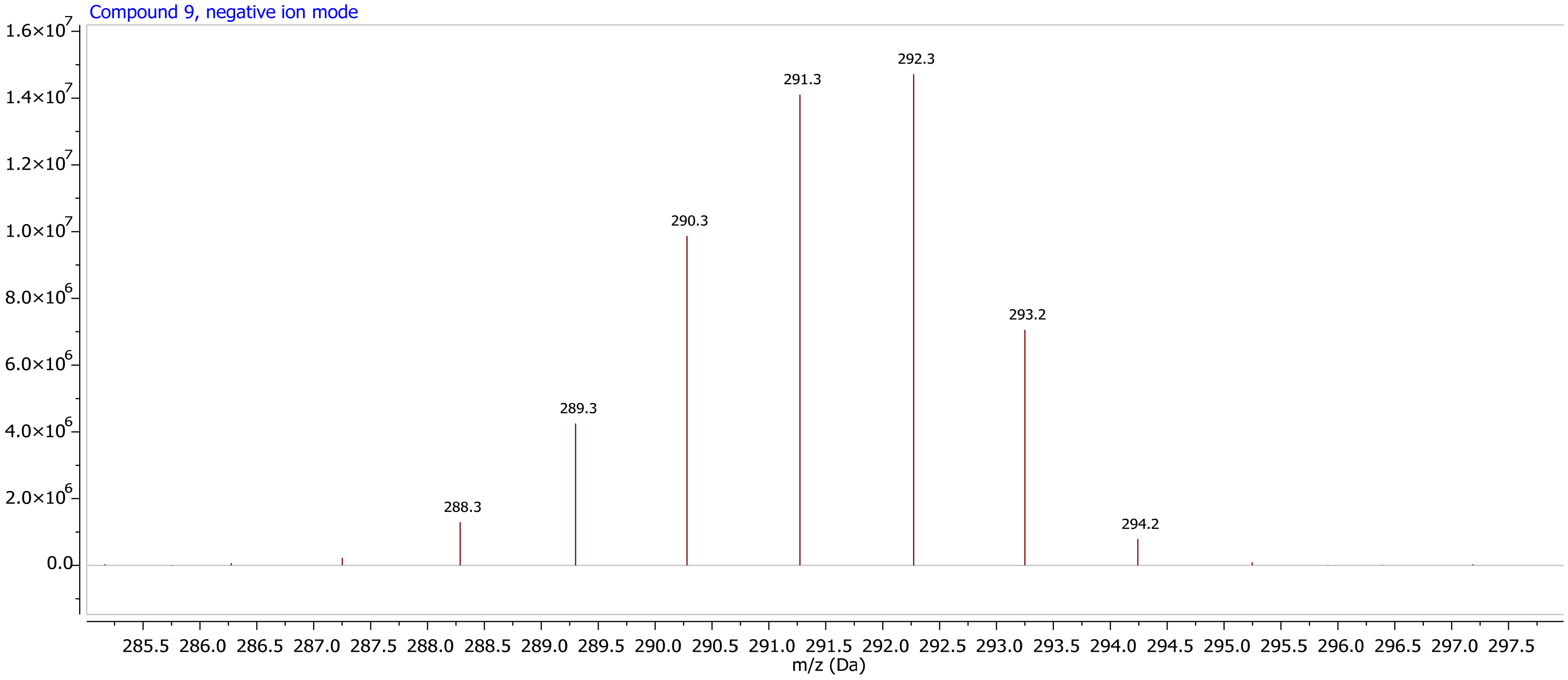
Compound 9, negative ion mode
1.6×10^7
1.4×10^7
1.2×10^7
1.0×10^7
8.0×10^6
6.0×10^6
4.0×10^6
2.0×10^6
0.0
291.3
292.3
290.3
293.2
289.3
288.3
294.2
285.5 286.0 286.5 287.0 287.5 288.0 288.5 289.0 289.5 290.0 290.5 291.0 291.5 292.0 292.5 293.0 293.5 294.0 294.5 295.0 295.5 296.0 296.5 297.0 297.5
m/z (Da)

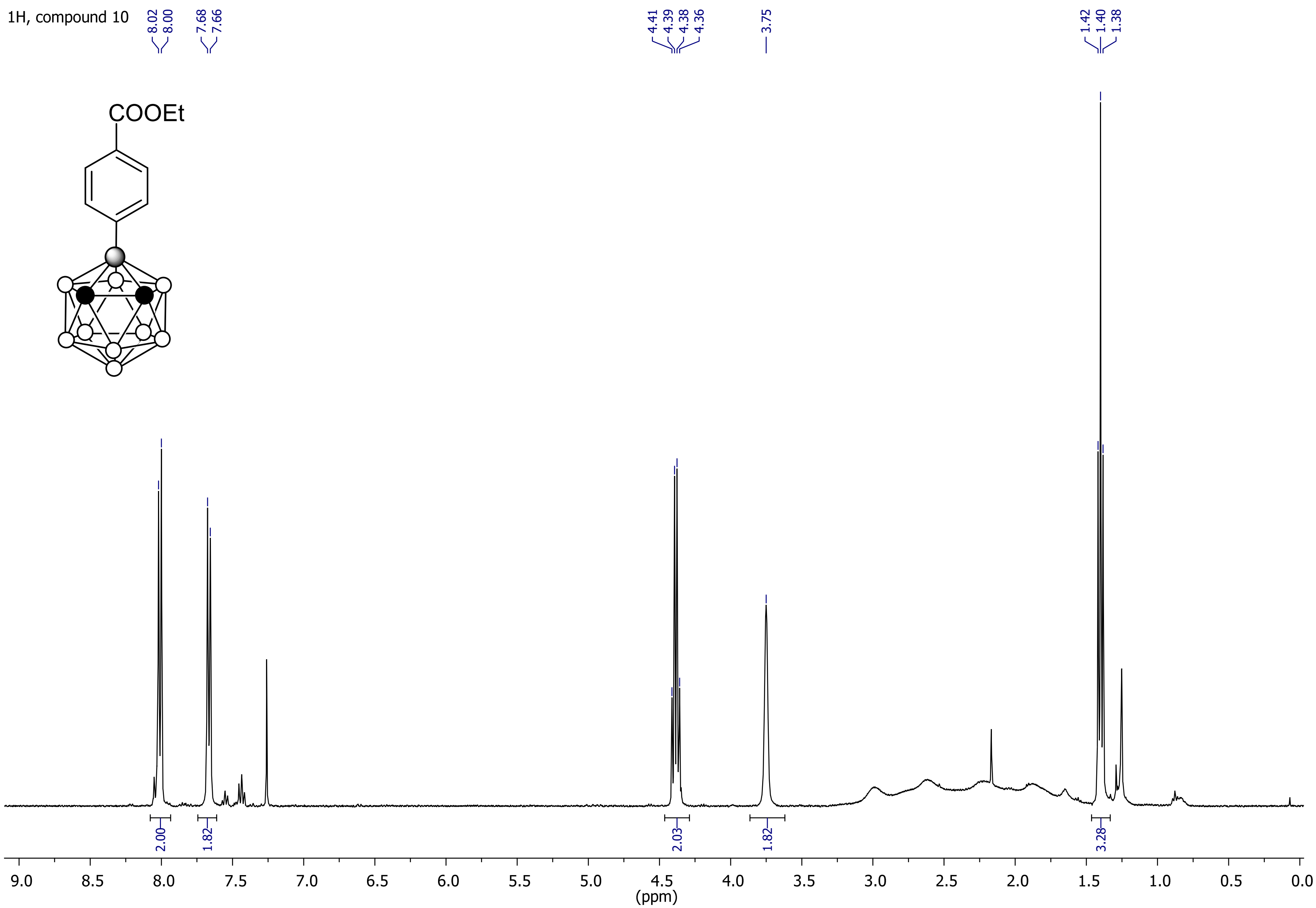
1H, compound 10
COOEt
8.02
8.00
7.68
7.66
4.41
4.39
4.38
4.36
3.75
1.42
1.40
1.38
2.00
1.82
2.03
1.82
3.28
9.0
8.5
8.0
7.5
7.0
6.5
6.0
5.5
5.0
4.5
4.0
3.5
3.0
2.5
2.0
1.5
1.0
0.5
0.0
(ppm)

11B{1H}, compound 10

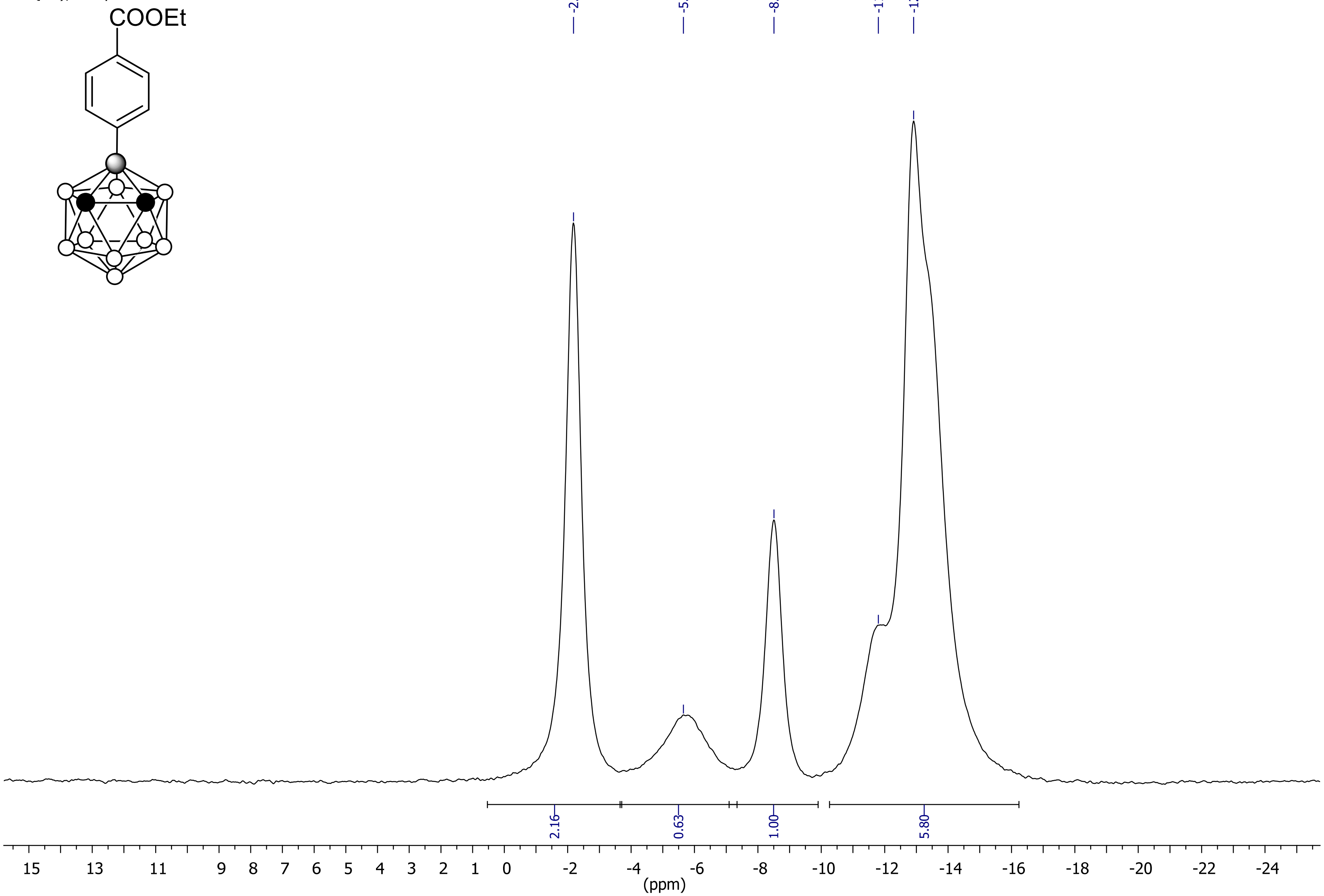

11B, compound 10

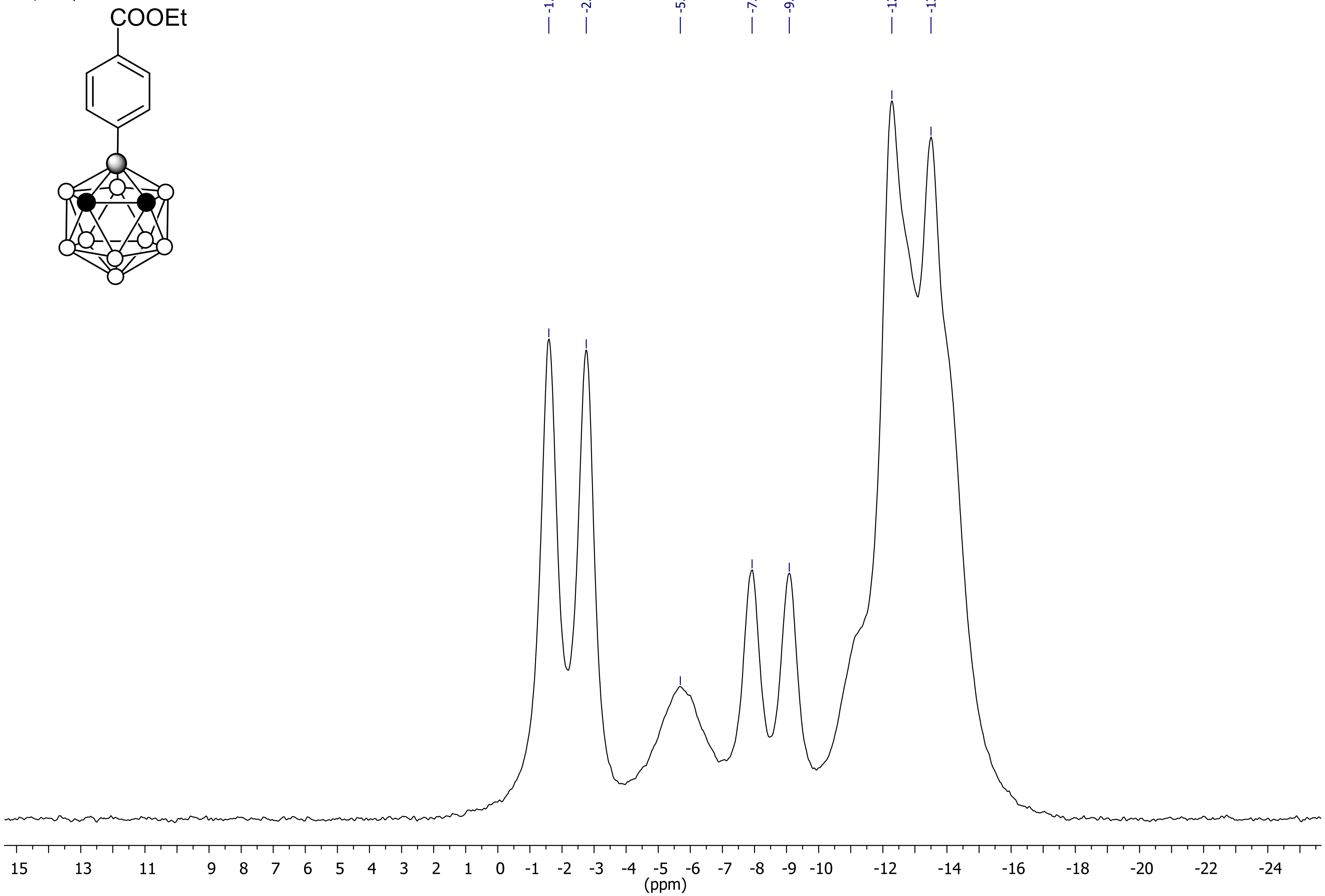

13C{1H}, compound 10

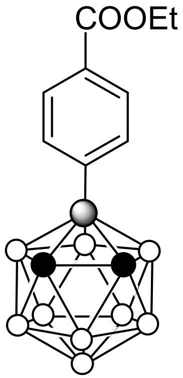

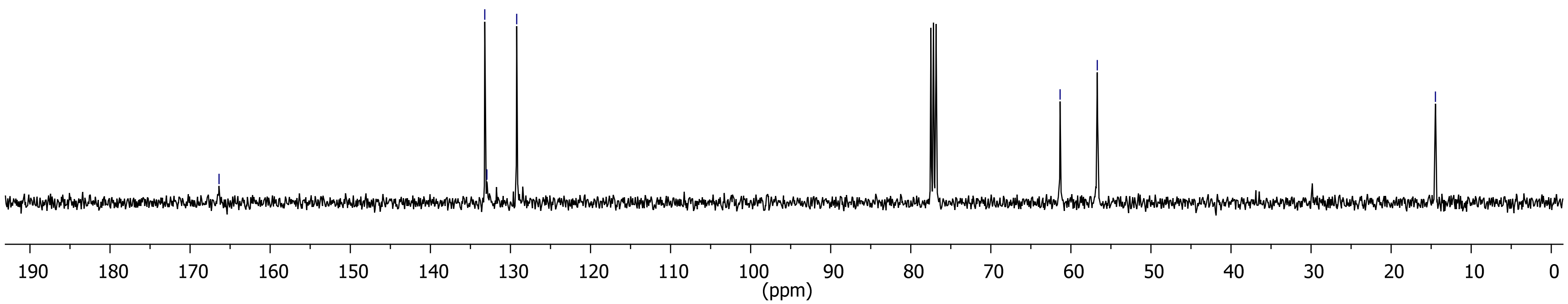

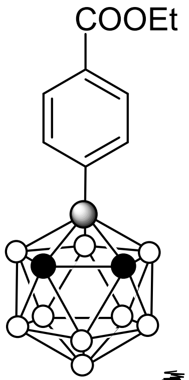
COOEt

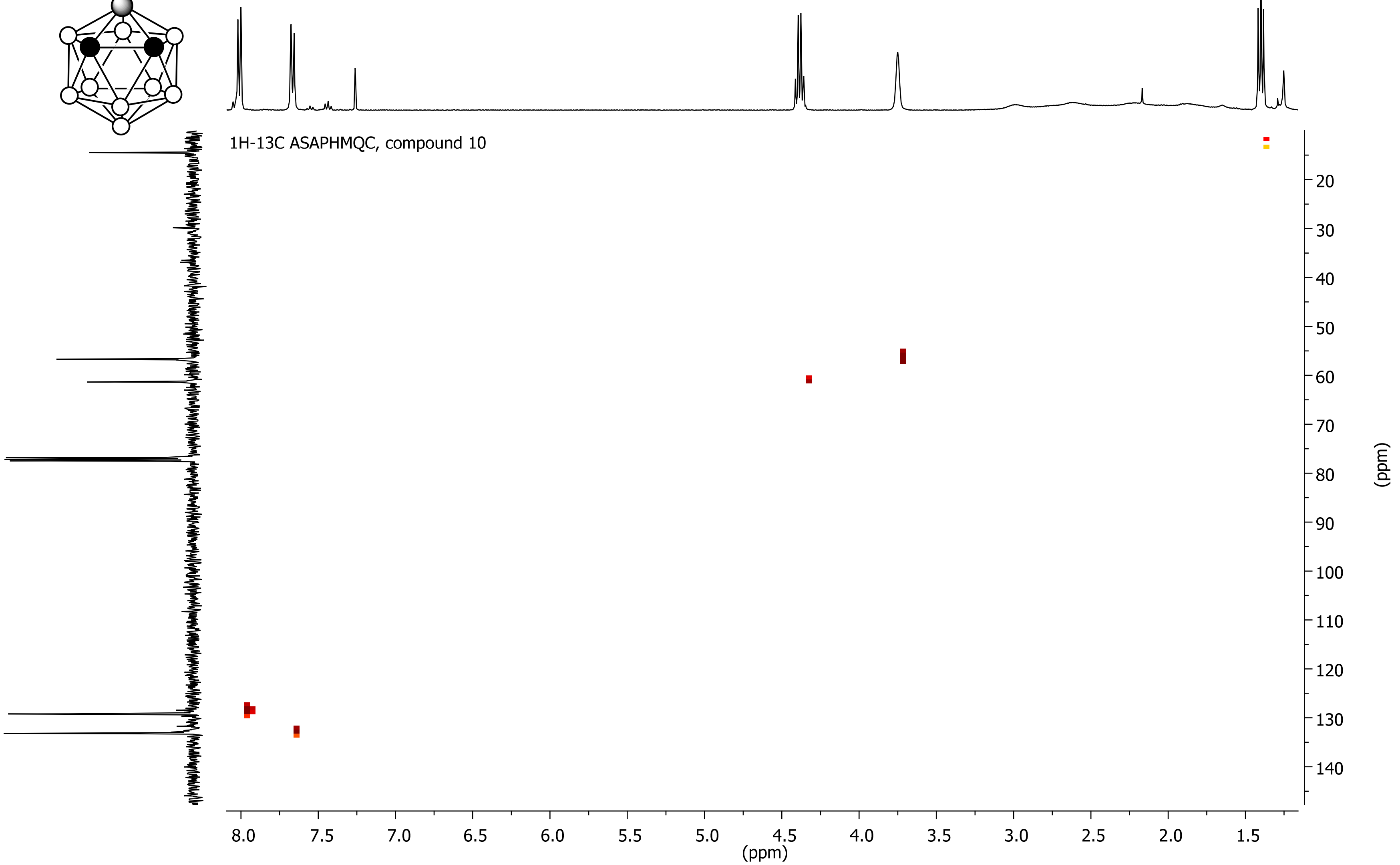
1H-13C ASAPHMQC, compound 10
(ppm)
(ppm)

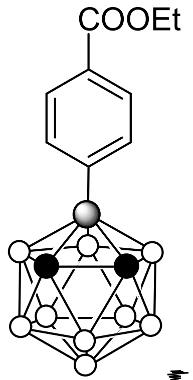
COOEt

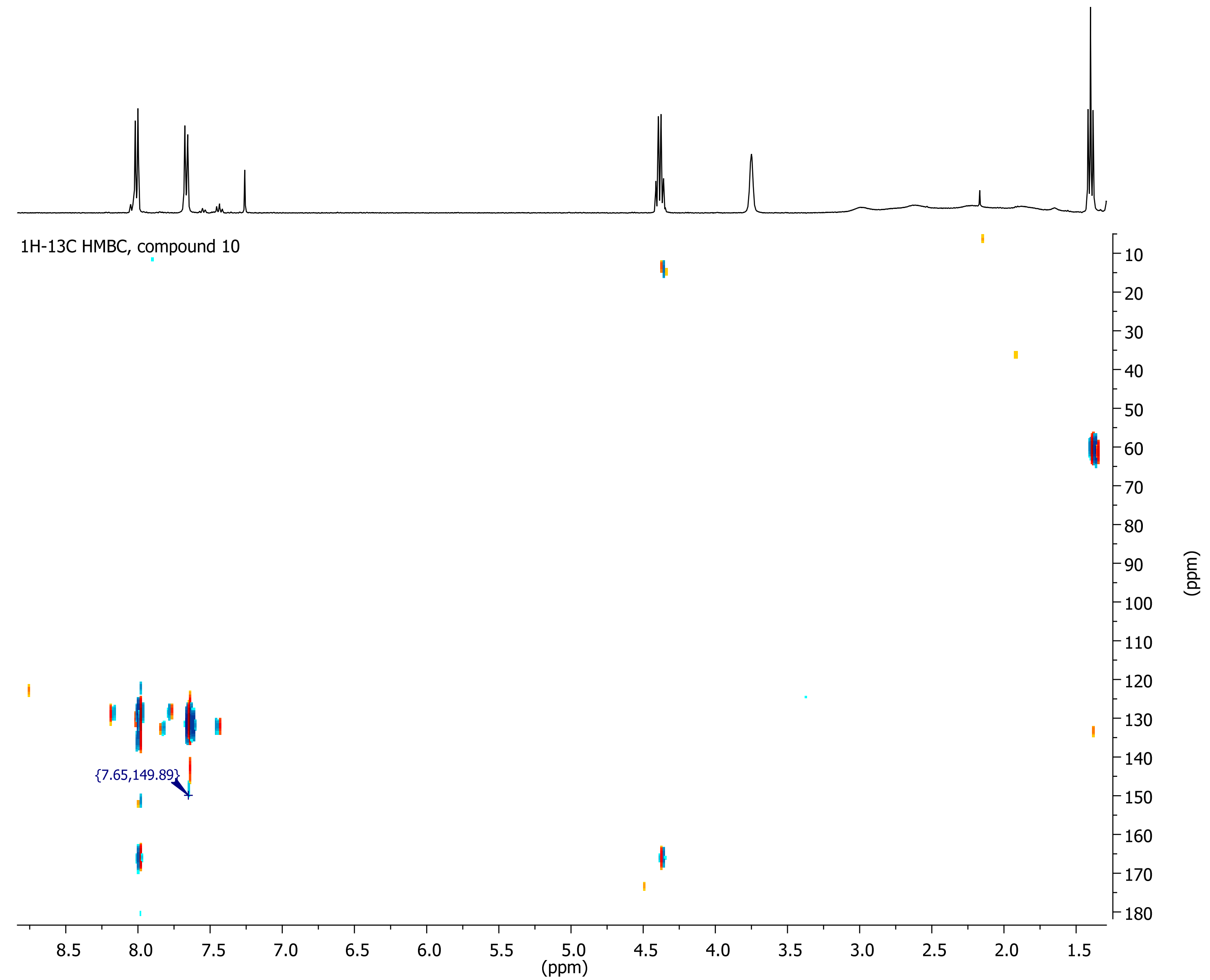
1H-13C HMBC, compound 10
{7.65,149.89}
8.5
8.0
7.5
7.0
6.5
6.0
5.5
5.0
(ppm)
4.5
4.0
3.5
3.0
2.5
2.0
1.5
10
20
30
40
50
60
70
80
90
100
110
120
130
140
150
160
170
180
(ppm)

Calculated isotope distribution for C11H19B10O2 (M-H)-
Compound 10

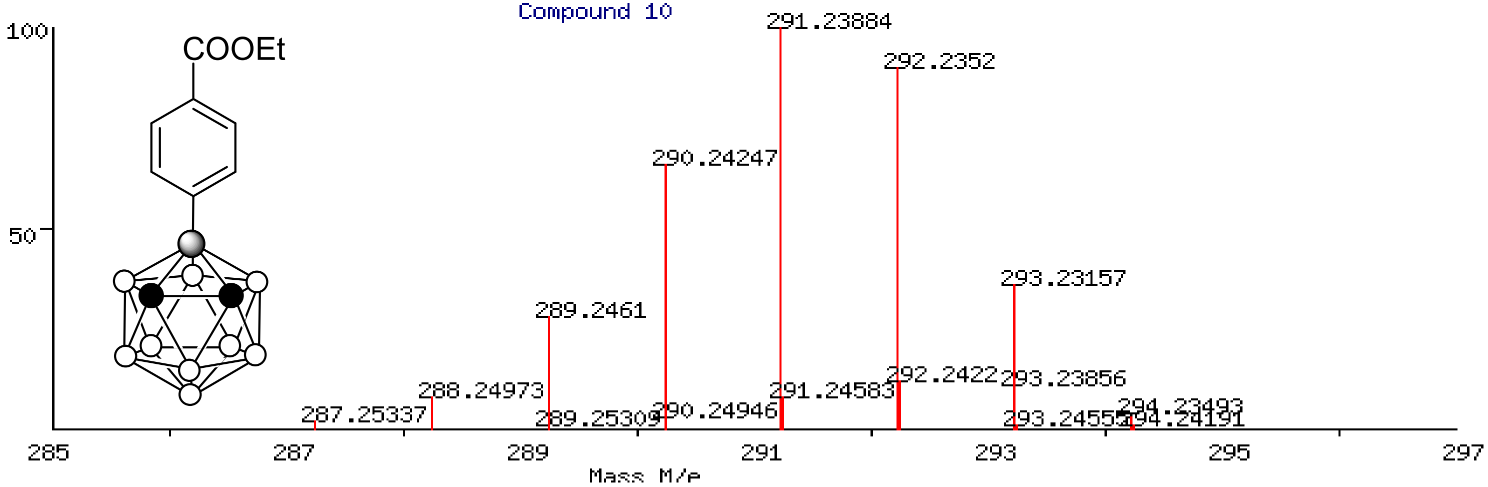

Compound 10, negative ion mode

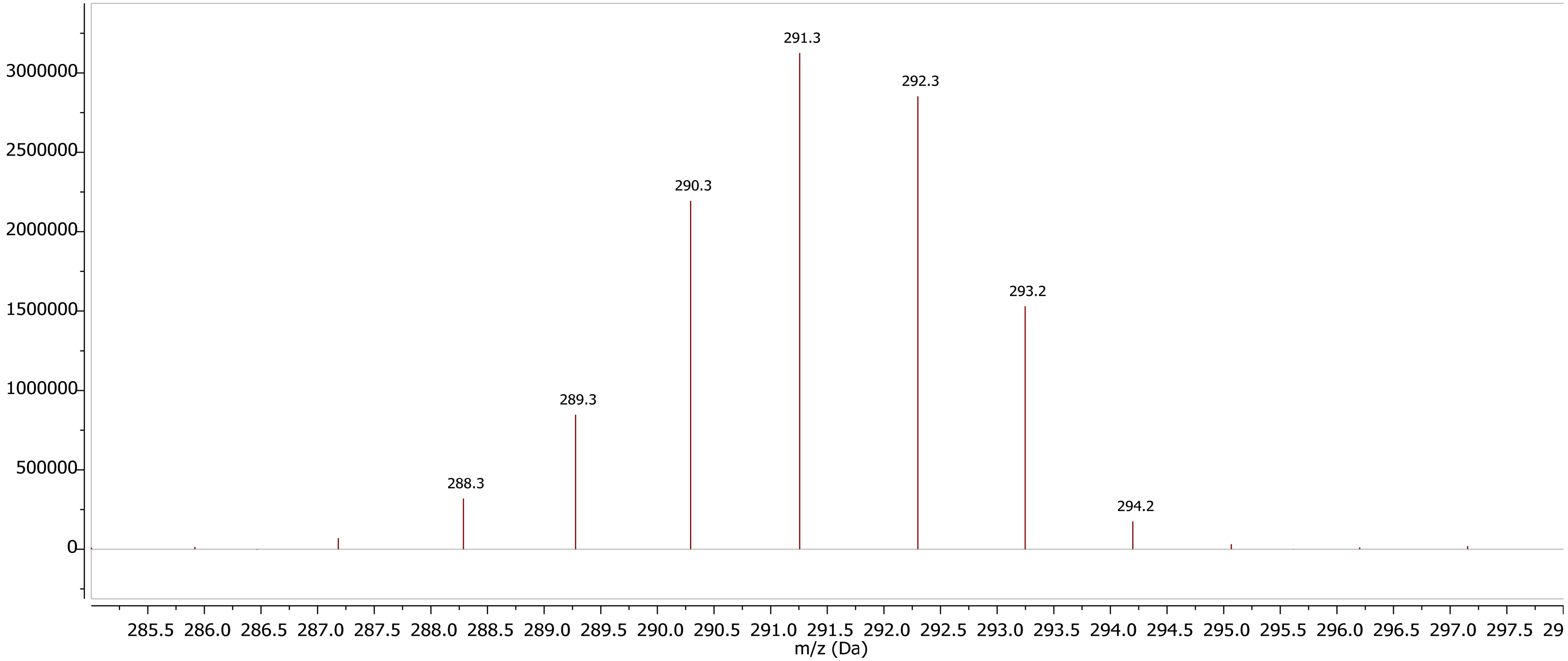

Supplement: Supplementary file 1 [file molecules-26-07297-s001.zip › molecules-1467975-supplementary.pdf]
